# Serum amyloid A expression in liver promotes synovial macrophage activation and chronic arthritis via NFAT5

**DOI:** 10.1172/JCI167835

**Published:** 2024-03-01

**Authors:** Meiling Li, Yu-Mi Kim, Jung Hee Koh, Jihyun Park, H. Moo Kwon, Jong-Hwan Park, Jingchun Jin, Youngjae Park, Donghyun Kim, Wan-Uk Kim

**Affiliations:** 1 Center for Integrative Rheumatoid Transcriptomics and Dynamics,; 2 Department of Biomedicine and Health Sciences, and; 3 Department of Internal Medicine, College of Medicine, The Catholic University of Korea, Seoul, Republic of Korea.; 4 Department of Immunology of Yanbian University Hospital, Yanji, Jilin Province, China.; 5 Division of Rheumatology, Department of Internal Medicine, Uijeoungbu St.Mary’s hospital, the Catholic University of Korea, Uijeoungbu, Republic of Korea.; 6 Department of Biomedical Sciences, Seoul National University College of Medicine, Seoul, Republic of Korea.; 7 School of Nano-Bioscience and Chemical Engineering, Ulsan National Institute of Science and Technology, Ulsan, Republic of Korea.; 8 Department of Laboratory Animal Medicine, College of Veterinary Medicine, Chonnam National University, Gwangju, Republic of Korea.; 9 Key Laboratory of Science and Technology Department (Jilin Province), Cancer Research Center, Yanji, Jilin Province, China.; 10 Department of Microbiology and Immunology, Seoul National University College of Medicine, Seoul, Republic of Korea.; 11 Institute of Endemic Diseases, Seoul National University Medical Research Center, Seoul, Republic of Korea.

**Keywords:** Autoimmunity, Inflammation, Arthritis, Autoimmune diseases, Rheumatology

## Abstract

Nuclear factor of activated T-cells 5 (NFAT5), an osmo-sensitive transcription factor, can be activated by isotonic stimuli, such as infection. It remains unclear, however, whether NFAT5 is required for damage-associated molecular pattern–triggered (DAMP-triggered) inflammation and immunity. Here, we found that several DAMPs increased NFAT5 expression in macrophages. In particular, serum amyloid A (SAA), primarily generated by the liver, substantially upregulated NFAT5 expression and activity through TLR2/4-JNK signalling pathway. Moreover, the SAA-TLR2/4-NFAT5 axis promoted migration and chemotaxis of macrophages in an IL-6– and chemokine ligand 2–dependent (CCL2-dependent) manner in vitro. Intraarticular injection of SAA markedly accelerated macrophage infiltration and arthritis progression in mice. By contrast, genetic ablation of NFAT5 or TLR2/4 rescued the pathology induced by SAA, confirming the SAA-TLR2/4-NFAT5 axis in vivo. Myeloid-specific depletion of NFAT5 also attenuated SAA-accelerated arthritis. Of note, inflammatory arthritis in mice strikingly induced SAA overexpression in the liver. Conversely, forced overexpression of the SAA gene in the liver accelerated joint damage, indicating that the liver contributes to bolstering chronic inflammation at remote sites by secreting SAA. Collectively, this study underscores the importance of the SAA-TLR2/4-NFAT5 axis in innate immunity, suggesting that acute phase reactant SAA mediates mutual interactions between liver and joints and ultimately aggravates chronic arthritis by enhancing macrophage activation.

## Abstract 2

Serum amyloid A, an acute-phase protein, mediates inter-organ communication between liver and joints and thereby promotes synovial macrophage activation via TLR2/4-NFAT5 axis to exacerbate chronic arthritis.

## Graphical abstract

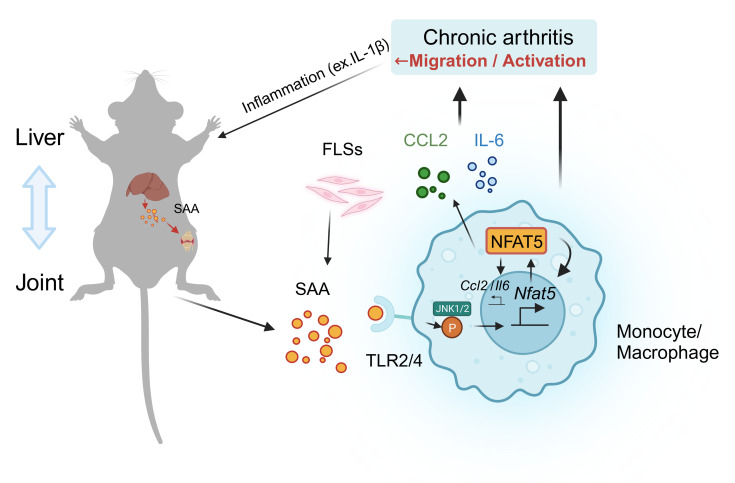

## Introduction

Macrophages play a pivotal role in the pathogenesis of chronic inflammatory diseases, such as rheumatoid arthritis (RA), with broad proinflammatory, destructive, and remodeling capabilities (1, 2). Similar to other immune cells, macrophages expand their functions by placing the transcriptional machinery within a distinct signaling pathway. A number of transcription factors and signal regulators in macrophages have been identified, including NF-κB, p53, phosphoinositide 3-kinase, and mitogen-activated protein kinases (MAPKs) (3, 4). Recently, through global transcriptome profiling, we demonstrated that nuclear factor of activated T-cells 5 (NFAT5) is one of the critical regulators for a wide range of pathologic processes mediated by macrophages, including cell proliferation, apoptosis, cytokine production, and chemotaxis; indeed, its pathologic significance in RA macrophages is comparable to that of NF-κB and p53 (5).

NFAT5 is a member of the Rel family of transcription factors that shares a conserved DNA binding domain with NFATc1–4 (6). It was originally identified as a tonicity-responsive enhancer-binding protein involved in the protection of cells, such as kidney medulla epithelia and skin epidermis, from hypertonic stress (7, 8). We and others have uncovered that, independent of hypertonicity, NFAT5 regulates the expression of a number of proinflammatory genes in macrophages upon Toll-like receptor (TLR) ligation with lipopolysaccharide (LPS) (9, 10). During infection and inflammation, macrophages are exposed to a panoply of endogenous TLR ligands, including serum amyloid A (SAA), myeloid-related protein 8 (MRP8), and high-mobility group box 1 (HMGB1) (11, 12). It is therefore plausible that endogenous TLR ligands also activate macrophages to induce NFAT5 expression, which may lead to diverse pathologic phenotypes. Little is known, however, about links between endogenous TLR ligands and NFAT5.

In response to infection and inflammation, innate immune cells secrete proinflammatory cytokines, in particular IL-1β and TNF-α, to which the liver responds by producing acute-phase reactants. A large body of evidence suggests that acute phase reactants, such as SAA, C-reactive protein, and α1-acid glycoprotein, actively participate in immune responses and regulation of chronic inflammation and thus go beyond their role as diagnostic markers (11, 13). For example, SAA is an endogenous TLR ligand functioning as a damage-associated molecular pattern (DAMP) that responds to bacterial endotoxins (14). SAA is strongly chemotactic for neutrophils and macrophages (15), induces production of proinflammatory cytokines (16), and promotes synoviocyte hyperplasia in RA (17, 18). Further identification of such pathologic process by acute-phase reactants will allow for better selection of therapeutic targets as well as a greater understanding of the mechanisms underlying chronic inflammation.

Here, we propose the intriguing hypothesis that SAA, an acute phase reactant as well as a TLR ligand, activates NFAT5 in macrophages of arthritic joints after being secreted from the liver and thereby promotes chronic inflammation. Using *Nfat5*- and *Tlr*2/4-deficient mice, we show that SAA can trigger the TLR2/4 receptor to upregulate NFAT5 expression, culminating in increased migration of macrophages and aggravation of chronic arthritis. Strikingly, forced overexpression of SAA gene in the liver exacerbates inflammatory arthritis in mice, which, in turn, upregulates SAA expression in the liver. Collectively, our data suggest that acute phase reactant SAA mediates interorgan communication between liver and joints and ultimately enhances macrophage activation and chronic arthritis via osmo-sensitive transcription factor NFAT5.

## Results

### SAA increases NFAT5 expression and activity in monocytes and macrophages.

**Figure 1:**
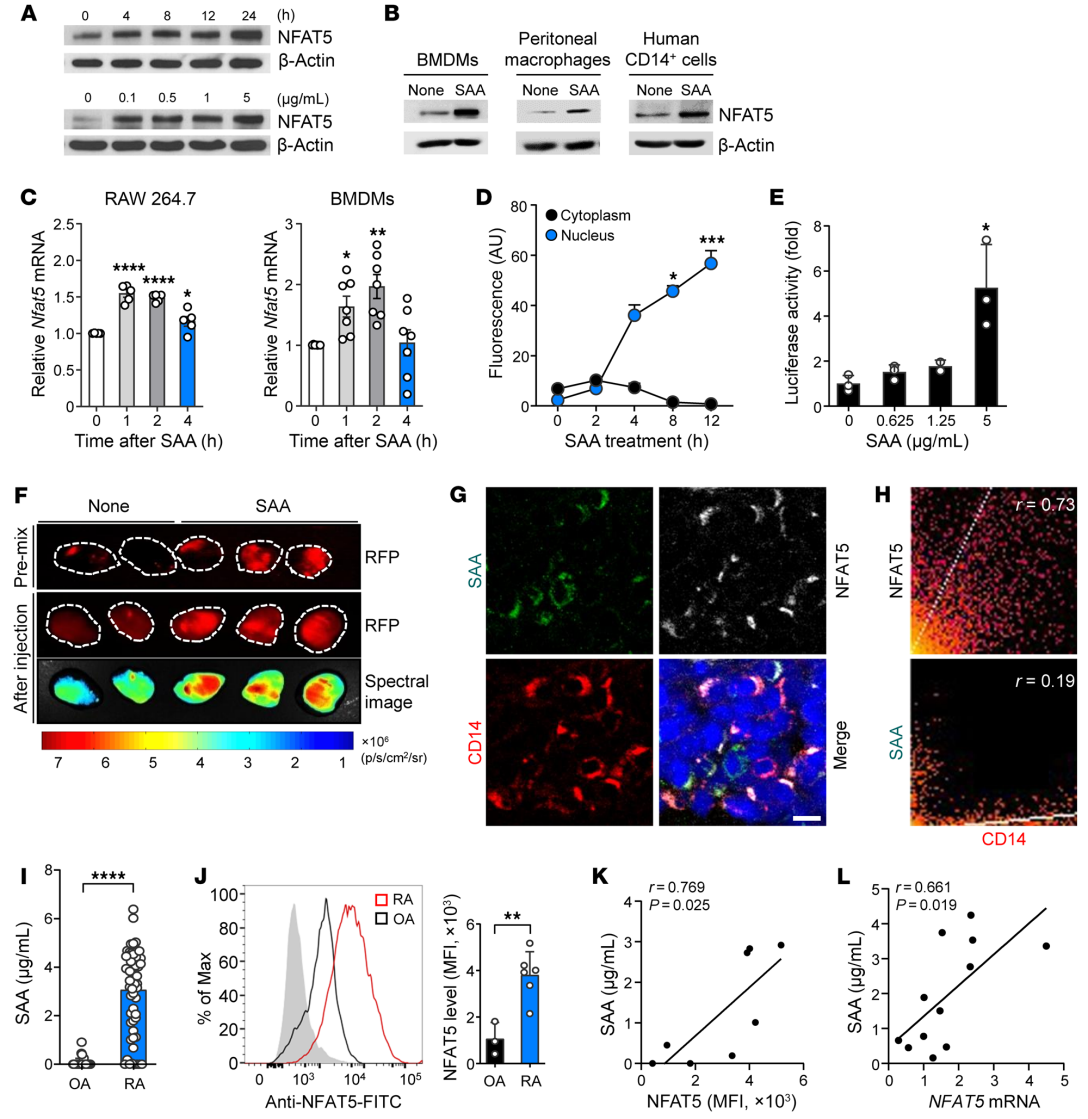
SAA-induced upregulation of NFAT5 expression and activity in macrophages. (**A** and **B**) Western blot analysis of NFAT5 in (**A**) RAW 264.7 macrophages, (**B**) primary mouse macrophages (BMDMs, left; peritoneal macrophages, middle) and human peripheral CD14^+^ monocytes (right) after SAA (5 μg/mL) treatment. β-actin served as a loading control. Data are representative of at least 3 independent experiments. (**C**) *Nfat5* mRNA expression levels in RAW264.7 cells and BMDMs after treatment with SAA (5 μg/mL). For qRT-PCR, *Gapdh* mRNA was used as an internal control. (**D**) Time course of NFAT5 localization in RAW 264.7 macrophages (*n* = 5) treated with SAA. NFAT5 expression was determined by immunoflorescent staining and is shown as fluorescence intensity. (**E**) Luciferase reporter assays for NFAT5-dependent promotor activity in RAW 264.7 macrophages treated with SAA for 24 hours after transduction of luciferase constructs (*n* = 3). (**F**) In vivo analysis of NFAT5 reporter activity in mice implanted with matrigel containing RAW 264.7 macrophages transfected with NFAT5-RFP reporter. SAA (10 μg/mL) was premixed (top) with matrigel or injected s.c. into the mice (middle and bottom). Representative images are shown. (**G** and **H**) Triple immunofluorescence staining of RA synovium for CD14 (red), NFAT5 (white), and SAA (green). Nuclei were counterstained with DAPI (blue). Scale bars: 10 μm. Data representative of more than 3 patients with RA is shown (**G**). Significance of colocalization was assessed by Pearson’s correlation coefficient analysis (**H**). (**I**) SAA concentrations in synovial fluids (SF) of patients with RA (*n* = 60) and OA (*n* = 33), as determined by ELISA. (**J**) Flow cytometry analysis of NFAT5 expression in CD14^+^ cells freshly isolated from the SF of patients with RA (*n* = 6) and OA (*n* = 3). (**K** and **L**) Correlation between SAA concentrations measured by ELISA and NFAT5 expression levels in CD14^+^ cells by flow cytometry (**K**) or qRT-PCR (**L**) in SF of patients with RA. Data in (**C**–**E**, and **J**) are mean ± SD. *P < 0.05, **P < 0.01, ***P < 0.001, and ****P < 0.0001 by 1-way ANOVA with Dunn’s multiple comparisons test (**C**), Friedman’s test with Dunn’s multiple comparisons test (**D**), Kruskal-Wallis test with Dunn’s multiple comparisons test (**E**), Mann Whitney U test (**I**), unpaired t test (**J**), and Pearson correlation test (**K** and **L**).

We and others have demonstrated that pathogen-associated molecular patterns (PAMPs) increase expression of NFAT5 and its target genes in a tonicity-independent manner (9, 10). However, it remains unknown whether DAMPs activate monocytes and macrophages to induce NFAT5. To address this, we added several DAMPs, including SAA, MRP8, and HMGB1, to cultured RAW 264.7 macrophages. As seen in Figure 1A and Supplemental Figure 1, A and B (supplemental material available online with this article; https://doi.org/10.1172/JCI167835DS1), SAA, MRP8, and HMGB1 all substantially increased NFAT5 expression of RAW 264.7 macrophages in dose- or time-dependent manners. The SAA-induced NFAT5 increase was reproduced in primary cells, including mouse bone marrow–derived macrophages (BMDM), mouse peritoneal macrophages, and human CD14^+^ peripheral monocytes (Figure 1B), suggesting that DAMPs (particularly SAA), like PAMPs (12), can induce NFAT5 expression in monocytes and macrophages.

Similar to other acute-phase proteins, SAA is produced predominantly by hepatocytes in response to injury, infection, and inflammation (19). Beyond its upregulatory effect on NFAT5 expression, we further investigated whether SAA also affected NFAT5 activity and transcription of target genes. We found a rapid increase in NFAT5 mRNA expression in RAW264.7 cells and BMDMs following treatment with 5 μg/mL SAA, with peak expression at 1 to 2 hours after SAA treatment (Figure 1C). As early as 4 hours after stimulation with SAA, NFAT5 translocation from the cytoplasm to the nucleus occurred in RAW 264.7 macrophages on confocal microscopy, and its increase was time dependent (Figure 1D and Supplemental Figure 2A). In addition, luciferase assay revealed that NFAT5-dependent reporter activity was also markedly elevated by SAA (5 μg/mL) (Figure 1E), which suggests that SAA promotes mRNA transcription of the NFAT5-dependent target genes. Together, SAA seems to enhance NFAT5 activity in at least 2 aspects: increased promoter activity and nuclear translocation.

To confirm this in vivo, we implanted matrigel containing SAA and RAW 264.7 macrophages transfected with NFAT5-dependent red fluorescence protein (RFP) reporter into mice (Supplemental Figure 1C). When the cells were stimulated locally with SAA in vivo for 16 hours, a strong RFP signal was observed in the matrigel harboring RAW 264.7 macrophages plus SAA (10 μg/mL), but not in those with no SAA (top panel of Figure 1F). Such increase was similarly noted with 40 μg/mL of recombinant MRP8, another DAMP (Supplemental Figure 1D). We also administered SAA (20 μg/mL) systemically (i.p.) 9 days after implantation of cell-containing matrigel, when new vessels had grown inside the matrigel, and confirmed that this could increase the RFP signal in vivo (middle and bottom panels of Figure 1F). Addition of polymyxin B also failed to inhibit NFAT5 upregulation by SAA while it suppressed LPS-induced NFAT5 upregulation (Supplemental Figure 3A), which eliminates concerns of possible endotoxin contamination.

To demonstrate the pathologic relevance of SAA upregulation of NFAT5, we sought to determine SAA and NFAT5 expression levels in patients with RA, a representative chronic inflammatory disease where monocytes and/or macrophages play a major role (20). On immunostaining of RA synovium, most of the CD14^+^ synovial macrophages highly expressed NFAT5 but failed to express SAA (Figure 1G); the correlation coefficient for colocalization analysis was 0.73 between CD14 and NFAT5 and 0.19 between CD14 and SAA (Figure 1H). Rather, SAA was predominantly expressed in fibroblast-like cells (FLSs) adjacent to NFAT5-expressing macrophages (Figure 1G and data not shown). As reported previously (21, 22), mean levels of SAA were found to be much greater (50.3-fold) in the synovial fluids of patients with RA than in those of patients with osteoarthritis (OA), a noninflammatory control (Figure 1I, mean level: 3.1 ± 1.7 versus 0.1 ± 0.2 μg/mL, *P* < 0.001). Moreover, NFAT5 expression in macrophages and monocytes, which were freshly isolated from synovial fluids, was also higher in patients with RA than patients with OA (Figure 1J), correlating well with SAA concentrations in the synovial fluids of patients with RA (Figure 1, K and L). Collectively, these data demonstrate that several DAMPs, particularly acute phase reactant SAA, increases NFAT5 expression and activity in monocytes/macrophages.

### TLR2/4-JNK signaling pathway primarily mediates SAA-induced NFAT5 expression.

**Figure 2:**
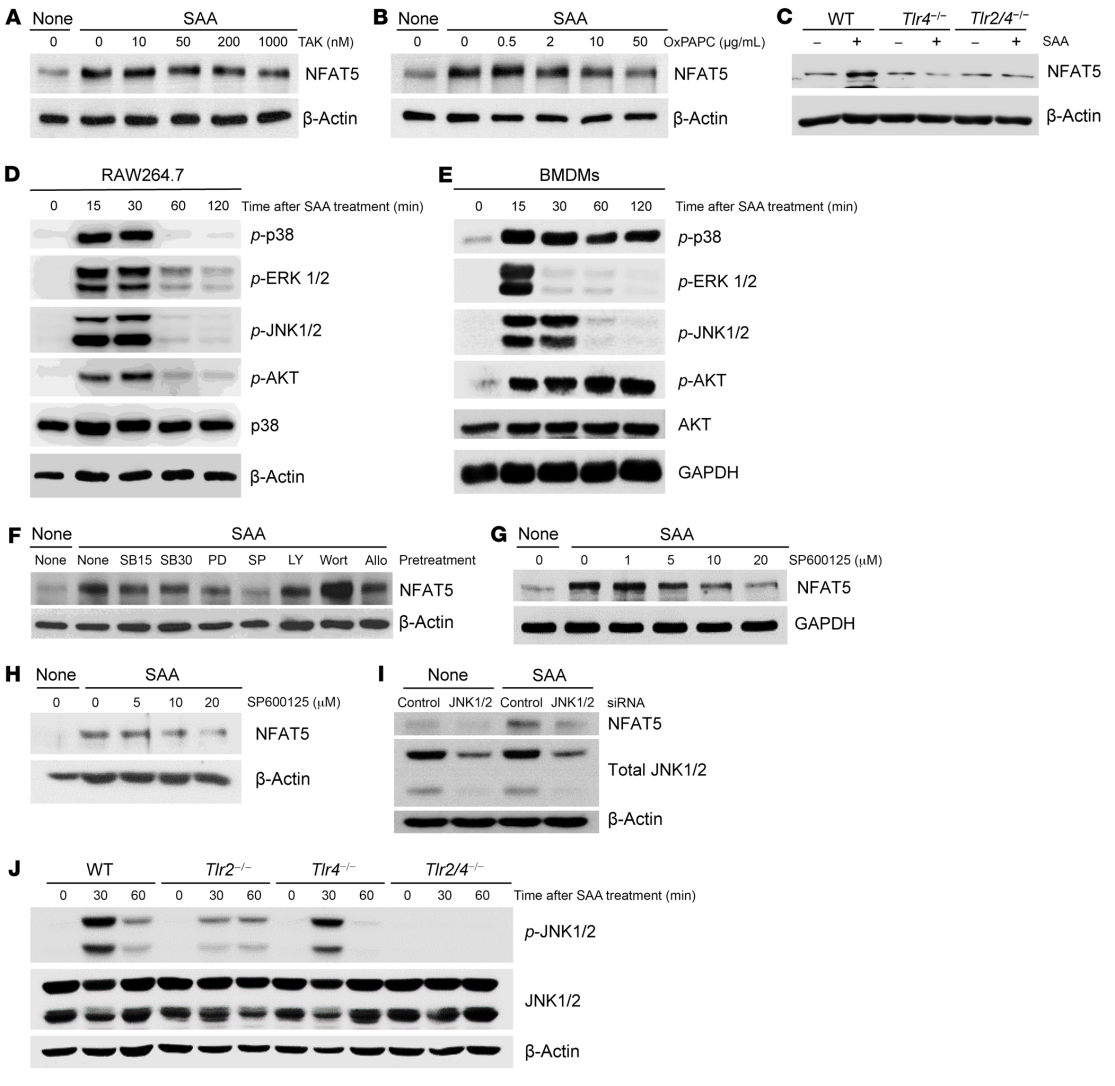
SAA upregulation of NFAT5 expression via TLR2/4-JNK pathway in macrophages. (**A** and **B**) Western blot analysis for NFAT5 expression in RAW 264.7 macrophages treated with TAK-242 (TAK) or oxidized 1-palmitoyl-2-arachidonoyl-sn-glycero-3-phosphorylcholine (OxPAPC) in the presence of SAA. (**C**) NFAT5 expression in SAA-treated BMDMs of WT, TLR4 KO (*Tlr4*^–/–^) and TLR2/4 double KO (*Tlr2/4*^–/–^) mice. (**D** and **E**) Increase in phospho-p38 (*p*-p38), phospho-ERK (*p*-ERK1/2), phospho-JNK1/2 (*p*-JNK1/2), and phospho-AKT (*p*-AKT) levels by SAA (5 μg/mL) in RAW 264.7 macrophages (**D**) and mouse BMDMs (**E**), as determined by Western blot. (**F**) NFAT5 expression in RAW 264.7 macrophages pretreated with SB203580 (SB, 15 [SB15] and 30 μM [SB30]), PD98059 (PD, 30 μM), SP600125 (SP, 10 μM), LY294002 (LY, 10 μM), Wortmannin (Wort, 10 μM), and allopurinol (Allo, 1 mM) for 1 hour. (**G** and **H**) SP600125 inhibition of NFAT5 expression in RAW 264.7 macrophages (**G**) and mouse BMDMs (**H**) stimulated with SAA. (**I**) Reduction of NFAT5 expression in RAW 264.7 macrophages by *Jnk1/2* siRNA. Cells were transfected with control or *Jnk1/2* siRNA for 24 hours and then treated with SAA (5 μg/mL) for 8 hours. Expression of NFAT5 and total JNK1/2 was measured by Western blot analysis. (**J**) Decrease in *p*-JNK1/2 expression by *Tlr2/4* deficiency. BMDMs of WT, *Tlr2*^–/–^, *Tlr4*^–/–^, *Tlr2/4*^–/–^ mice were activated with SAA for the indicated times. Total JNK1/2 and *p*-JNK1/2 expressions were determined by Western blotting. Data are representative of more than 3 independent experiments with similar results. In experiments for **A**, **B**, **C**, **F**, **G**, and **H**, the cells were stimulated with 5 μg/mL of SAA for 24 hours.

Most transcription factors have independent roles and outcomes depending on the signaling pathway. We next questioned how SAA transmits an activation signal for NFAT5 induction in cells. Several receptors are known to mediate the effect of SAA, including formyl peptide receptor-like 1 (FPRL1), receptor for advanced glycation end-products (RAGE), TLR2, and TLR4 (19). As shown in Supplemental Figure 3B, pertussis toxin and toxin B, the inhibitors of G-protein–coupled receptors (23, 24) including FPRL1, failed to reduce SAA-stimulated NFAT5 expression in RAW 264.7 macrophages. Moreover, W peptide, a specific agonistic peptide for FPRL1 (24), did not affect NFAT5 expression (Supplemental Figure 3C), suggesting that SAA does not utilize FPRL1 for NFAT5 production. By contrast, TAK-242 (a TLR4 signaling antagonist) and oxidized 1-palmitoyl-2-arachidonoyl-sn-glycero-3-phosphorylcholine (OxPAPC, a TLR2/4 antagonist) dose-dependently inhibited the SAA-induced increase in NFAT5 expression (Figure 2, A and B). In parallel, in *Tlr*4- or *Tlr*2/4-deficient (–/–) mouse peritoneal macrophages, SAA failed to increase NFAT5 expression (Figure 2C). Therefore, TLR2/4 appears to be essential to SAA-induced NFAT5 upregulation in macrophages.

TLR2/4 propagates its signal via MAPKs, which include p38, extracellular signal–regulated kinase (ERK), and c-Jun NH 2-terminal kinase (JNK) (25). To assess whether the 3 MAPKs contribute to the SAA-TLR2/4–induced NFAT5 activation in macrophages, we treated cells with SAA in the presence of chemical inhibitors specific for each type of MAPK. As expected, it was found that SAA treatment strongly increased phosphorylation of p38, ERK, JNK 1/2, and AKT in RAW 264.7 macrophages and mouse BMDM (Figure 2, D and E). JNK inhibitor SP600125 completely inhibited SAA-induced NFAT5 expression in RAW 264.7 macrophages, whereas PD98059 and SB203580, which act on ERK1/2 and p38 MAPK, respectively, only partially restricted it (Figure 2F). SP600125 inhibition in RAW 264.7 cells was dose-dependent (Figure 2G), and it was reproduced in BMDM (Figure 2H). By contrast, LY294002 and wortmannin (inhibitors of phosphatidylinositol-3-kinase/AKT pathway) failed to mitigate NFAT5 upregulation by SAA (Figure 2F), indicating that JNK, but not Akt, is the major signal mediating NFAT5 upregulation by SAA. In support, *Jnk1/2* siRNAs completely repressed the SAA upregulation of NFAT5 in RAW 264.7 cells (Figure 2I). Moreover, in mouse BMDM, either *Tlr2* or *Tlr4* KO alone, only partially reduced SAA-stimulated expression of p-JNK1/2, but knocking out both completely abolished it (Figure 2J), indicating that both TLR2 and TLR4 are required for SAA-induced JNK activity. Collectively, the data suggest that SAA upregulates NFAT5 expression in macrophages via the TLR2/4-JNK signaling pathway.

### SAA-induced increase in macrophage migration is dependent on NFAT5.

**Figure 3:**
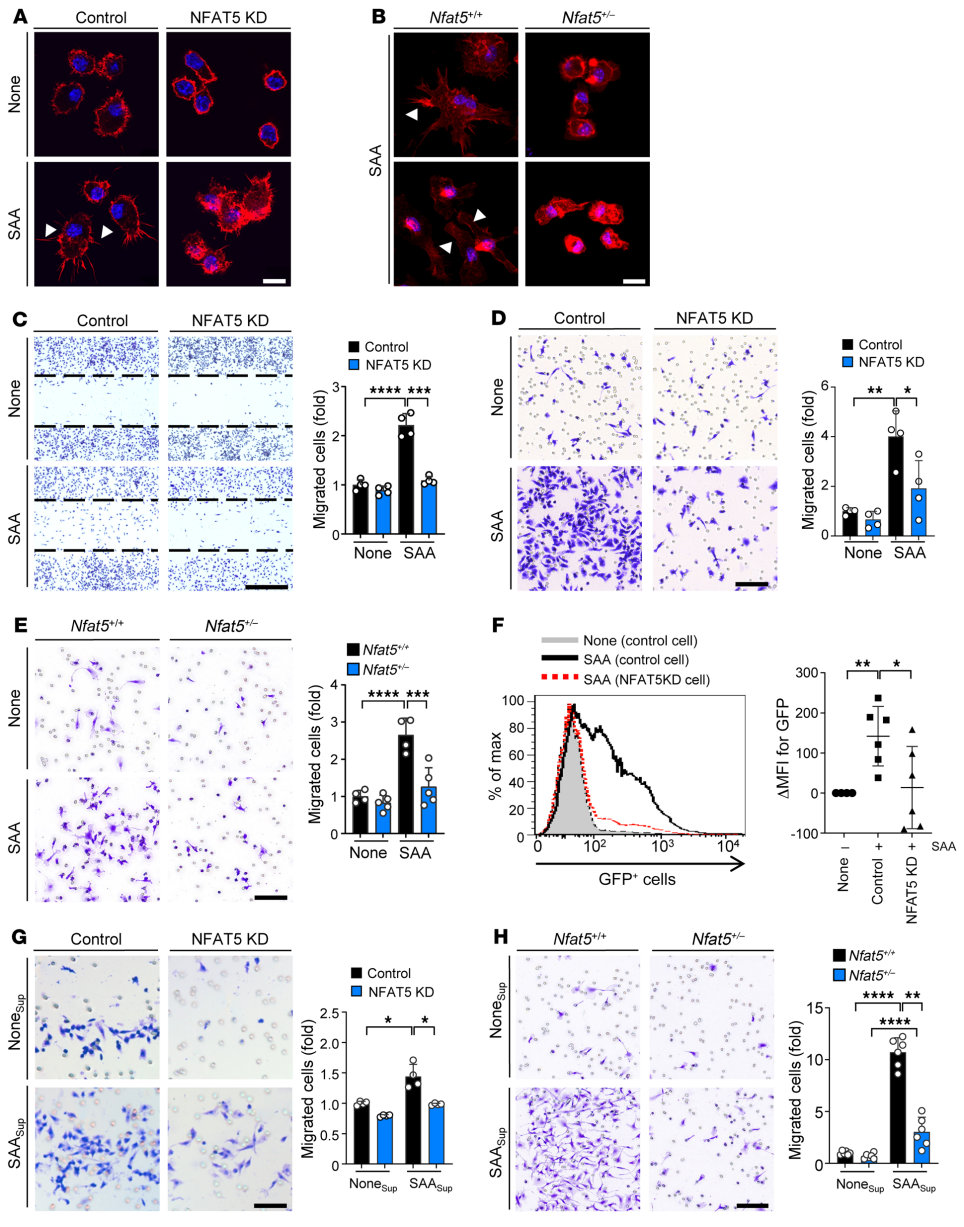
Requirement of NFAT5 for SAA-induced macrophage migration. (**A** and **B**) Immunocytochemical analysis of F-actin (red) in RAW 264.7 macrophages transfected with NFAT5 shRNA (**A**) and peritoneal macrophages of *Nfat5*^+/–^ mice (**B**), stimulated with SAA (2.5 μg/mL) for 2.5 hours. Nuclei were counterstained with DAPI (blue). Arrows indicate filopodia and lamellipodia. Scale bars: 10 μm. (**C**) Decrease in SAA-induced wound migration of RAW 264.7 macrophages by NFAT5 knockdown (KD). Scale bar: 400 μm. (**D** and **E**) Decrease in transwell migration of NFAT5 KD RAW 264.7 macrophages (**D**) and *Nfat5*^+/–^ BMDM (**E**) stimulated with SAA (5 μg/mL). Scale bars: 500 μm. (**F**) Macrophage migration in SAA-stimulated air pouch in vivo. After air pouches were established in BALB/c mice, the mice were subjected to i.v. injection of NFAT5 KD or control RAW 264.7 cells (1 × 10^5^) labeled with GFP. After 48 hours, the ratio of GFP^+^ cells migrating to the air pouch cavity containing SAA (20 μg/mL) were evaluated by flow cytometry. (**G** and **H**) Chemotactic migration of macrophages to conditioned media. Conditioned media (SAA_Sup_) were harvested from *Nfat5*-deficient (KD) or -sufficient RAW 264.7 macrophages (**G**) and BMDM (**H**) 24 hours after stimulation with SAA (5 μg/mL). Scale bars: 100 μm. Data represent mean ± SD. *P < 0.05, **P < 0.01, ***P < 0.001, and ****P < 0.0001 by Brown–Forsythe and Welch’s ANOVA (**C**, **G**, and **H**), 1-way ANOVA test (**D** and **E**), and Kruskal-Wallis test (**F**). Comparison of numerical data between groups were performed using the unpaired t test, Welch’s t test, or Mann-Whitney U test.

It is well known that SAA provokes monocyte chemotaxis by inducing several chemokines, including CCL2, MIP-1, and IL-8 (26). Moreover, NFAT5 promotes migration and/or invasion of some types of cells, including cancer cells, macrophages, and synovial fibroblasts (27–29). However, an answer to whether the SAA-induced increase in cell migration is dependent on NFAT5 remains elusive. To explore the functional role of SAA-TLR2/4-NFAT5 signaling in macrophages, we first tested whether NFAT5 deficiency reduced chemotactic migration of RAW 264.7 macrophages on SAA stimulation. The assembly of actin-based extensions, namely filopodia and lamellipodia, is an integral component of cell migration (30). As reported previously (31), SAA treatment strongly increased filopodia formation in RAW 264.7 macrophages, which was markedly hampered by stable knockdown of NFAT5 transcripts (NFAT5 KD) using short hairpin RNAs (shRNAs) (Figure 3A and Supplemental Figure 4, A and B); this increase was not affected by polymyxin B, excluding endotoxin contamination (data not shown). NFAT5 haplodeficient (*Nfat5*^+/–^) peritoneal macrophages also showed a substantial decrease in filopodia and lamellipodia formation, along with a reduction of NFAT5 expression in the presence of SAA (Figure 3B and Supplemental Figure 4C); and such reduction was similarly noted when the cells were stimulated with MRP8, another DAMP (Supplemental Figure 5, A and B).

In parallel, SAA-induced migration was much lower in *Nfat5*-deficient RAW 264.7 cells or BMDM than in *Nfat5*-sufficient control cells, as determined by wound migration assay (Figure 3C) and Boyden chamber assay (Figure 3, D and E and Supplemental Figure 6A), thus demonstrating that the SAA/NFAT5 axis controls migration of macrophages. To validate this in vivo, we used the mouse air pouch model of inflammation and measured the recruitment of *Nfat5*-deficient versus -sufficient macrophages into air pouches harboring SAA. After i.v. injection of RAW 264.7 cells labeled with GFP, the number of GFP^+^ cells in the pouch was increased by addition of SAA (20 μg/mL) to the air pouch, an effect that was markedly reduced by knockdown of NFAT5 (Figure 3F). Together, these observations demonstrate that SAA/NFAT5 axis controls macrophage migration in vitro and in vivo possibly by expediting filopodia and lamellipodia formation.

Since SAA activates macrophages to produce a variety of cytokines and chemokines, we wondered if the promigratory activity of the SAA/NFAT5 axis is achieved by these mediators. As shown in Figure 3G and Supplemental Figure 6B, conditioned media from NFAT5-knockdown cells stimulated with or without SAA, were less effective in promoting migration of RAW 264.7 macrophages than those from control cells. Conditioned media of *Nfat5*^+/–^ macrophages showed similar results (Figure 3H).

**Figure 4:**
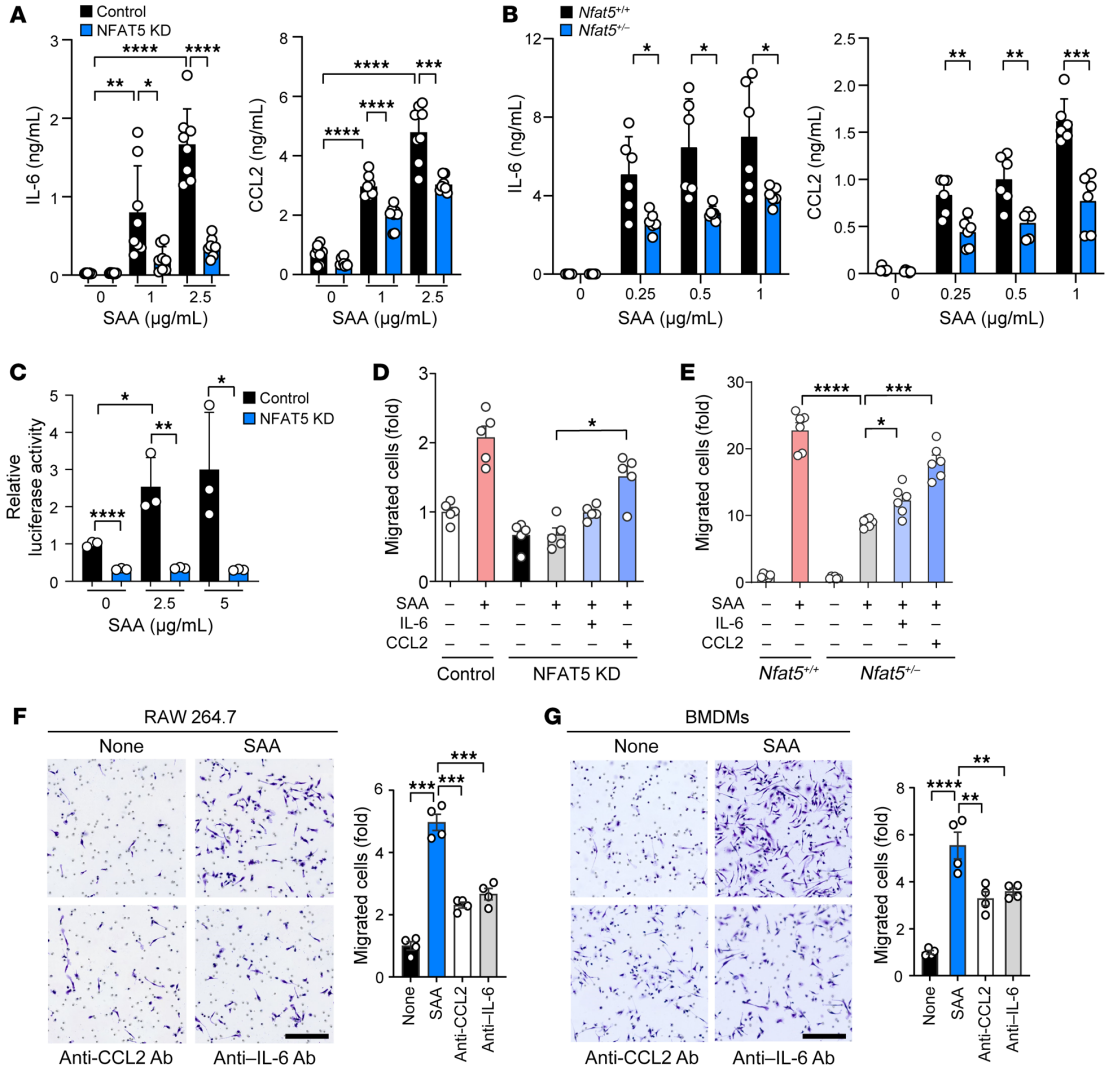
CCL2 and IL-6 reversal of macrophage migration repressed by *Nfat5* deficiency. (**A** and **B**) Reduced IL-6 and CCL2 production by NFAT5 KD RAW 264.7 macrophages and *Nfat5*^+/–^ BMDM, as determined by ELISA. The cells were stimulated with SAA for 24 hours. IL-6 and CCL2 concentrations in the culture supernatants were determined by ELISA. (**C**) Decrease in SAA-stimulated CCL2 promoter activity by the knockdown of NFAT5. NFAT5 KD or control RAW 264.7 macrophages (1 × 10^5^) were transfected with CCL2 reporter plasmid (250 ng/well), and the transfected cells were then treated with the indicated concentrations of SAA for 18 hours. The CCL2 promoter activity was determined by luciferase assay. (**D** and **E**) Recovery effect of CCL2 and IL-6 on the decrease in macrophage migration by *Nfat5* deficiency. In the same experimental conditions as **A** and **B**, recombinant CCL2 (10 ng/mL) or IL-6 (10 ng/mL) were added to NFAT5 KD RAW 264.7 cells (**D**) and *Nfat5*^+/–^ BMDM (**E**). After 8 hours, the cell migration was determined by Boyden chamber assay. (**F** and **G**) Inhibition of macrophage migration by neutralizing Ab to CCL2 or IL-6. Anti-CCL2 Ab (1 μg/mL) or anti-IL-6 Ab (1 μg/mL) was mixed for 1 hour with the conditioned media of RAW264.7 cells (**F**) and BMDM (**G**), which were stimulated with SAA for 24 hours. Macrophage migration was assessed in a Boyden chamber 8 hours after the treatment of conditioned media in the absence or presence of the blocking Abs. Scale bars: 150 μm. Data represent mean ± SD. *P < 0.05, **P < 0.01, ***P < 0.001, and ****P < 0.0001 by Brown–Forsythe and Welch’s ANOVA (**A** and **E**), 1-way ANOVA test (**D**, **F**, and **G**), and Kruskal-Wallis test (**B**). Comparison of numerical data between groups were performed using the unpaired t test, Welch’s t test, Mann-Whitney U test or Tukey’s multiple comparisons test.

We and others have reported that NFAT5 regulates production of a number of cytokines and chemokines by macrophages, and, in particular, IL-6 and CCL2 (20, 32). In accordance with earlier reports (16), SAA-stimulated production of IL-6 and CCL2 was much lower in *Nfat5*-deficient RAW 264.7 cells or BMDM than in *Nfat5*-sufficient control cells (Figure 4, A and B), suggesting that NFAT5 is critical to IL-6 and CCL2 secretion by SAA; meanwhile, CCR2, the CCL2 receptor, expression was not affected by SAA and showed no difference between *Nfat5*-deficient and sufficient RAW 264.7 cells, as determined by flow cytometry (data not shown). Moreover, in the CCL2 promoter-reporter assay, luciferase activity showed a dose-dependent increase when stimulated by SAA in NFAT5-sufficient (RAW 264.7) macrophages, but this response was not observed in NFAT5-knockdown macrophages, which indicates that CCL2 expression was regulated through the direct binding of NFAT5 to the CCL2 promoter (Figure 4C). Of note, the decrease in macrophage migration was partially recovered when recombinant CCL2 or IL-6 was added to the Boyden chamber (Figure 4, D and E). Moreover, the addition of neutralizing antibody (Ab) to CCL2 or to IL-6 in the supernatant of SAA-treated macrophages remarkably reduced the chemotactic migration of both RAW 264.7 cells and BMDM (Figure 4, F and G). These results support the suggestion that SAA-NFAT5-mediated macrophage migration is, at least in part, dependent on CCL2 and IL-6 production. Meanwhile, either TLR2/4 antagonists (TAK-242 and OxPAPC) or genetic ablation of *Tlr2/4* completely abolished SAA-induced increases in macrophage migration and CCL2/IL6 production (Supplemental Figure 7, A–E), suggesting that such migratory process and the involvement of CCL2/IL-6 are exerted through TLR2/4.

### SAA/NFAT5 axis is essential for progression of chronic arthritis in mice.

**Figure 5:**
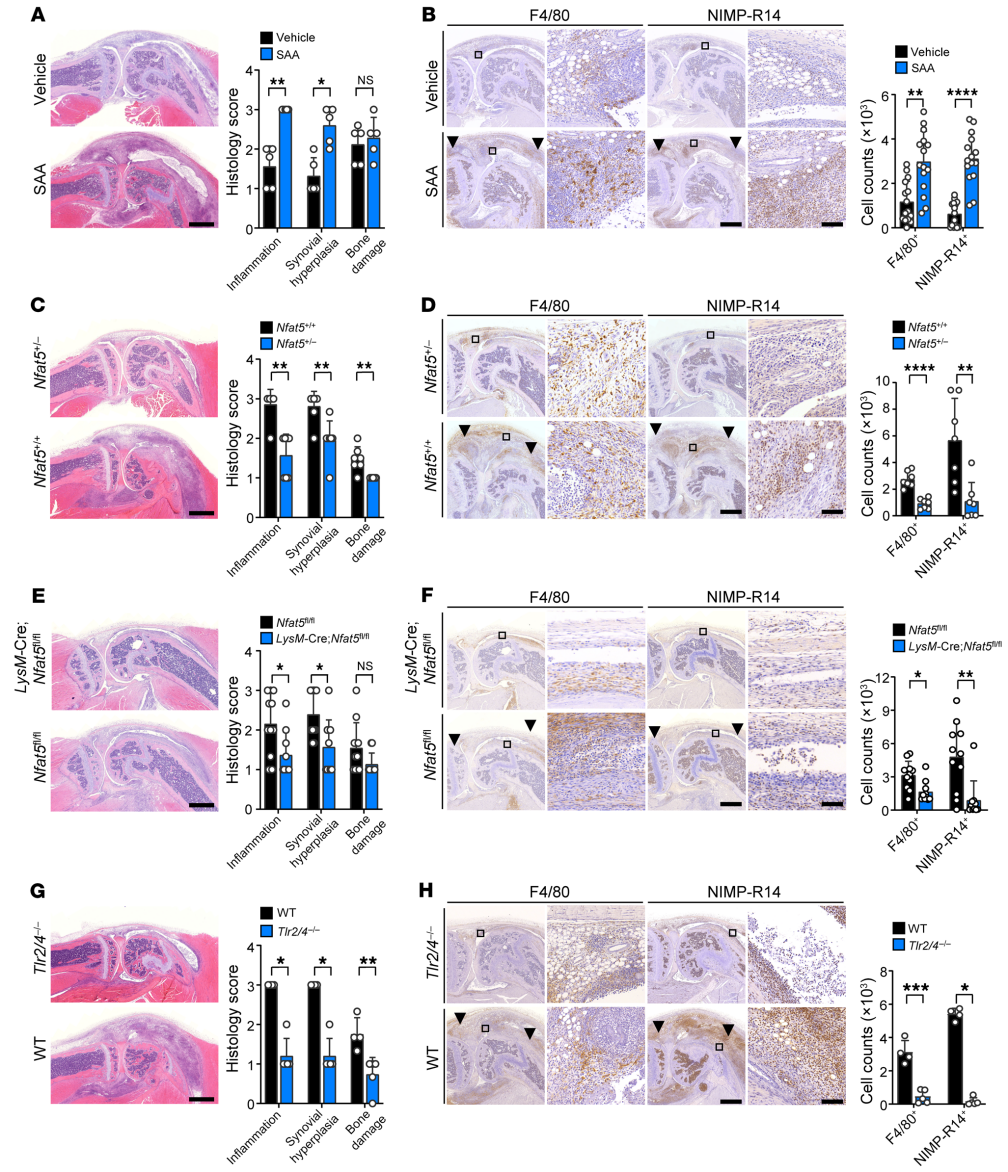
Aggravation of suboptimal form of IL-1β–induced arthritis by SAA-TLR2/4-NFAT5 axis. SAA-accelerated arthritis in mice was generated by injecting SAA (5 μg × 1) in the affected joint of mice with a suboptimal form of IL-1β–induced arthritis, which was induced by injection of mBSA (200 μg × 1) and/or IL-1β (250 ng × 2) (See Supplemental Figure 8A). Arthritis severity was scored 7 days after SAA injection on a scale of 0 to 3 depending on the extent of inflammatory cell infiltration, synovial hyperplasia, and bone destruction. (**A** and **B**) Increases in the progression of chronic arthritis and infiltration of macrophages/neutrophils by intra-articular injection of SAA. The bar graphs represent mean ± SD. *P < 0.05, **P < 0.01, and ****P < 0.0001 by Mann Whitney U test for inflammatory cell infiltration and synovial hyperplasia in (**A**), unpaired *t* test for bone destruction in (**A**) and F4/80 in (**B**), and Welch’s *t* test for NIMP-R14 in (**B**). (**C** and **D**) Amelioration of SAA-accelerated arthritis and decrement of inflammatory cell (F4/80^+^ and NIMP-R14^+^ cells) infiltration by *Nfat5* haploinsufficiency. The bar graphs represent mean ± SD. **P < 0.01 and ****P < 0.0001 by Mann Whitney U test for (**C**) and unpaired *t* test for (**D**). (**E** and **F**) Decrease in arthritis severity and inflammatory cell infiltration by specific KO of myeloid *Nfat5*. *LysM*-Cre;*Nfat5^fl/fl^* conditional KO mice were generated and subjected to SAA-accelerated arthritis. *Nfat5^fl/fl^* mice were used as controls. The bar graphs indicate mean ± SD. *P < 0.05 and **P < 0.01 by Mann Whitney U test. (**G** and **H**) Suppressive effect of *Tlr*2/4 KO on severity of SAA-accelerated arthritis and on infiltration of F4/80^+^ and NIMP-R14^+^ cells in the affected joints. The bar graphs indicate mean ± SD. *P < 0.05, **P < 0.01, and ***P < 0.001 by Mann Whitney U test for (**G**) and NIMP-R14 in (**H**), and unpaired *t* test for F4/80 in (**H**). Scale bars: 1 mm for H&E staining in **A**, **C**, **E**, and **G**. Scale bars for IHC staining of F4/80^+^ and NIMP-R14^+^ cells in **B**, **D**, **F**, and **H**: 500 μm for low magnification (left) and 50 μm for high magnification (right). Data in **A**, **C**, **E**, and **G** are representative of 2 independent experiments. Black arrowheads indicate F4/80^+^ macrophages or NIMP-R14^+^ neutrophils that have infiltrated into the synovium (**B**, **D**, **F**, and **H**).

To ascertain the pathogenic role of the SAA/NFAT5 axis in vivo, we generated a severe form of SAA-accelerated arthritis by intraarticular administration of SAA (5 μg) into the knee joint of mice with suboptimal severity of IL-1β-induced arthritis, a model of inflammatory arthritis where macrophages play a central role (33). As seen in Figure 5, A and B and Supplemental Figure 8, A and B, intraarticular injection of SAA (5 μg/mL) markedly exacerbated the severity of IL-1β–induced arthritis, as assessed by inflammatory cell infiltration (e.g., macrophage infiltration), synovial hyperplasia, and cartilage loss. However, the severity of SAA-accelerated arthritis was substantially diminished in *Nfat5*^+/–^ mice compared with their WT littermates (*Nfat5*^+/+^), suggesting that the SAA/NFAT5 axis contributes to inflammatory arthritis in vivo (Figure 5C). Moreover, the SAA-accelerated increase in infiltration of F4/80^+^ macrophages as well as NIMP-R14^+^ neutrophils was less prominent in *Nfat5*^+/–^ mice, which supports the view that SAA-NFAT5 signaling controls macrophage migration (Figure 5D). The decrease in arthritis severity in addition to macrophage infiltration was similarly reproduced in myeloid-specific *Nfat5*-deficient mice (*LysM*-Cre;*Nfat5^fl/fl^* mice) (Figure 5, E and F and Supplemental Figure 4D), demonstrating a specific proarthritic effect of myeloid NFAT5 under high SAA concentrations. Moreover, consistent with the in vitro data (Supplemental Figure 7, A–E), *Tlr2/4*-deficient mice exhibited a lesser degree of arthritis severity (Figure 5, G and H), including synovial hyperplasia, bone damage, and infiltration of F4/80^+^ macrophages.

**Figure 6:**
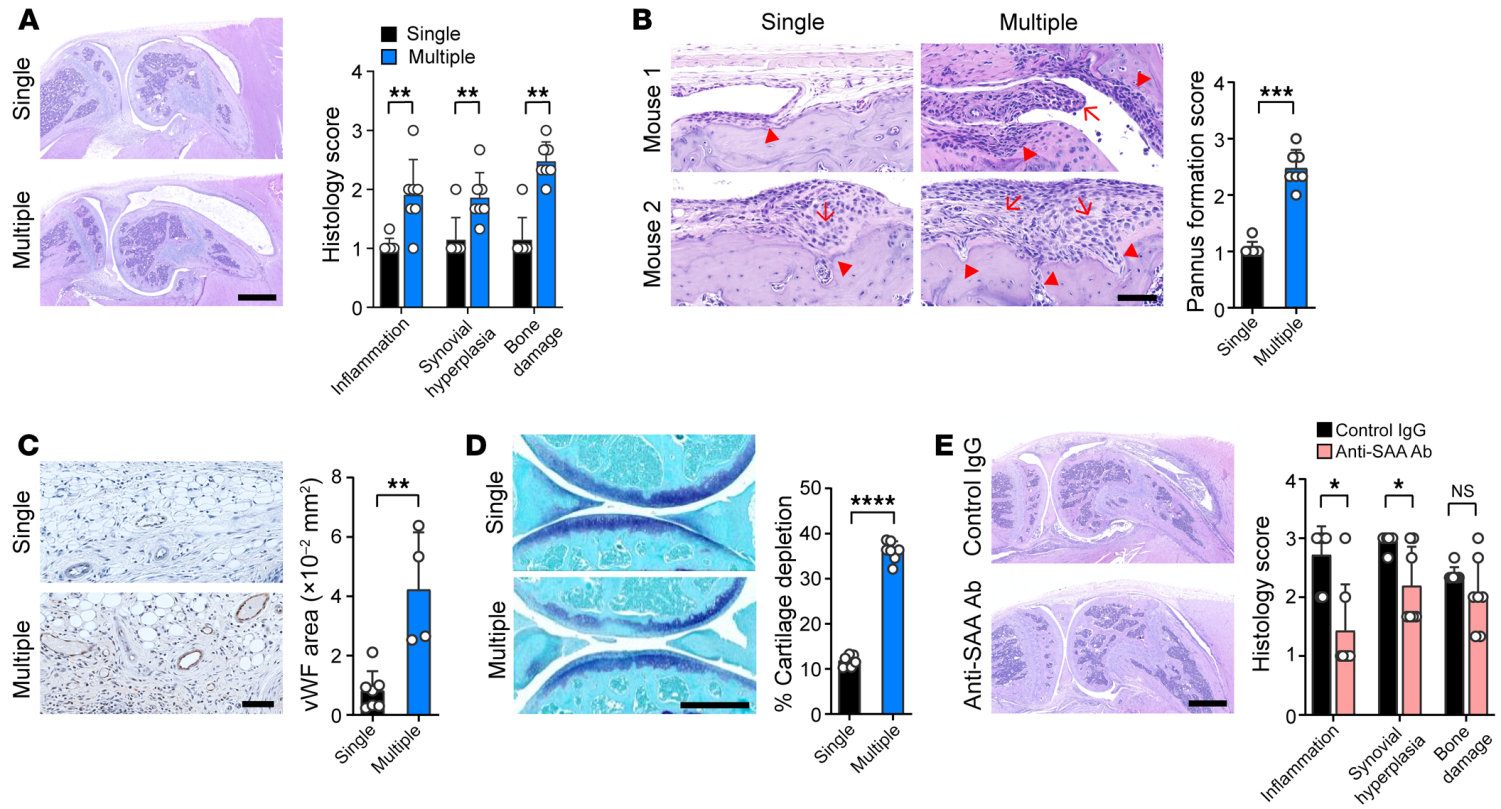
IL-1β–induced arthritis progresses to rheumatoid pathology by repeated challenges with SAA, whereas it is blocked by anti-SAA Ab. (**A**) SAA-accelerated arthritis was induced as in Supplemental Figure 8A. After 1 week, SAA (5 μg) or vehicle alone was additionally injected into the affected joint once a week for a total of 3 times. At day 28, arthritis severity was scored on a scale of 0 to 3, depending on the extent of inflammatory cell infiltration, synovial hyperplasia, and bone destruction. The bar graph represents mean ± SD. **P < 0.01 by Mann Whitney U test. (**B**) The joint pathology showing resolution of arthritis in mice singly injected with SAA versus ‘pannus formation’ (arrows) in addition to a more severe bony erosion and invasion (arrowheads) in mice receiving multiple injections of SAA. The bar graph represents mean ± SD of pannus formation score. ***P < 0.001 by Mann Whitney U test. (**C**) The degree of angiogenesis assessed by immunostaining for vWF using anti-vWF Ab. The bar graph represents mean ± SD of the stained area (mm^2^). **P < 0.01 by unpaired *t* test. (**D**) Toluidine blue stain for cartilage damage of the affected (knee) joint of mice with single versus multiple SAA injection. The bar graph indicates mean ± SD. ****P < 0.0001 by unpaired *t* test. (**E**) Amelioration of IL-1β-induced arthritis by anti-SAA Ab. Neutralizing Ab to SAA (1mg/kg) was i.p. injected on days 1 and 2 into the mice with a standard form of IL-1β–induced arthritis (IL-1β: 250 ng × 3, no SAA injection). The same concentration of isotype control IgG was used as a control. At day 7, arthritis severity was evaluated. The bar graph represents mean ± SD. *P < 0.05 and **P < 0.01 by Mann Whitney U test. Data in **A** and **E** are representative of 2 independent experiments.

Patients with RA have chronically elevated levels of serum SAA, which fluctuate over time depending on disease activity (21). We, therefore, questioned whether repeated challenge of SAA for an extended period progressively aggravates joint inflammation and destruction in mice, mimicking RA. To address this, we first generated the SAA-accelerated arthritis in mice with the same protocol as in Supplemental Figure 8A. We then repeatedly injected SAA into the affected joints once a week for an additional 3 weeks. On day 28, mice with multiple (× 4) SAA injections showed a more severe joint pathology, including inflammation, synovial hyperplasia, and bone damage, compared with those with a single SAA injection (Figure 6A). Indeed, joint inflammation and synovial hyperplasia were remarkably resolved in singly injected mice after no further SAA was challenged (Figure 6A). Notably, pannus formation (fibro-vascular hyperplasia of synovium), which is the pathologic hallmark of RA (34), as well as angiogenesis, was more frequently observed in mice with multiple injections of SAA (Figure 6, B and C). In parallel, multiple-injected mice showed a greater cartilage loss and destruction than singly injected mice (Figure 6D). Together, these observations suggest that chronic exposure to SAA may convert IL-1β–induced acute inflammation to a chronic pathology resembling RA, leading to perpetuation of inflammatory arthritis.

The results in Figures 5 and 6 clearly show that SAA directly promoted progression of arthritis in mice, but whether blocking SAA in vivo alleviates arthritis remains unknown. To address this question, we i.p. injected neutralizing Ab to SAA, which is commercially available, into the affected joints of mice with IL-1β–induced arthritis (with no SAA injection) twice, on days 1 and 2. The result showed that both joint inflammation and synovial hyperplasia were reduced by administration of anti-SAA Ab (1 mg/kg), which supports the notion that SAA directly contributes to the development of arthritis (Figure 6E) and suggests that it could potentially be a therapeutic target.

### CCL2 is required for SAA-accelerated arthritis via NFAT5.

**Figure 7:**
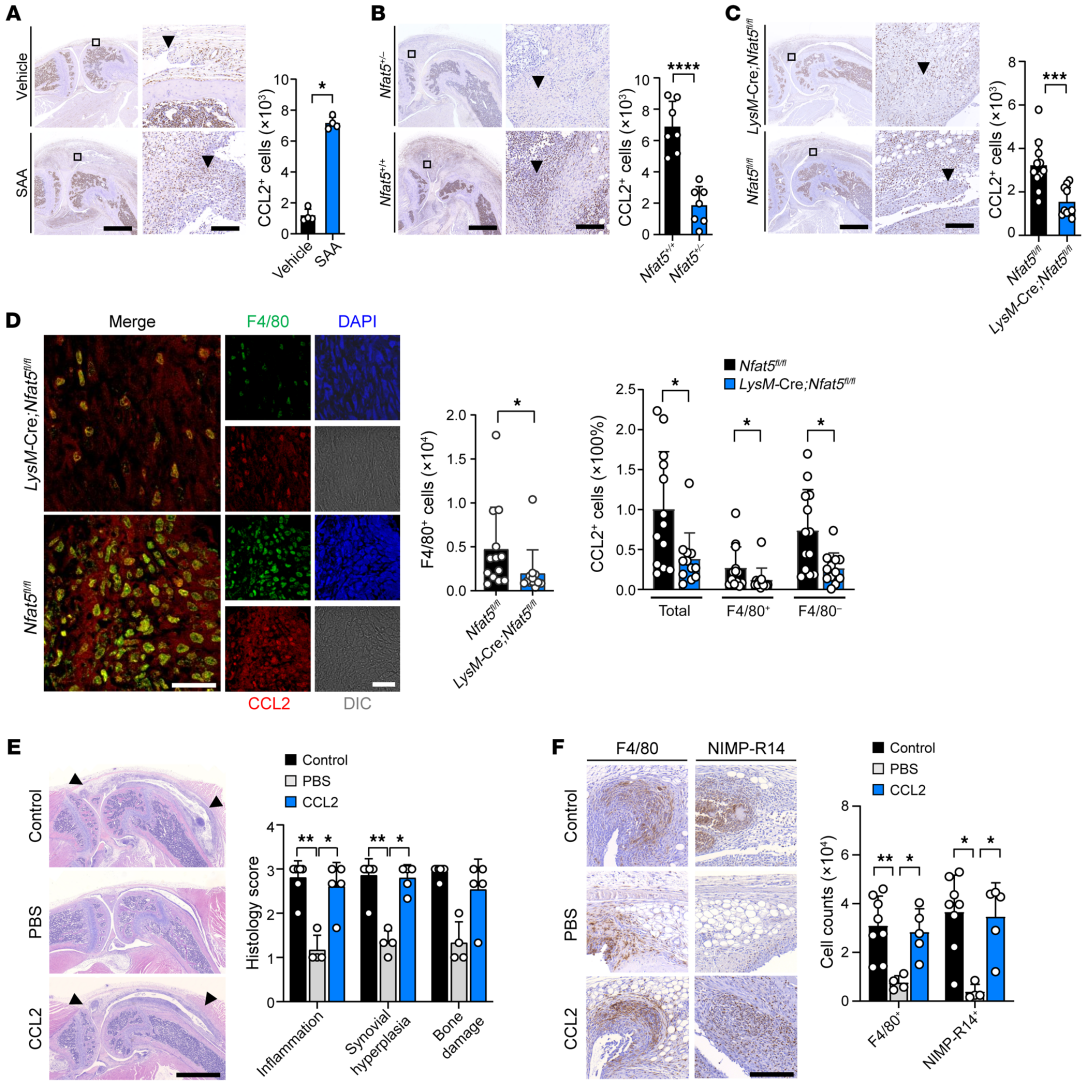
CCL2 is responsible for arthritis progression as a target of the SAA/NFAT5 axis. (**A**–**C**) Quantitation by IHC staining of CCL2 expression in the affected joints of WT (**A**), *Nfat5^+/–^* (**B**), and *LysM*-Cre;*Nfat5^fl/fl^* mice (**C**) with SAA-accelerated arthritis (See Supplemental Figure 8A), compared with their respective controls. Data represent mean ± SD; *P < 0.05, **P < 0.01, ***P < 0.001, and ****P < 0.0001 by Mann Whitney U test (**A**) and unpaired *t* test (**B** and **C**). Scale bars: 500 μm for low magnification (left panel) and 50 μm for high magnification (right panel). (**D**) Immunofluorescence colocalization for F4/80 (green) with CCL2 (red) in the affected joints of *Nfat5^fl/fl^* versus *LysM*-Cre; *Nfat5^fl/fl^* mice with SAA-accelerated arthritis. Nuclei were counterstained with DAPI (blue). The bar graphs represent mean ± SD. *P < 0.05 by Mann Whitney U test for immunostaining of F4/80^+^ cells and Kruskal-Wallis test with post hoc Mann-Whitney U test for immunostaining of CCL2^+^ cells. Scale bars: 20 μm. (**E**) Histological severity of the affected joints of *LysM*-Cre;*Nfat5^fl/fl^* mice injected intraarticularly with CCL2 (2 μg × 3) versus vehicle alone. The *Nfat5*^fl/fl^ mice with SAA-accelerated arthritis were used as a positive control. Mean histological severity is shown on the right. Data are mean ± SD. *P < 0.05 and **P < 0.01 by Kruskal-Wallis test with a Dunn’s multiple comparisons test. Scale bars: 500 μm. Data are representative of 2 independent experiments. (**F**) Quantitation of F4/80^+^ macrophage and NIMP-R14^+^ neutrophil infiltration in the affected joints between *LysM*-Cre;*Nfat5^fl/fl^* mice injected intraarticularly with CCL2 (2 μg × 3) and those with vehicle alone. The bar graph indicates mean ± SD. *P < 0.05 and **P < 0.01 by 1-way ANOVA with Tukey’s multiple comparisons test for immunostaining of F4/80 and Kruskal-Wallis test with a Dunn’s multiple comparisons test for immunostaining of NIMP-R14. Scale bars: 50 μm.

We demonstrated that macrophage migration by SAA-NFAT5 signaling is dependent on CCL2 and IL-6 production in vitro, and such dependency was more prominent with CCL2 than with IL-6 (Figure 4, D and E). Based on these findings, we examined CCL2 expression in the SAA-accelerated arthritis model. As expected, intraarticular injection of SAA caused a marked increase in CCL2 expression in the affected joints (Figure 7A). In contrast, when SAA was injected into *Nfat5*^+/–^ and *LysM*-Cre;*Nfat5^fl/fl^* mice, the number of CCL2-expressing cells as well as F4/80^+^ cells was significantly lower than in their respective WT littermates (*Nfat5*^+/+^ and *Nfat5^fl/fl^*) (Figure 7, B–D). Moreover, CCL2 expression in F4/80^+^ cells was substantially downregulated in synovium of *LysM*-Cre;*Nfat5^fl/fl^* mice compared with *Nfat5^fl/fl^* mice, indicating that NFAT5 contributes to the induction of CCL2 expression in macrophages (Figure 7D). In parallel, CCL2 expression in NIMP-R14^+^ cells, but not in nonmyeloid CD90^+^ cells, was also remarkably reduced in the same *LysM*-Cre;*Nfat5^fl/fl^* mice (Figure 7D and Supplemental Figure 9), which may explain the decrease in CCL2 expression in F4/80^–^ cells in Figure 7D.

*Tlr2/4*-deficiency also resulted in a decrease in CCL2 expression in the synovium of mice injected with SAA (Supplemental Figure 7F). Conversely, intraarticular injection of CCL2 into the affected joints almost completely restored the severity of SAA-accelerated arthritis in *LysM*-Cre;*Nfat5^fl/fl^* mice compared with *Nfat5^fl/fl^* mice (Figure 7E). Injection of IL-6 into the affected joints also almost completely reversed arthritis reduction by NFAT5 deficiency (Supplemental Figure 10), suggesting that IL-6, in addition to CCL2, mediates the progression of SAA/NFAT5 axis–dependent arthritis. Macrophage and neutrophil infiltration were also in concordance with this phenotype, such that the number of F4/80^+^ or NIMP-R14^+^ cells was remarkably recovered by the administration of CCL2 (Figure 7F). To summarize, it was demonstrated that the SAA/NFAT5 axis promoted chronic arthritis and macrophage infiltration in affected joints, presumably by increasing production of CCL2 and IL-6, the NFAT5 targets.

### SAA1 overexpression in the liver accelerates progression of mBSA/IL-1β–induced arthritis.

**Figure 8:**
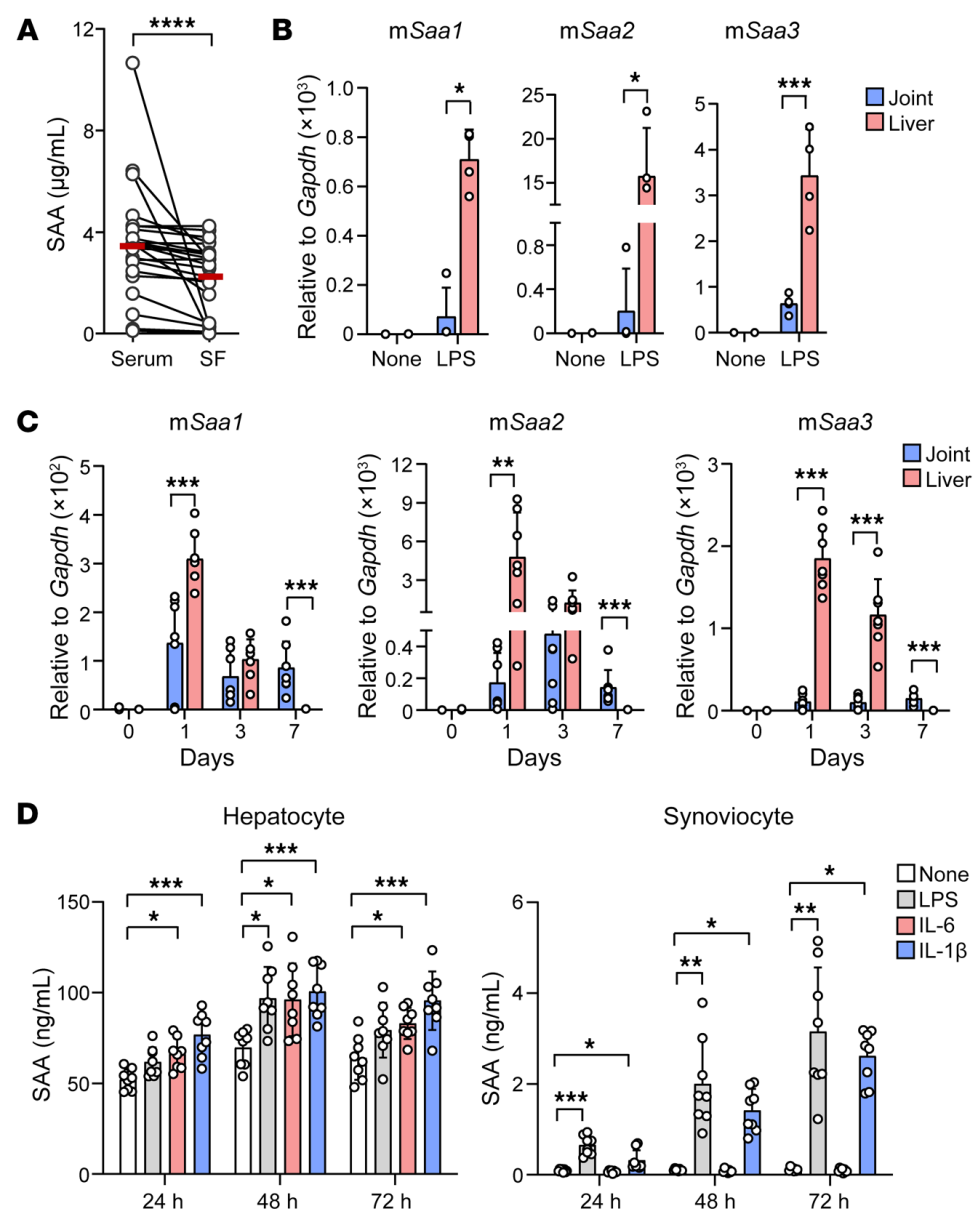
Liver is one of the major sources of SAA production in inflammatory arthritis. (**A**) Levels of SAA in paired sera and synovial fluids isolated simultaneously from patients with RA (*n* = 25), as determined by ELISA. Data are mean ± SD. ****P < 0.0001 by Wilcoxon matched pairs signed rank test. (**B** and **C**) qPCR analysis of m*Saa1*, m*Saa2*, and m*Saa3* mRNA expression levels in the liver and joint tissues of mice injected i.p. with 10 mg/kg of LPS (**B**) or in those of mice injected with mBSA (200 μg, 1 × on day 0) and/or IL-1β (250 ng, 3 × on days 0, 1, and 2) (**C**). Data are mean ± SD. *P < 0.05, **P < 0.01, and ***P < 0.001by Kruskal-Wallis test for m*Saa1* and m*Saa2* (**B**), Brown–Forsythe and Welch’s ANOVA test for m*Saa3* (**B**), 2-way ANOVA test for m*Saa1* and m*Saa3* (**C**), and Friedman’s test for m*Saa2* (**C**). Comparison of numerical data between groups were performed using the unpaired *t* test, Welch’s *t* test, or Mann-Whitney U test. Data are representative of 2 independent experiments. (**D**) SAA production from cultured hepatocytes and synoviocytes. Hepatocytes (1 × 10^5^/well) and fibroblast-like synoviocytes (5 × 10^4^/well), isolated from C57BL/6 mice, were stimulated with LPS (10 ng/mL), IL-6 (10 ng/mL), and IL-1β (10 ng/mL) for the indicated times. The concentration of SAA in the culture supernatants was determined by ELISA. Data are mean ± SD. *P < 0.05, **P < 0.01, ***P < 0.001, and ****P < 0.0001 by 1-way ANOVA with Tukey’s multiple comparisons test.

Although SAA is predominantly produced by the liver, there is ample evidence that it is also highly expressed in arthritic joints (21, 35). To determine the relative contribution of the 2 organs to SAA generation under inflammatory conditions, we measured SAA levels in the sera and synovial fluids obtained simultaneously from patients with RA (*n* = 25). SAA concentrations were found to be significantly higher in the sera than in the synovial fluids of patients with RA (mean levels: 3.6 ± 2.1 and 2.3 ± 1.4 μg/mL, *P* < 0.01, Figure 8A), suggesting that chronic arthritis stimulates production of SAA from extraarticular sources such as the liver, thus contributing to the higher level of SAA in sera.

To validate this assumption, we induced a local form of arthritis in mice and then measured SAA expression in the liver and joints simultaneously. As a control experiment, we confirmed that i.p. injection of LPS instigated expression of m*Saa1*, m*Saa2*, and m*Saa3*, which was much higher in the liver than in the joints (Figure 8B). Interestingly, when a local form of arthritis was induced by injection of methylated bovine serum albumin (mBSA) plus IL-1β (× 3) into mouse joints, expression levels of all 3 isoforms of mSAA were also higher in the liver than in the joints at an earlier stage of arthritis (days 1 and 3) while becoming much lower in the liver at later stages (day 7) (Figure 8C). In support of these findings, SAA production from cultured hepatocytes was increased by stimulation with LPS, IL-6, and IL-1β. SAA secretion from synovial fibroblasts also was instigated by LPS and IL-1β, but its levels were much lower than those from hepatocytes (mean ± SD: 0.08 ± 0.03 to 3.15 ± 1.32 ng/mL for synoviocytes versus 51.9 ± 5.5 to 101.7 ± 13.5 ng/mL for hepatocytes) (Figure 8D). Interestingly, IL-6 did not affect SAA production by synovial fibroblasts, which suggests that different stimuli are required for synovial fibroblasts and hepatocytes to generate SAA.

**Figure 9:**
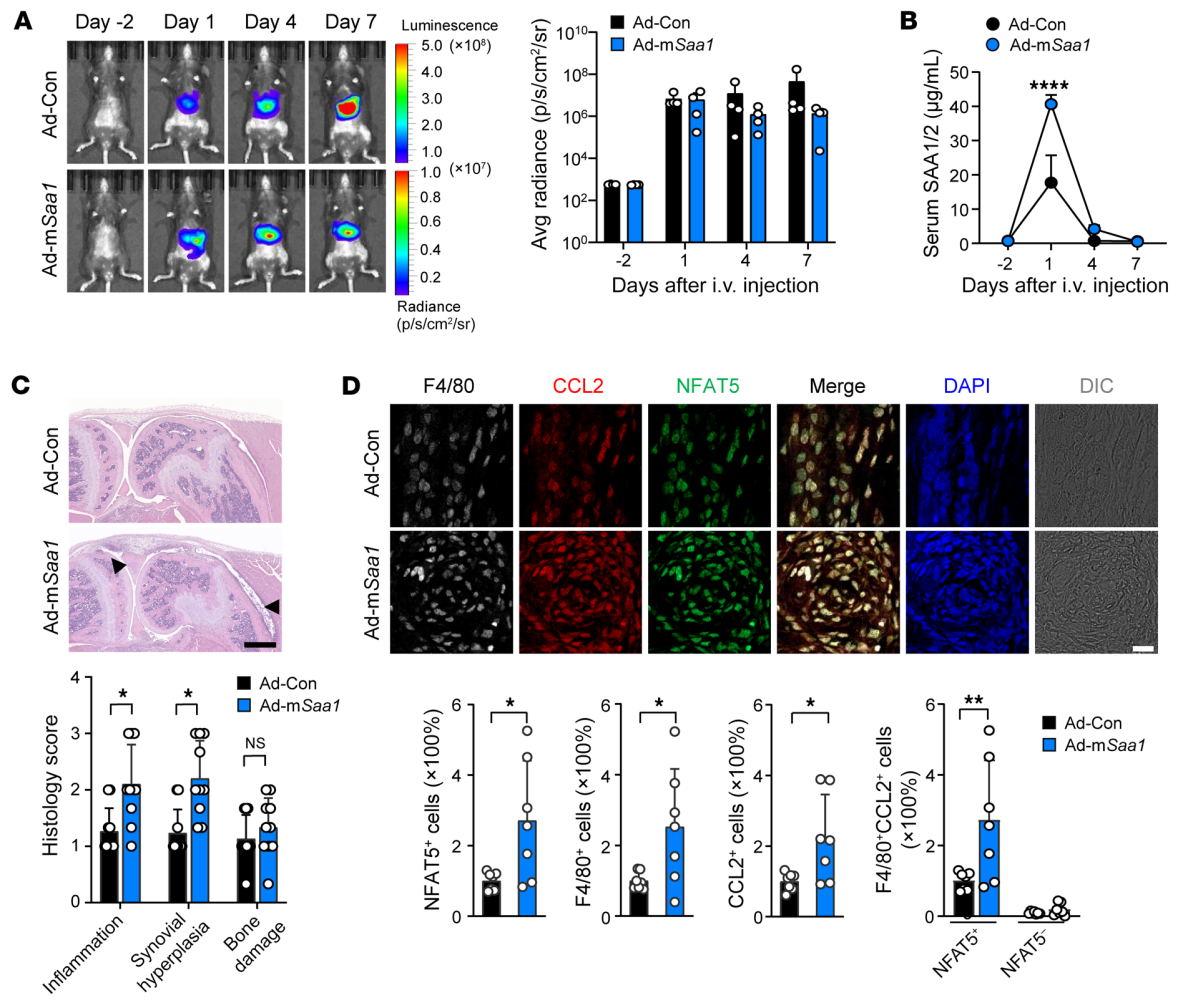
Accelerated chronic arthritis by forced overexpression of SAA in the liver. (**A**) In vivo luciferase imaging of mouse whole body with a suboptimal form of IL-1β-induced arthritis before and after i.v. injection of Ad-m*Saa1* and Ad-Con (See Supplemental Figure 8C). Mean (± SD) bioluminescence signal intensity (p/s/cm^2^/sr) in the liver at different time points is shown on the right. (**B**) SAA1/2 levels in the sera of mice with IL-1β–induced arthritis before and after injecting Ad-m*Saa1* versus Ad-Con, as determined by ELISA. Data are mean ± SD. ****P < 0.0001 by 2-way ANOVA with Šidák’s multiple comparisons test. (**C**) Deterioration of chronic arthritis by overexpression of *Saa1* in the livers of mice. 7 days after injection of Ad-m*Saa1* and Ad-Con, arthritis severity was histologically assessed. The bar graph represents mean ± SD. **P < 0.01 by unpaired *t* test. Scale bars: 500 μm (**D**) Quantitation of F4/80^+^ macrophage infiltration and immunofluorescence colocalization for CCL2 (red), NFAT5 (green), and F4/80 (white) in the affected joints of mice with forced overexpression of *Saa1* in the liver compared with control mice without *Saa1* overexpression. The bar graphs indicate mean ± SD. *P < 0.05 and **P < 0.01 by Welch’s *t* test for NFAT5^+^, F4/80^+^, and CCL2^+^ cells, and by 1-way ANOVA with Tukey’s multiple comparisons test for F4/80^+^ CCL2^+^ (double positive) cells. Scale bars: 20 μm. Data are representative of 2 independent experiments.

The finding of elevated SAA in the liver by inflammatory arthritis, specifically by IL-6 and IL-1β, raised the intriguing hypothesis that with the presence of arthritis, the liver takes an active part in exacerbating disease progression by secreting SAA. To address this, we sought to overexpress m*Saa1* gene in the liver by adenovirus-mediated transfer of luciferase-tagged m*Saa1* (Ad-m*Saa1*) to just the liver (Supplemental Figure 8C). Of the 4 isoforms, m*Saa1* was selected because it is known to be the most pathogenic (36, 37). We first confirmed that after i.v. injection of Ad-m*Saa1*, a bioluminescence signal reflecting luciferase activity was selectively increased in the liver (Supplemental Figure 11A and 11B), which corresponded to high concentrations of mSAA1/2 in the sera (Supplemental Figure 11C). No bioluminescence was detected in the joints (Supplemental Figure 11, A and B). In mice with mBSA+IL-1β–induced arthritis, the signal was also increased exclusively in the liver after viral transfer similarly in mice with Ad-m*Saa1* versus those with empty vector only (Figure 9A). Meanwhile, serum SAA1/2 concentrations were significantly higher in mice with Ad-m*Saa1* than those with control vector at the preclinical stage of disease (Figure 9B). Most strikingly, on day 7, hepatic (extraarticular) overexpression of m*Saa1* caused an increase in the pathology of the affected joints, i.e., at remote sites, in mice with IL-1β–induced arthritis (Figure 9C). In parallel, the number of NFAT5^+^ or CCL2^+^ cells and F4/80^+^ macrophages were more frequently detected in mice with Ad-m*Saa1* (Figure 9D). Importantly, hepatic overexpression of m*Saa1* remarkably increased CCL2 expression in NFAT5^+^ macrophages in the arthritic joints, but not in NFAT5^–^ macrophages (Figure 9D), supporting the view that hepatic SAA1 activates the NFAT5/CCL2 axis in joints. In sum, these data argue that liver has a proinflammatory role through secretion of SAA, and potentiates the TLR2/NFAT5/CCL2 pathway in macrophages infiltrated in arthritic joints.

### Serum SAA levels reflect RA activity and treatment response to antirheumatic drugs.

**Figure 10:**
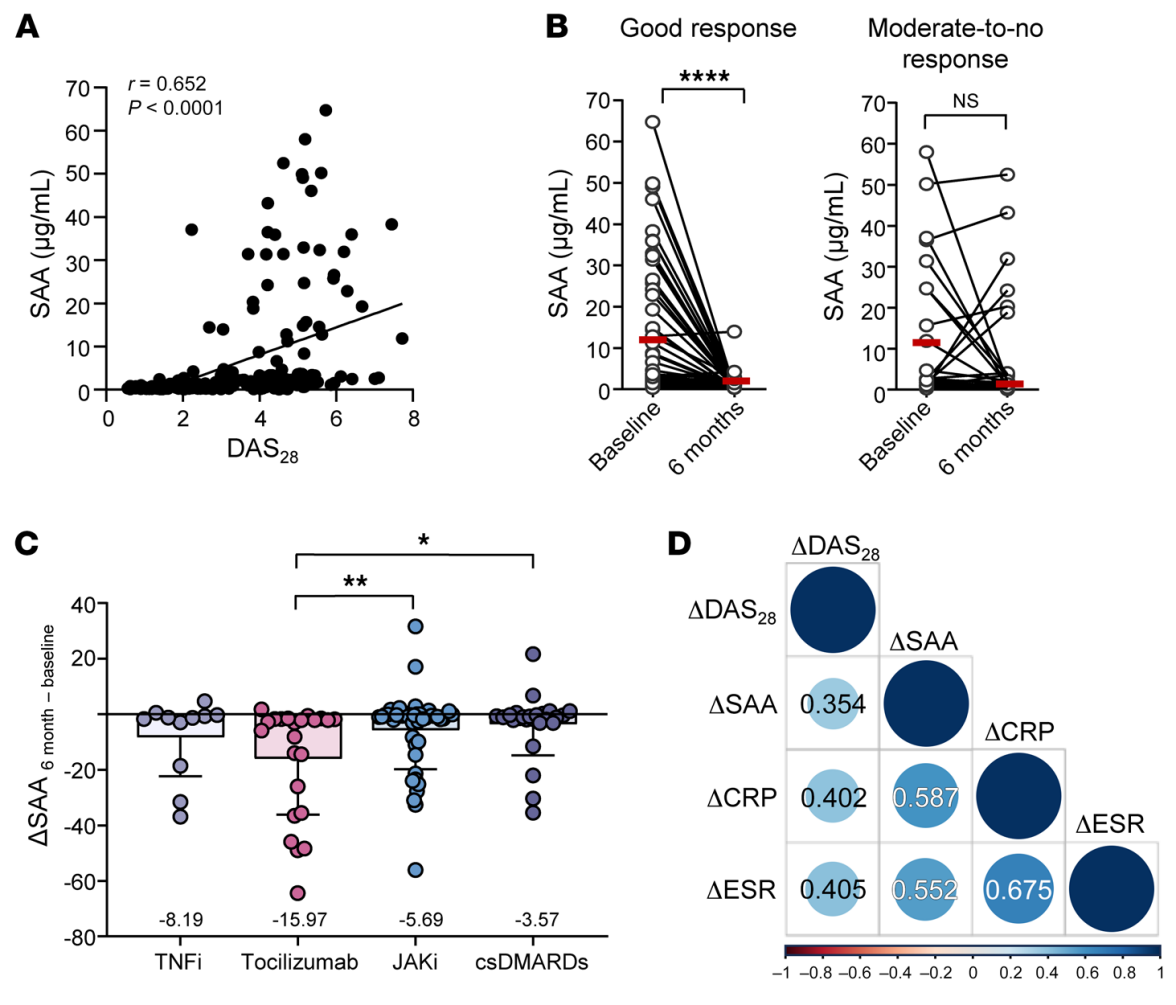
Serial monitoring of serum SAA levels before and after treatment with antirheumatic drugs in patients with RA. (**A**) Correlation between SAA level and disease activity of RA. Serum SAA levels were measured by ELISA both before and 6 months after treatment with antirheumatic drugs, including TNFi, Tocilizumab, an anti-IL-6 receptor Ab, JAKi, and conventional synthetic disease-modifying antirheumatic drugs (csDMARDs) (See Supplemental Table 1 for more details). At the time of blood sampling, the DAS_28_, which represents RA activity, was assessed. The correlation analysis was performed using the Spearman test. (**B**) The ΔSAA from baseline to 6-month follow-up according to the treatment response. Good, moderate, and no response were defined according to EULAR response criteria. ****P < 0.0001 by Wilcoxon matched pairs signed rank test. (**C**) Comparison of ΔSAA levels depending on the kinds of antirheumatic drugs, including TNFi, Tocilizumab, JAKi, and csDMARDs. Data are mean ± SD. *P < 0.05 and **P < 0.01, by Kruskal-Wallis test with Dunn’s multiple comparisons test. (**D**) Positive correlations of ΔSAA with ΔCRP, ΔESR, and ΔDAS_28._ The correlation analysis was performed using the Spearman test.

Finally, we wondered how the SAA/NFAT5 axis can be therapeutically targeted, especially in the context of using antirheumatic drugs, such as IL-6 inhibitor and Janus kinase inhibitor (JAKi), which suppress the production of acute phase reactants like SAA. To address this question, we sought to determine dynamic changes of SAA concentrations in the sera of 103 patients with active RA (DAS28 > 3.2) at the start of each treatment and after 6 months. Among these patients, 24% were treated with conventional disease-modifying antirheumatic drugs (csDMARDs), 11% with a TNF-α inhibitor (TNFi), 22% with Tocilizumab, the antiinterleukin-6 receptor (IL-6R) Ab, and 43% with JAKi (Supplemental Table 1). As reported previously (21), serum SAA level correlated well with the Disease Activity Score with 28 joint counts (DAS_28_) (r = 0.652, *P* < 0.001, Figure 10A), which is one of the standard methods to measure RA activity (38). Moreover, when serially monitored, serum SAA levels significantly decreased from baseline to 6 months in the good response group (*n* = 75, 73%), whereas no significant change was found in the moderate-to-no response group (*n* = 28, 27%) (Figure 10B), suggesting that serum SAA concentration represents treatment response to antirheumatic drugs. IL-6 is a well-known cytokine that induces SAA production by the liver (39). Interestingly, the change of SAA level (ΔSAA) from baseline to 6 months was significantly higher in patients with RA who were treated with Tocilizumab than in those treated with JAKi and csDMARDs (Figure 10C and Supplemental Figure 12). The ΔSAA values correlated well with the ΔCRP, ΔESR, and ΔDAS_28_ (*r* = 0.587 and *P* < 0.001, *r* = 0.552 and *P* < 0.001, and *r* = 0.354 and *P* < 0.001, respectively) (Figure 10D). Taken together, this prospective clinical study of patients with RA demonstrated that serum SAA levels can be changed by antirheumatic drugs, most strikingly by Tocilizumab, an inhibitor of SAA-stimulating cytokine IL-6, presumably reflecting RA activity and therapeutic outcome.

## Discussion

Activation of NFAT5, a well-known osmoprotective factor, can be induced by isotonic stimuli, such as infection. It was unclear, however, whether NFAT5 mediated DAMP-triggered inflammation and immunity. In the present study, we found that, similar to PAMPs, several DAMPs, including SAA, MRP8, and HMGB1, can induce NFAT5 expression. In particular, acute phase reactant SAA substantially increased NFAT5 expression and activity in RAW 264.7 and primary macrophages. SAA-mediated NFAT5 upregulation was operative via TLR2/4-JNK signaling pathway. Moreover, triggering of the SAA-NFAT5 axis provoked migration and chemotaxis of macrophages and deteriorated chronic arthritis in CCL2- and IL-6–dependent manner. Conversely, myeloid-specific depletion of NFAT5 attenuated SAA-accelerated arthritis. Notably, overexpression of *Saa1* in the liver substantially increased IL-1β–induced arthritis. Taken together, this study demonstrates that SAA-TLR2/4-NFAT5 signaling is crucial for macrophage migration and chronic arthritis, suggesting the proinflammatory role of the liver in the propagation of chronic inflammation at remote sites.

One way that a given molecule acquires multifunctionality would be associating it with a distinct receptor and signaling pathway. SAA seems to display its diverse immune-modulating activities by utilizing different receptors and signaling pathways depending on cell type and function. For instance, SAA induces robust expression of cytokines and chemokines in macrophages and endothelial cells, which is mediated by the TLR2/4-NF-ĸB axis (14, 40). SAA also activates ERK1/2 and p38 MAPK, promoting IL-8 production by THP1 monocytes through CLA1 (CD36 and LIMPII analogous-1) (41). On the other hand, in synovial fibroblasts, SAA interacts with its receptor FPRL1 to enhance cell proliferation via the ERK and AKT pathways (17). Here, we identified a branch of a SAA-TLR2/4-JNK1/2-NFAT5 signaling pathway utilized in macrophages. Specifically, our report is the first to demonstrate the pivotal role of NFAT5 in migration and chemotaxis of macrophages exposed to DAMPs, including SAA and MRP8. We presume that inhibition of this axis may hold promise in the development of antimacrophage therapies in chronic inflammatory diseases characterized by elevated SAA and permit selective targeting of potentially harmful macrophages while sparing beneficial cells.

Macrophages have a central role in initiation and perpetuation of chronic inflammatory arthritis, including RA (20, 42). In this study, intraarticular injection of SAA markedly propelled progression of IL-1β–induced arthritis. By contrast, *Nfat5*-haplodeficient and *Tlr2/4*-deficient mice all exhibited a milder form of SAA-accelerated arthritis, including synovial hyperplasia and bone damage, which was accompanied by diminished macrophage infiltration, thereby confirming the critical role of SAA-TLR2/4-NFAT5 axis in macrophage migration and chronic arthritis in vivo. Such reduction was similarly noted in *LysM*-Cre;*Nfat5^fl/fl^* mice, thus demonstrating an indispensable effect of myeloid NFAT5 on the development of chronic arthritis (43). Moreover, as in mice, patients with RA had much higher levels of SAA in their synovial fluids and elevated NFAT5 expression in synovial macrophages than did patients with noninflammatory OA. Importantly, macrophage NFAT5 levels correlated well with SAA concentrations in RA synovial fluids. In summary, these data, together along with earlier reports (20, 42), indicate that the SAA-TLR2/4-NFAT5 axis in macrophages is necessary for the progression of chronic arthritis.

CCL2 is a classical inflammatory chemokine expressed by a variety of cell types, including macrophages. In this study, CCL2 was a major downstream target of the SAA-TLR2/4-NFAT5 axis to augment migration and chemotaxis of macrophages in vitro. Moreover, in mice with SAA-accelerated arthritis, myeloid-specific depletion of *Nfat5* mitigated infiltration of CCL2-expressing cells in the affected joints, particularly in infiltrating macrophages. Recombinant CCL2 almost completely restored macrophage infiltration dampened by *Nfat5*- or *Tlr2/4–*deficiency. Therefore, the myeloid NFAT5-CCL2 pathway seems to be critically involved in SAA-induced macrophage infiltration into the arthritic joints. Notwithstanding, CCL2 can be produced by nonmyeloid cells, including synovial fibroblasts, via the SAA/NFAT5 axis, as shown in Figure 7D and Supplemental Figure 9. Moreover, rescue experiments using recombinant IL-6 indicated that the SAA/NFAT5–induced increase in macrophage migration and arthritis progression also is dependent on IL-6. Since NFAT5 regulates a number of proinflammatory genes and biologic processes, such as macrophage survival (20), it is unlikely that myeloid NFAT5/CCL2 axis alone entirely mediates the SAA promotion of macrophage migration and chronic arthritis. Multiple genes and other extramigratory cellular functions governed by NFAT5 may cooperatively contribute to this process.

Evidence is emerging that acute phase reactants, for which the liver is a major source, have direct immune-modulating activities (44, 45). However, dynamic, mutual interaction between liver and joint in chronic inflammatory diseases is poorly understood. Here, we identified that *Saa1*, *Saa2*, and *Saa3* expression in the liver, an extraarticular organ, were markedly increased in IL-1β–induced arthritis. Moreover, hepatocytes produced a much higher amount of SAA when stimulated with LPS, IL-1β, and IL-6 than synoviocytes, suggesting that TLR4 ligands and the cytokines secreted from inflamed joints to the periphery could trigger SAA production by the liver. Conversely, overexpression of *Saa1* in the liver deteriorated arthritis progression, accompanied by increased macrophage infiltration and CCL2 expression in the affected joints, (extrahepatic organs), demonstrating that the liver contributes to chronic arthritis as an active participant in the immune system. In such case, a more severe form of arthritis exacerbated by liver-derived SAA might further activate liver to secrete more SAA, constructing a feed-forward cycle connecting the liver and joints (Supplemental Figure 13). However, given that the elevated serum SAA in nonarthritic inflammatory conditions is not commonly accompanied by arthritis, it is unlikely that SAA alone can initiate joint inflammation. Rather, the SAA/NFAT5 axis may require an initial priming of synovitis repeatedly secreting a high amount of IL-1β and/or IL-6 so as to establish a chronic form of arthritis.

It remains unclear whether SAA could be a therapeutic target in patients with RA. Here, the severity of IL-1β-induced arthritis was dampened by anti-SAA Ab treatment, which suggests that SAA can be targeted to retard IL-1β–dependent arthritides, including gouty arthritis and collagen-induced arthritis (46, 47). Conversely, repeated injection of SAA into the affected joints accelerated arthritis progression, closely mimicking RA pathology, indicating that SAA not only exacerbates acute inflammatory arthritis but also propels it to chronic form, presumably by bypassing the spontaneous resolution seen in single SAA injection. Moreover, serum SAA concentration was remarkably reduced by antirheumatic drugs in patients with RA, particularly by Tocilizumab, an inhibitor of potent SAA-inducing cytokine IL-6, correlating well with RA activity and treatment outcomes. Taken together, our work underscores the importance of SAA in the perpetuation of chronic inflammatory arthritis, suggesting it as a new therapeutic target. If this is the case, then inhibition of the SAA/NFAT5 axis, for example, via shRNA or small molecule intervention, would repress the interorgan communication and represent therapeutic strategies for chronic inflammatory diseases characterized by excessive production of SAA, including encephalomyelitis, inflammatory bowel disease, and RA (48, 49).

This study has some limitations. First, we observed SAA-induced exacerbation of IL-1β–induced arthritis over a relatively short 7-day period. Consequently, it is imperative to validate our primary findings in a long-term chronic arthritis model, such as collagen-induced arthritis. Second, focusing solely on the SAA/NFAT5/CCL2 axis could overlook other crucial pathways in the pathogenesis of RA. RA involves a complex network of cytokines, chemokines, and cellular interactions (50, 51). Therefore, a more extensive investigation into how NFAT5 deficiency affects various aspects of immune regulation could enrich our understanding of its role in chronic inflammatory arthritis. Third, relying heavily on animal models and in vitro experiments, without substantial clinical data from humans, might restrict the generalizability of our findings to human RA. Given RA’s heterogeneity, incorporating direct clinical data from patients with RA would strengthen the evidence for the roles of SAA, NFAT5, and CCL2 in RA progression.

In summary, we have identified a signaling pathway of TLR2/4-NFAT5 activated in macrophages upon exposure to DAMPs, including SAA and MRP8. Specifically, the SAA-TLR2/4-NFAT5 axis critically mediates migration/chemotaxis of macrophages via CCL2, instigating infiltration of macrophages in the affected joints and accelerating progression of chronic arthritis. Most strikingly, inflammatory arthritis upregulates SAA expression in the liver, which, in turn, exacerbates pathology within the joints. We anticipate that our data provides a pathophysiologic basis that reveals insights into interorgan communication responsible for macrophage activation and chronic arthritis, including RA. These findings may facilitate therapeutic intervention to ameliorate a diverse array of DAMPs-associated chronic inflammatory diseases.

## Methods

### Sex as a biological variable.

Our study examined male and female humans and animals, and similar findings are reported for both sexes.

### Preparation of BMDMs, peritoneal macrophages, CD14^+^ cells, hepatocytes, and synoviocytes.

BMDMs were generated by differentiating bone marrow progenitors from the tibia and femur of each mouse (C57BL/6J WT, *Tlr2*^–/–^, *Tlr4*^–/–^, and *Tlr2/4*^–/–^ mice) as described previously (52). Bone marrow cells were cultured in IMDM (Gibco) supplemented with 10% FBS, 30% L-cell (ATCC) supernatant, nonessential amino acids (Gibco), sodium pyruvate (Gibco), and antibiotics (penicillin/streptomycin; Gibco). L-cell supernatant was collected after 7 days of culture in IMDM supplemented with 10% FBS. The culture medium was replaced with fresh medium on day 3. On day 7, adherent cells were collected for experiments. For preparation of peritoneal macrophages, each mouse was i.p. injected with 3 mL of 3% sterile thioglycollate solution (BD Difco) and, 3 days later, macrophages were isolated from the peritoneal lavage fluid as described previously (53). CD14^+^ cells were obtained from PBMCs of healthy individuals using anti-CD14 magnetic beads (Miltenyi Biotec) according to the manufacturer’s instructions. Hepatocytes were isolated from C57BL/6 mice using a liver dissociation kit (Miltenyi Biotec). Mouse fibroblast-like synoviocytes (FLSs) were isolated from the synovial tissues of C57BL/6 mice as previously described (54).

### Patients and the healthy control group.

In total, 103 patients who met the 2010 RA classification criteria of the American College of Rheumatology (ACR)/European League Against Rheumatism (EULAR) were studied (55) (Supplemental Table 1). The mean age of the patients with RA was 53.9 ± 12.1 years, and women accounted for 89.3% while men accounted for 10.7%. The mean disease duration was 5.5 ± 6.2 years. Among the 103 patients with RA, 77/98 (79%) were positive for rheumatoid factor and 51/61 (84%) were positive for the anti-cyclic citrullinated Abs. All these patients with moderate-to-high disease activity (disease activity score in 28 joints [DAS_28_] > 3.2) at baseline. The mean DAS_28_ was 4.73 ± 1.06. These patients initiated conventional synthetic disease-modifying antirheumatic drugs (csDMARDs, 24.3%), TNFi (10.7%), anti-IL-6 receptor agent (tocilizumab, 23.3%) or JAKi (42,7%). The treatment response for 6 months was determined by the EULAR response criteria based on DAS_28_ (38) and it was categorized by a good response group and a moderate-to-no response group. Comparisons were made with 96 people in a healthy control group (16 men and 80 women) who had no rheumatic diseases. The mean age of the people in the healthy control group was 56.8 ± 10.2 years old. No difference was found in age and sex between patients with RA and people in the healthy control group. All patients and people in the healthy control group gave written informed consent to the study protocol. Their serum, synovial fluid, and synovium were used in this study.

### Mice.

WT C57BL/6 and BALB/c mice were purchased from Orientbio. *Nfat5*^+/–^ and *LysM*-Cre;*Nfat5^fl/fl^* mice on a C57BL/6 background (provided by H. Moo Kwon, Ulsan National Institute of Science and Technology, Ulsan, Republic of Korea) were mated with WT C57BL/6 (*Nfat5*^+/+^) and *Nfat5^fl/fl^* mice, respectively, and were held under specific pathogen free (SPF) conditions in the animal facilities at the Catholic University of Korea. *Tlr2*^–/–^, *Tlr4*^–/–^, and *Tlr2/4*^–/–^ mice on a C57BL/6 background were provided by Jong-Hwan Park (Chonnam National University, Gwangju, Republic of Korea) and they were maintained under standard conditions in the animal facilities at the Catholic University of Korea. Mice were randomly allocated into experimental groups.

### Statistics.

Statistical analyses were conducted using GraphPad Prism v.8.4.3. The normality of the data distribution was verified using the D’Agostino and Pearson test, Shapiro-Wilks test, or Kolmogorov-Smirnov test and for homogeneity of variances was tested using F test or Brown–Forsythe test. Differences between 2 groups were analyzed by Wilcoxon matched pairs signed rank test, 2-tailed, unpaired *t* test, Welch’s *t* test, or Mann Whitney U test. Comparisons among multiple groups were performed by 1-way ANOVA with Tukey’s multiple comparisons, Brown-Forsythe and Welch ANOVA with Dunnett T3 multiple-comparison test, or Kruskal-Wallis test with Dunn’s multiple comparisons test. In the case of time-course data, 2-way ANOVA with Šidák’s multiple comparisons test or Friedman’s test with Dunn’s multiple comparisons test was used to determine significance. The correlation analysis was performed using the Pearson test or Spearman test. Data are shown as mean ± SD and *P* values lower than 0.05 were considered significant.

### Study approval.

Human study was performed with the approval of the IRB (nos. CUMC09U034 and KC16SISI0632) at Seoul St. Mary’s Hospital of the Catholic University of Korea. All animal experiments were performed in accordance with protocols approved by the IACUC at the Catholic University of Korea (CUMS-2019-0253-05 and CUMS-2021-0281-01).

### Data availability.

All supporting data are provided in the Supporting Data Values file or will be provided by the corresponding author upon request.

Other detailed experimental methods, including cell culture, Western blot, immunofluorescence staining, NFAT5 reporter assay, matrigel plug assay, ELISA, wound migration assay, boyden chamber assay, CCL2 promoter reporter construct and luciferase assay, flow cytometry, generation of air pouch model, induction of mBSA/IL1β-induced arthritis and SAA-accelerated arthritis, in vivo bioluminescence imaging and histological analysis of the joints, and real-time PCR, are described in the Supplemental Methods.

## Author contributions

ML, YMK, JP, and DK performed the experiments. WUK designed the experiments and analyzed the data. HMK and JHP provided the mice. ML, YMK, JHK, JJ, and DK contributed to the data analysis. ML, YMK, JHK, and DK drafted the paper. YP provided patient samples and information. WUK edited the paper. All authors commented on the manuscript. WUK coordinated the study design and implementation.

## Supplementary Material

Supplemental data

Unedited blot and gel images

Supporting data values

## Version 1 03/01/2024

Electronic publication
