# Supplementary material for: Serum amyloid A expression in liver promotes synovial macrophage activation and chronic arthritis via NFAT5
Source: J Clin Invest. 2024 Mar 1;134(5):e167835. doi: 10.1172/JCI167835 (PMC10904059; doi:10.1172/JCI167835)
Supplement: Supplemental data [file jci-134-167835-s125.pdf]

Supplemental Data Information

Serum amyloid A expression in liver promotes synovial macrophage activation and chronic arthritis via NFAT5

Meiling Li[1,2,3,a], Yu-Mi Kim[1,2,a], Jung Hee Koh[4], Jihyun Park[5], H. Moo Kwon[6], Jong-Hwan Park[7], Jingchun Jin[3,8], Youngjae Park[2,10], Donghyun Kim[5,9,11,b], and Wan-Uk Kim[1,2,10,b]

[1]Center for Integrative Rheumatoid Transcriptomics and Dynamics, The Catholic University of Korea, Seoul, Republic of Korea.
[2]Department of Biomedicine & Health Sciences, The Catholic University of Korea, Seoul, Republic of Korea.
[3]Department of Immunology of Yanbian University Hospital, Yanji, Jilin Province, China.
[4]Division of Rheumatology, Department of internal medicine, Uijeoungbu St.Mary's hospital, the Catholic Univ. of Korea, Uijeoungbu, Republic of Korea
[5]Department of Biomedical Sciences, Seoul National University College of Medicine, Seoul, Republic of Korea
[6]School of Nano-Bioscience and Chemical Engineering, Ulsan National Institute of Science and Technology, Ulsan, Republic of Korea
[7]Department of Laboratory Animal Medicine, College of Veterinary Medicine, Chonnam National University, Gwangju, Republic of Korea
[8]Key Laboratory of Science and Technology Department (Jilin Province), Cancer Research Center, Yanji, Jilin Province, China
[9]Department of Microbiology and Immunology, Seoul National University College of Medicine, Seoul, Republic of Korea
[10]Department of Internal Medicine, College of Medicine, The Catholic University of Korea, Seoul, Republic of Korea
[11]Institute of Endemic Diseases, Seoul National University Medical Research Center, Seoul, Republic of Korea

[a]These authors contributed equally to this work.
[b]Correspondence and reprint requests to: Dr. Wan-Uk Kim, Division of Rheumatology, Department of Internal Medicine, Catholic University of Korea, School of Medicine, Seoul, Republic of Korea (E-mail: wan725@catholic.ac.kr) or Dr. Donghyun Kim, Department of Biomedical Sciences, Seoul National University College of Medicine, Seoul, Republic of Korea (E-mail: biologokim@snu.ac.kr).

Short Title: Liver to joint connection via SAA

This file includes:

Supplemental Methods

Supplemental Figure 1. DAMP-induced increases in NFAT5 expression in RAW 264.7 macrophages.

Supplemental Figure 2. The representative image of NFAT5 localization in RAW 264.7 macrophages treated with SAA.

Supplemental Figure 3. No involvement of GPCR and FPRL1 in SAA-induced NFAT5 upregulation.

Supplemental Figure 4. Downregulation of NFAT5 expression by *Nfat5* shRNA or *Nfat5* knockout.

Supplemental Figure 5. NFAT5 regulation of MRP8-stimulated formation of filopodia and lamellipodia.

Supplemental Figure 6. Illustration of transwell migration assay.

Supplemental Figure 7. TLR2/4 dependency of SAA-induced macrophage migration.

Supplemental Figure 8. Experimental design for determining effect of *Saa1* overexpression on arthritis progression.

Supplemental Figure 9. Immunofluorescence colocalization for CD90 (green) or NIMP-R14 (green) with CCL2 (red) in the affected joints of $Nfat5^{fl/fl}$ versus *LysM*-Cre; $Nfat5^{fl/fl}$ mice with SAA-accelerated arthritis.

Supplemental Figure 10. IL-6 reversal of the decrease in arthritis severity by NFAT5 deficiency

Supplemental Figure 11. Hepatic overexpression of m*Saa1* in healthy mice by adenovirus-mediated transfer of luciferase-tagged m*Saa1*.

Supplemental Figure 12. The levels of serum SAA at baseline and 6-months after treatment with anti-rheumatic drugs

Supplemental Figure 13. Hypothetical model of interorgan communication between liver and joint via SAA-TLR2/4-NFAT5 axis to accelerate chronic arthritis.

Supplemental Table 1. Demographics and patients' characteristics at baseline

Supplemental References

Supplemental Methods

RAW 264.7 and transformed RAW 264.7 cells

RAW 264.7 cells were obtained from ATCC (Manassas, VA) and maintained in RPMI 1640 (Gibco) containing 10% fetal bovine serum (FBS, Gibco). RFP-NFAT5 reporter RAW 264.7 cells (stably transfected with NFAT5-dependent red fluorescence protein (RFP) reporter plasmid), NFAT5 KD cells (transfected with short hairpin RNA [shRNA] against *Nfat5* mRNA), and GFP-expressing cells (transfected with GFP-expressing plasmid) were previously established (1).

In vitro cell experiments

To investigate the effect of SAA on NFAT5 expression and activation *in vitro*, cells in wells of 24- or 48-well plates were treated with indicated concentrations of SAA (Peprotech) for 24 hours (or indicated period). To determine which receptor and signaling pathways are required for the SAA-mediated NFAT5 induction, RAW 264.7 cells were pretreated with small molecule inhibitors (TAK-242, SB203580, PD98059, SP600125, LY294002, Wortmannin, and allopurinol from Sigma; OxPAPC from InvivoGen) 30 minutes before SAA (5 μg/mL) stimulation. Additionally, RAW 264.7 cells were transfected with 5 nM of siRNAs for *Jnk1/2, Tlr2, Tlr4,* or scrambled sequence (Santa Cruz Biotechnology) using Lipofectamine 3000 (Thermo Fisher Scientific) according to manufacturer's instructions. After 24 hours, the medium was replaced with fresh and transfected cells were treated with SAA.

Western blot assay

Cells were lysed in RIPA buffer (50 mM Tris-HCl pH7.6, 150 mM NaCl, 1%

NP-40, 0.5% sodium deoxycholate, and 0.1% SDS supplemented with complete protease inhibitor) for 10 minutes at 4°C. The cell lysates were spun down at 20,000 x *g* for 15 minutes at 4°C and the supernatants were collected. Protein samples were electrophoresed on 7, 10, or 12.5% SDS-polyacrylamide gels, followed by electrotransfer to PVDF (BioRad). Membranes were blocked for 1 hour at room temperature in T-TBS containing 5% skim milk (BD Biosciences) and then incubated with anti-NFAT5 antibody (Ab) (diluted 1:3,000; courtesy of Dr. H. Moo Kwon, Ulsan National Institute of Science and Technology), anti-p38 Ab, anti-JNK1/2 Ab, anti-phosphorylated p38 Ab, anti-phosphorylated ERK1/2 Ab, anti-phosphorylated JNK1/2 Ab, anti-phosphorylated AKT Ab (1:1,000 for all; 9212, 9252, 9211, 9101, 9251, 9271, respectively, Cell Signaling Technology), anti-AKT, anti-β actin, and anti-GAPDH (sc-8312, sc-47778, and sc-25778, respectively, Santa Cruz Biotechnology) in T-TBS containing 5% skim milk overnight at 4°C. After washing three times in T-TBS, the membranes were incubated for 2 hours at room temperature with a peroxidase-conjugated goat anti-rabbit or anti-mouse Ab (1:1,000; sc-2004 or sc-2005, Santa Cruz Biotechnology) in T-TBS containing 5% skim milk. They were then washed three times in T-TBS, and exposed to ECL chemiluminescent substrate (Pierce). The chemiluminescence signal was detected using a Luminescent Image Analyzer LAS-4000 (Fuji Film) or X-ray films.

Immunofluorescence staining

Fixed cells and synovial tissue sections were blocked and incubated with the following primary Abs: anti-NFAT5 (1:3,000), anti-SAA (1:500; ab687, Abcam), anti-CD14 (1:1,000; ab45870, Abcam), anti-F4/80 (1:3,000; MCA497G, Bio-Rad), anti-

CCL2 (1:3,000; ab308523, Abcam or NBP2-22115, Novus Biologicals), CD90 (1:1,000; sc-53116, Santa Cruz Biotechnology), NIMP-R14 (1:3,000; sc-59338, Santa Cruz Biotechnology), and anti-RFP (1:1,000; ab62341, Abcam). Appropriate Alexa488-, Alexa594-, or Alexa647-conjugated secondary Abs (A-21202, A-21206, A-21208, A-11058, A-21203, A-21207, A-21472, or A-31573, Invitrogen) were used for fluorescent detection. Rhodamine phalloidin (Invitrogen) was used to label F-actin in cells. Nuclei were then stained with 4',6-diamidino-2-phenylindole (DAPI; Roche). The samples were mounted with antifade mounting medium (Vector Labs) and observed under an LSM510 or LSM 900 confocal microscope (Carl Zeiss). Images were processed with ZEN lite (Carl Zeiss).

NFAT5 reporter assay

RAW 264.7 cells were transfected with luciferase-fused NFAT5 reporter genes (1.3 μg) using Neon Transfection System (Thermo Fisher Scientific) as previously described (1). The reporter plasmid transfecting cells (5×10^4 cells/well of 48-well plates) were plated in DMEM (Gibco) supplemented with 10% FBS, L-glutamine, sodium pyruvate, and antibiotics. The next day, the transfected cells were treated with SAA for 18 hours and lysed with passive lysis buffer (Promega). The luciferase activities were measured using the Luciferase Assay System according to the manufacturer’s instructions (Promega).

Matrigel plug assay

To measure DAMP-mediated NFAT5 activation *in vivo*, Matrigel plug reporter assays were performed as previously described (1). Briefly, Matrigel complexes (0.6

mL; BD Biosciences) containing RFP-NFAT5 reporter RAW 264.7 cells (1 x 10^7 cells/mL) were subcutaneously injected into the dorsal regions of male BALB/c mice. Nine days after implantation of Matrigels, the mice were intraperitoneally injected with SAA (10 μg/mL). After 16 hours, Matrigel plugs were removed and their fluorescence intensity was measured by Maestro 2 imaging system (CRi). In some experiments, SAA (10 μg/mL) was locally treated to Matrigels containing RFP-NFAT5 reporter RAW 264.7 cells (1 x 10^7 cells/mL) prior to implantation into mice. After 24 hours of implantation, Matrigel plugs were collected and their fluorescence intensities were measured using Maestro software 2.8 (CRi). The RFP activity were calculated as total photons per centimeter squared per steradian ($\times 10^6$ p/s/cm^2/sr).

ELISA

The levels of SAA (MyBioSource), IL-6 (R&D systems), and CCL2 (R&D systems) were measured using commercial ELISA kits according to manufacturer's instructions.

Wound migration assay

The intrinsic motility of cells was evaluated using wound migration assay. Briefly, NFAT5 KD RAW 264.7 cells and control RAW 264.7 cells (1.5 x 10^6 cells/well) were seeded. Next day, monolayers were scratched with a pipette tip to generate a "wound gap" and were treated with or without 2.5 μg/mL of SAA. After 16 hours, the cells were fixed and stained according to the manufacturer's instructions and the number of migrated cells into the wound gap was measured.

Boyden chamber assay

Chemotaxis assays were performed using a Boyden chamber (8.0-μm pores size, Corning). RAW 264.7 cells or primary macrophages (2 x 10^5 cells/well) were suspended in the serum-free medium and seeded in the upper chamber of Transwell invasion plates. SAA (5 μg/mL), recombinant IL-6 (10 ng/mL; R&D systems), and recombinant CCL2 (10 ng/mL; R&D systems) were added into the upper chamber; fresh media supplemented with 1% FBS was placed in the lower chamber. In some experiments, conditioned media of SAA-treated macrophages were used. Briefly, RAW 264.7 cells or primary macrophages were cultured with or without SAA (5 μg/mL) for 8 hours, and the culture supernatant were collected. For migration assay, the cells (1 x 10^5 cells/well) were seeded in the upper chamber while the conditioned media was placed in the lower chamber as a chemoattractant. To determine the effect of IL-6 and CCL2, the neutralizing Abs of IL-6 and CCL2 (1 μg/mL; R&D systems) were added to the conditioned media of SAA-treated macrophages. After 8 hours, the cells that failed to invade in the upper side were gently removed using cotton swabs, and the remaining invaded cells were stained with crystal violet for cell counting.

CCL2 promoter reporter construct and luciferase assay

A mouse CCL2 promoter sequence was generated by PCR with genomic DNA extracted from RAW 264.7 cells as a template, along with a primer pair (5′-GCAGAGCCACTCCATTCACA-3′ and 5′-GGTGGTGGAGGAAGAGAGAG-3′) for the -315 ~ +85 DNA sequence, which encompasses the NFAT5-binding site (2, 3). The PCR product for the CCL2 promoter DNA was purified and inserted into a pGL4.10_luc2 vector. The cloned CCL2 reporter plasmid was transfected by using

Lipofectamine LTX with Plus Reagent (Invitrogen) into *Nfat5*-deficient and sufficient RAW 264.7 cells. The transfected cells were cultured overnight at 37 °C with 5 % CO2 and then treated with SAA. After 18 hours, the cell pellet was harvested, and a luciferase assay was conducted using a commercial kit (Luciferase Reporter Assay System, Promega) following the manufacturer's provided protocol. The luminescence was measured by the basic protocol of the GloMax Microplate Reader (Promega).

Flow cytometry

Cells isolated from synovial fluids were stained with fluorescence-labeled Abs for CD14 (12-0149-42, eBioscience). After the wash, the cells were fixed and permeabilized using Intracellular Fixation & Permeabilization Buffer (eBioscience). An Ab for NFAT5 and a secondary fluorescence-labeled Ab (11-4839-81, eBioscience) were added sequentially. The fluorescence of stained cells or GFP-expressing cells was measured using a FACS Canto II Flow System (BD Biosciences) instrument, and data was analyzed using FlowJo software (TreeStar).

Air pouch model

To evaluate the role of NFAT5 in SAA-mediated monocyte/macrophage migration *in vivo*, the air pouch model was introduced in mice. Briefly, air (3 mL) was subcutaneously injected into the backs of BALB/c mice. After 3 days, 3 mL of air and SAA (20 μg/mL) were injected into the pouches, and GFP-expressing RAW 264.7 cells (5 x 10^5 cells/mouse) were then intravenously injected on the same day. After 2 days, the mice were anaesthetized, and the pouches were washed with 1 mL of PBS. The number of GFP-expressing cells in the lavage fluids was analyzed using flow cytometry.

mBSA/IL1β-induced arthritis model, SAA-accelerated arthritis model, and recombinant CCL2 injection

Mice were intra-articularly treated with methylated bovine serum albumin (mBSA; 200 μg/mouse; Sigma) with or without SAA (5 μg/mouse) on day 0. Recombinant IL1β (rIL1β; 250 ng/mouse; R&D Systems) was then subcutaneously injected into the footpad twice, 1 and 2 days after mBSA/SAA treatment. A separate control cohort of mice was used where a vehicle was injected instead of SAA but otherwise with the same procedure as SAA-accelerated arthritis. On day 7, the mice were euthanized, and joint tissues were collected. The mice with mBSA/IL1β-induced arthritis that additionally received SAA were defined as the SAA-accelerated arthritis model. In some experiments, recombinant CCL2 (2 μg/mouse) or recombinant IL-6 (2 μg/mouse) was injected into a joint of *LysM*-Cre;*Nfat5*$^{fl/fl}$ mice three times at 3, 5, and 7 days after mBSA/SAA treatment. The mice were euthanized on day 8, and joint tissues were harvested. To investigate the pathologic role of SAA in inflammatory arthritis *in vivo*, neutralizing antibodies to SAA or control IgG (1 mg/kg; Invitrogen) were intraperitoneally injected into the mice with mBSA/IL1β-induced arthritis on day 1 or day 2.

LPS injection

LPS (10 mg/kg) was intraperitoneally injected into C57BL/6 mice. The next day mice were sacrificed, and their joints and liver were collected.

In vivo bioluminescence imaging

Adenoviral vectors harboring mSaa1 gene (Ad-m*Saa1*) or control vectors (Ad-

Con) were generated by Sirion Biotech and injected into C57BL/6 mice via tail vein. Bioluminescence imaging was conducted in the mice at -2, 1, 4, and 7 days after intravenous injection of Ad-m*Saa1* or Ad-Con. For this, the mice were injected intraperitoneally with 150 mg/kg Xenolight d-luciferin potassium salt (PerkinElmer) diluted in PBS and left to rest for 5 minutes. Subsequently, mice were anesthetized by 1.5-2% isoflurane (Ifran liquid, Hana. Pharm Co) and bioluminescence images were acquired using an IVIS Lumina XRMS Series III system (PerkinElmer). Images were analyzed with Living Image v4.4 software (PerkinElmer). An ROI was drawn around the whole mouse. The luciferase activity was expressed as photons per second.

Histological analysis

The collected knee joints were fixed in 4% paraformaldehyde (Biosesang) overnight at 4°C. The joints were then decalcified with Decalcifying Solution-Lite (Sigma-Aldrich) for 36 hours at room temperature prior to embedding in paraffin. Sagittal sections of joint (thickness of 4-4.5 μm) were cut at five depths approximately 100 μm apart and stained with hematoxylin and eosin (H&E), Safranin O-fast green or toluidine blue. The slides were qualitatively evaluated using the following parameters: synovial hyperplasia, inflammation, and bone destruction. Disease severity was graded histologically for each from 0 (normal) to 3 (severe) by an investigator blinded to the experimental groups(4). The extent of cartilage depletion was assessed by the criteria as described previously (5)

For immunohistochemistry, the paraffin-embedded sections were hydrated with graded ethanol and rinsed in tap water. Antigen was retrieved by citric acid buffer (pH 6.0) microwave antigen retrieval. They were then incubated for 30 minutes with

methanolic hydrogen peroxide, rinsed in tap water, and treated with 0.5% Triton X-100 (Sigma) in PBS for 15 minutes. The sections were then rinsed in PBS three times for 10 minutes before being treated with normal donkey serum (Jackson ImmunoResearch Laboratories) for 1 hour and incubated with anti-F4/80 (1:3,000; MCA497G, Bio-Rad), anti-NIMP-R14 (1:3,000; sc-59338, Santa Cruz Biotechnology), anti-CCL2 (1:3,000; ab308523, Abcam), and anti-vWF Abs (1:2000; ab194405, Abcam) overnight at 4°C. After being washed in PBS, the tissue sections were incubated for 2 hours in biotin-conjugated donkey anti-rat IgG or anti-rabbit IgG secondary Ab (1:200; 712-065-153 or 711-065-152, respectively, Jackson ImmunoResearch Laboratories), followed by Vectastain ABC Kit (Vector Laboratories). For the detection of peroxidase, 3,3′-diaminobenzidine tetrahydro-chloride (DAB; Vector Laboratories) was used as the chromogen. The sections were subsequently counterstained with hematoxylin and mounted with Surgipath MM24 mounting medium (Leica). The slides were scanned using a Pannoramic MIDI digital slide scanner (3DHISTECH), and images were extracted by Case Viewer software (3DHISTECH). The number of cells stained with F4/80, NIMP-R14, and CCL2 Ab was carefully quantified by reading three randomly chosen fields in the high-power field on each slide. For each animal, three sections were counted from the joint. The values are expressed as a total number of cells (as percentage) in the respective slide.

Quantitative real-time PCR

Total RNA was extracted using RNeasy Mini Kit (QIAGEN) and then cDNA was generated from the isolated RNA using a RevertAid First Strand cDNA Synthesis Kit (Thermo Fisher Scientific) according to the manufacturer’s instruction. Real-time

qPCRs were performed with the cDNA, specific primer sets, and SYBR Green Master Mix (Bio-Rad) in a MyCycler (Bio-Rad). The real-time qPCR primers (Bioneer) were as follows: human *NFAT5* (5'-CCAGAAGTCATTTGCCTGGT-3'; 5'-GATTCCAAGC-CCACTCTTCA-3'), human *GAPDH* (5'-AAGGTGAAGGTCGGAGTCAA-3'; 5'-AA TGAAGGGGTCATTGATGG-3'), mouse *Nfat5* (5'-AACCAGTGGTGTTGCAGGTA-3'; 5'-AGGGAGTTGTATTTCGCCCAG-3'), mouse *Saa1* (5'-TGTTCACGAGGCTTT -CCAAG-3'; 5'-ATTTGTCTGAGTTTTTCCAGTTAGC-3'), mouse *Saa2* (5'-CTGCCTGCCAA -ATACTGAGAGTC-3'; 5'-CCACTTCCAAGTTCCTGTTTATTAC-3'), mouse *Saa3* (5' -GCTGGCCTGCCTAAAAGATACTG-3'; 5'-GCATTTCACAAGT -ATTTATTCAGC-3'), mouse *Tlr2* (5'-ACAGCAAGGTCTTCCTGGTTCC-3'; 5'-GCTCCCTTACAGGCTGAGTTCT-3'), mouse *Tlr4* (5'- AGCTTCTCCAATTTTTCA-GAACTTC-3'; 5'-TG-AGAGGTGGTGTAAGCCATGC-3') and mouse *Gapdh* (5'-AGGTCGGTGTGAACGGATTTG-3'; 5'-TGTAGACCATGTAGTTGAGGTCA-3'). The qPCR conditions for mRNA quantification were 95°C for 3 minutes, followed by 40 cycles of denaturation at 95°C for 10 seconds and annealing and extension at 60°C for 30 seconds. The cycle threshold values of each sample were normalized by the value of GAPDH.

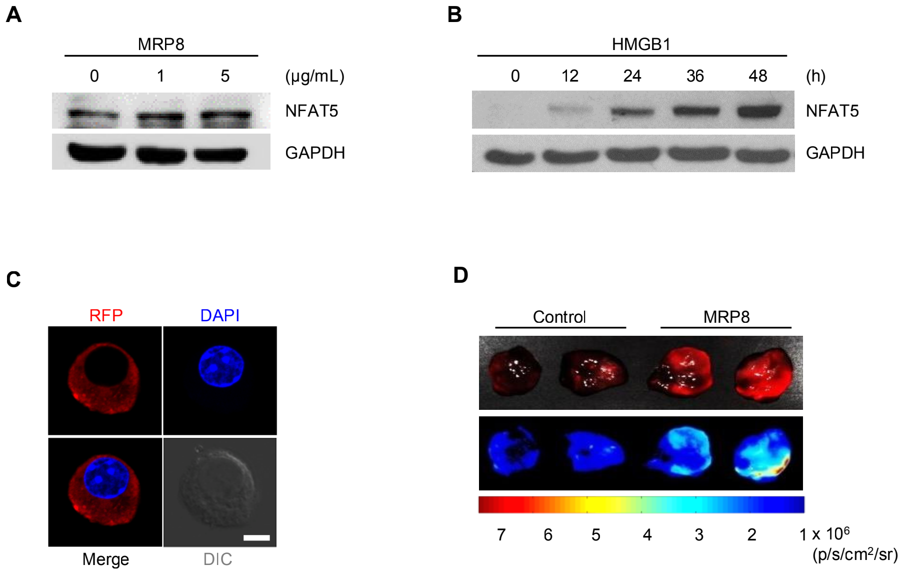

Supplemental Figure 1. DAMP-induced increases in NFAT5 expression in RAW 264.7 macrophages. (**A**, **B**) Western blot analysis of NFAT5 in RAW 264.7 macrophages after treatment with MRP8 (1, 5 µg/mL) (A) and HMGB1 (200 ng/mL) (B). β-actin or GAPDH served as a loading control. Data are representative of at least 3 independent experiments with similar results. (**C**) Immunofluorescent imaging of RAW 264.7 macrophages harboring NFAT5-dependent reporter tagged with RFP using anti-RFP Ab. Nuclei were counterstained with DAPI (blue). Scale bar: 5 µm. (**D**) Increase in NFAT5 activity by MRP8. Mice were subcutaneously implanted with Matrigels containing MRP8 (40 µg/mL) and RAW 264.7 macrophages transfected with NFAT5-dependent RFP reporter. After 24 hours, the Matrigels were harvested and subjected to RFP imaging

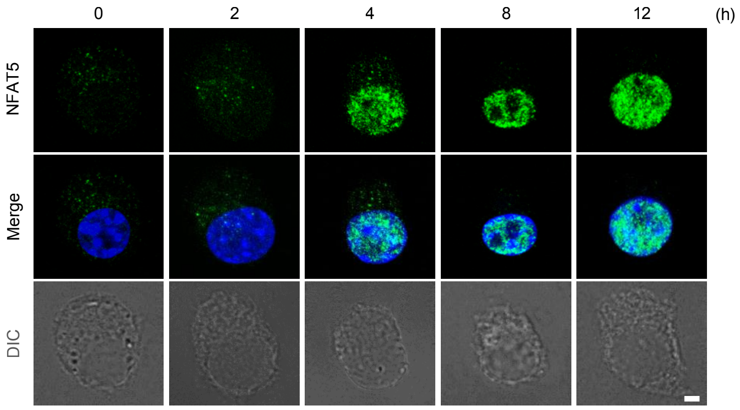

Supplemental Figure 2. The representative image of NFAT5 localization in RAW 264.7 macrophages treated with SAA. Nuclei were counterstained with DAPI (blue). Scale bars: 5 μm.

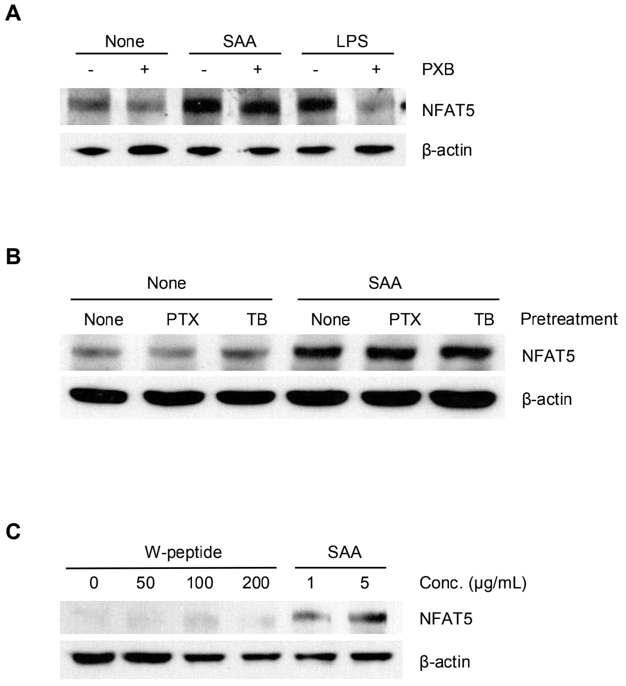

Supplemental Figure 3. No involvement of GPCR and FPRL1 in SAA-induced NFAT5 upregulation. (**A**) NFAT5 expression in RAW 264.7 macrophages pretreated with polymyxin B (PXB) in the presence of SAA or LPS. (**B**) No effect of pertussis toxin B (PTX) and toxin B (TB) on SAA-induced increase in NFAT5 expression in RAW 264.7 macrophages. (**C**) No effect of W-peptide (a specific agonistic peptide of FPRL1) on SAA-triggered NFAT5 upregulation. RAW 264.7 macrophages were stimulated with SAA (A and B, 5 μg/mL; C, 1 and 5 μg /mL) for 24 hours and then subjected to Western blot analysis. β-actin served as a loading control. Data are representative of at least three independent experiments with similar results.

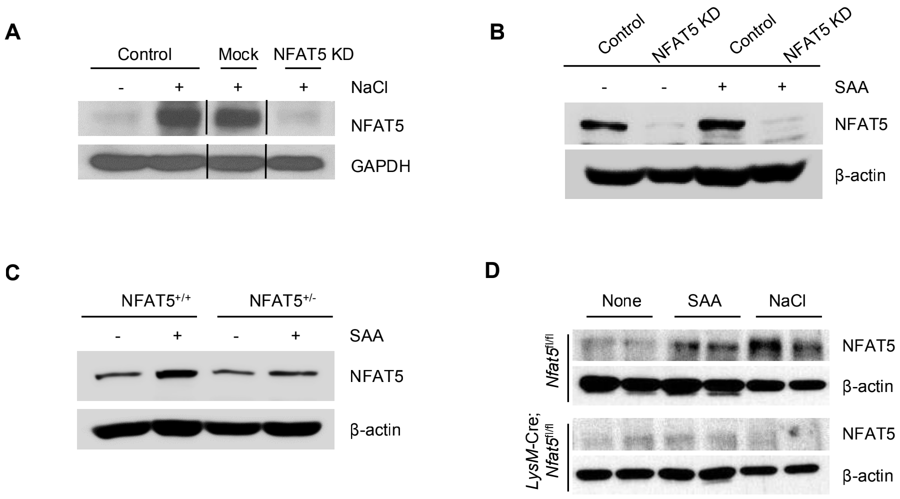

Supplemental Figure 4. Downregulation of NFAT5 expression by *Nfat5* shRNA or *Nfat5* knockout. (**A**, **B**) Decrease in NFAT5 expression in RAW 264.7 macrophages 24 hours after transfection of *Nfat5* shRNA (NFAT5 KD)**.** After the transfection of *Nfat5* shRNA, the cells were stimulated with NaCl or SAA for 18 hours. NFAT5 expression was determined by Western blot analysis. (**C, D**). Decrease in NFAT5 expression in macrophages of NFAT5-deficient mice. Recombinant SAA (5 μg/mL) was treated to BMDM of NFAT5-haploinsufficent mice (NFAT5$^{+/-}$) and peritoneal macrophages of *LysM*-Cre; *Nfat5$^{fl/fl}$* mice for 24 hours. BMDM of wild type mice (NFAT5$^{+/-}$) and peritoneal macrophages of *Nfat5$^{fl/fl}$* mice were used as controls, respectively. The NFAT5 expression was determined by Western blot analysis. The representative of more than three independent experiments was shown.

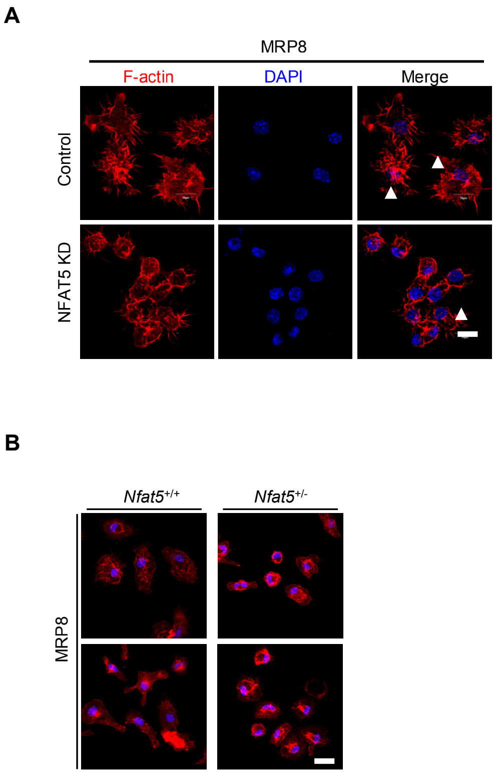

Supplemental Figure 5. NFAT5 regulation of MRP8-stimulated formation of filopodia and lamellipodia. (A, B) Immunofluorescence imaging by confocal microscopy of F-actin (red) in RAW 264.7 macrophages after stable transfection of NFAT5 shRNA (NFAT5 KD) (A) and in peritoneal macrophages of *Nfat5*$^{+/-}$ mice (B), when stimulated with MRP8 (5 μg/mL) for 2.5 hours. Nuclei were counterstained with DAPI (blue). Arrows indicate filopodia and lamellipodia. Scale bars: 10 μm.

A

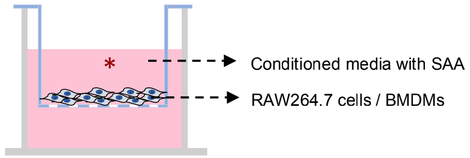

B

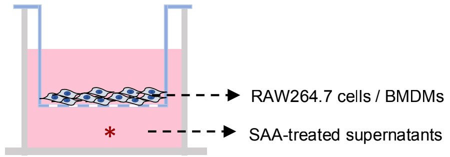

Supplemental Figure 6. Illustration of transwell migration assay. (**A**) RAW 264.7 macrophages and mouse BMDMs were stimulated with SAA (5 μg/mL) in the upper chamber for 8 and 16 hours, respectively. The number of migrated cells was counted manually. (**B**) Conditioned media were obtained from cultured RAW 264.7 macrophages or mouse BMDMs 24 hours after stimulation with SAA (5 μg/mL) and then added to the lower chamber to stimulate chemotactic migration. Migrated cells were counted 8 and 16 hours after addition of corresponding conditioned media.

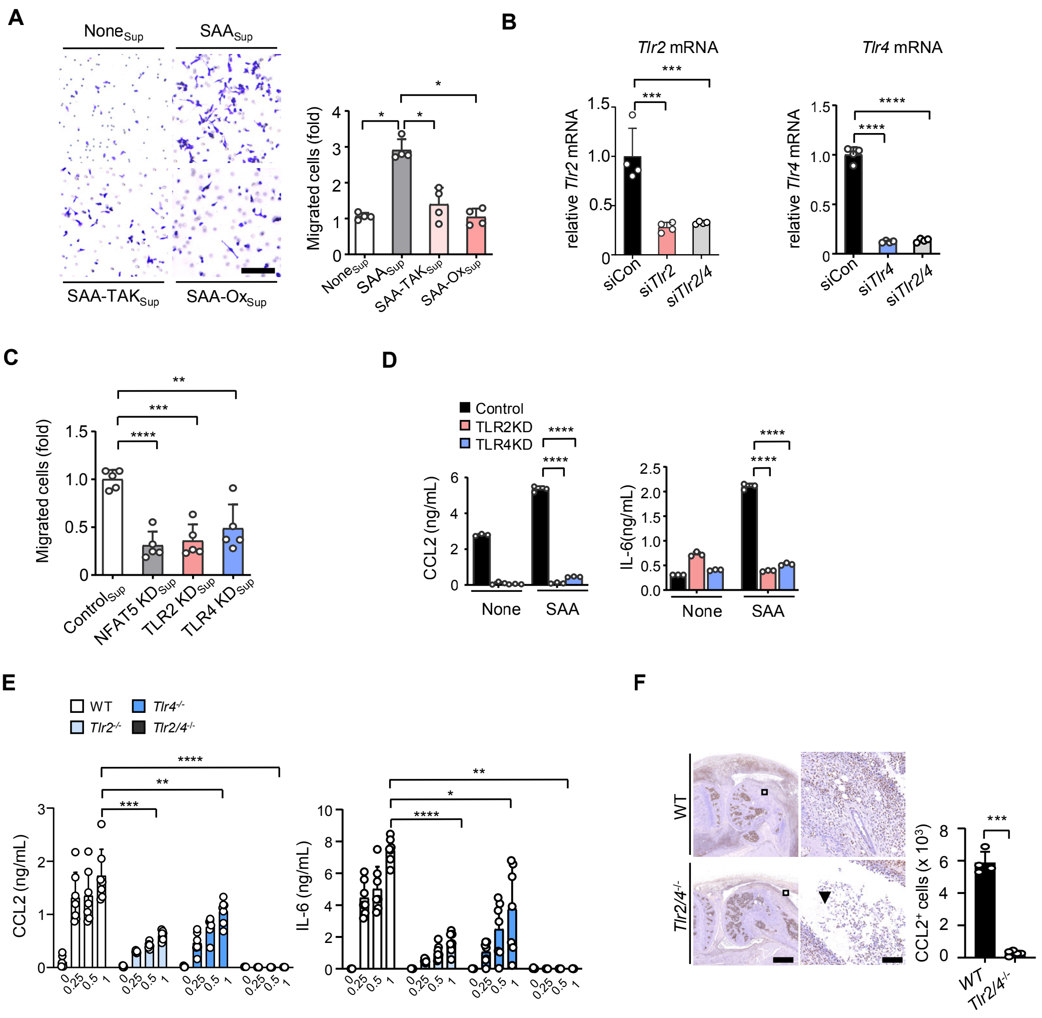

Supplemental Figure 7. TLR2/4 dependency of SAA-induced macrophage migration. (**A**) Inhibition of SAA-induced chemotactic migration of macrophages by TLR2/4 antagonists. Conditioned media were harvested from cultured RAW 264.7 macrophages 24 hours after stimulation with SAA (5 µg/mL), in the absence (SAA_{Sup}) or presence of TLR4 antagonist TAK-242 ($SAA\text{-}TAK_{Sup}$) and TLR2/4 antagonist OxPAPC ($SAA\text{-}Ox_{Sup}$). Chemotactic migration of RAW 264.7 macrophages was then induced by adding conditioned media to the lower chamber. After 5 hours, the number of migrated cells was counted manually. Scale bar: 100 µm. (**B**) Real time-qPCR analysis of TLR2 and TLR4 expression in RAW 264.7 macrophages after transient transfection of siRNAs for *Tlr2* (TLR2 KD), *Tlr4* (TLR4 KD), and *Tlr2/4* (TLR2/4 KD). Data are representative of at least 3 independent experiments. (**C**) Suppression of SAA-

induced macrophage migration by *Tlr*2/4 deficiency. Conditioned media were harvested from RAW 264.7 macrophages stimulated with SAA (5 μg/mL) for 5 hours after stable transfection with *Nfat5* shRNAs (NFAT5 KD $_{Sup}$), *Tlr2* shRNAs (TLR2 KD$_{Sup}$), and *Tlr4* shRNAs (TLR4 KD$_{Sup}$). Chemotactic migration of RAW 264.7 macrophages was conducted in the presence of the conditioned media for 5 hours. (**D, E**) ELISA for IL-6 and CCL2 level in culture supernatants of TLR2 KD and TLR4 KD RAW 264.7 cells stimulated with SAA (2.5 μg/mL) for 5 hours (D) or in *Tlr2*, *Tlr4*, and *Tlr2/4* knockout (KO) peritoneal macrophages stimulated with SAA (0.25, 0.5, and 1 μg/mL) for 24 hours (E). (**F**) Immunohistochemical analysis of CCL2 expression in the affected joints of WT and *Tlr2/4* double knockout mice (*Tlr2/4*$^{-/-}$) with SAA-accelerated arthritis (*See* **Supplemental Figure 7A**). Scale bars: 500 μm for low magnification (left) and 50 μm for high magnification (right). Bar graphs in A to F indicate mean ± SD. **P* < 0.05, ***P* < 0.01, ****P* < 0.001, and *****P* < 0.0001 by Kruskal-Wallis test and Mann-Whitney's post hoc test for (A) and IL-6 ELISA in (E), by one-way ANOVA with Tukey's multiple comparisons test for (B), (C), and (D), by Brown-Forsythe and Welch ANOVA with Dunnett T3 multiple-comparison test for CCL2 ELISA in (E), and by Welch’s t-test for (F).

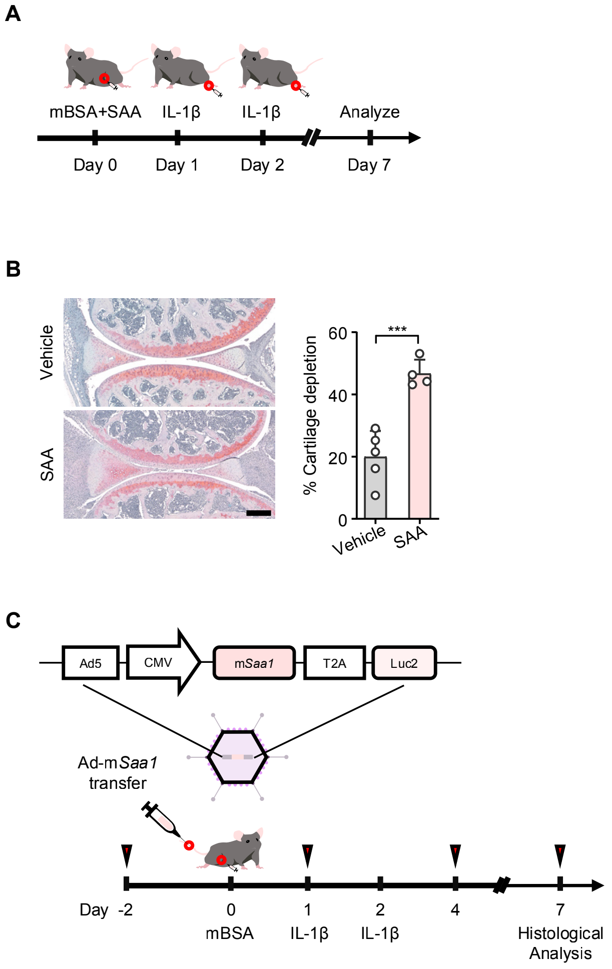

Supplemental Figure 8. Experimental design for determining effect of *Saa1* overexpression on arthritis progression. (A) SAA-accelerated arthritis was induced by the following protocol; on day 0, SAA (5 μg) plus methylated bovine serum albumin (mBSA, 200 μg) were injected intra-articularly into the knee joint. On days 1 and 2, IL-1β (250 ng) was injected into the footpad on the same side. On day 7, the mice were sacrificed and assessed for severity of joint inflammation and infiltration of F4/80^{+} and NIMP-R14^{+} cells. (**B**) Arthritic joints of mice with or without SAA-accelerated arthritis were stained with Safranin O on day 7. Cartilage damage was assessed by the degree of cartilage depletion. The bar graph on the right shows mean ± SD of the degree of cartilage damage in the two groups. ***$P < 0.001$ by Welch's t-test. Scale bar: 200 μm. (**C**) Experimental design to determine the effect of *Saa1* overexpression in the liver on arthritis progression. On day 0, adenoviral vectors harboring m*Saa1* (Ad-m*Saa1*) or control (Ad-Con) were injected intravenously into the mice and then 200 μg of mBSA

was injected intra-articularly into the knee joint. On days 1 and 2, IL-1β (250 ng) was injected into the footpad on the same side as the affected joint. On day 7, the mice were sacrificed and evaluated for arthritis severity. Red arrows indicate the day when *in vivo* luciferase imaging was carried out.

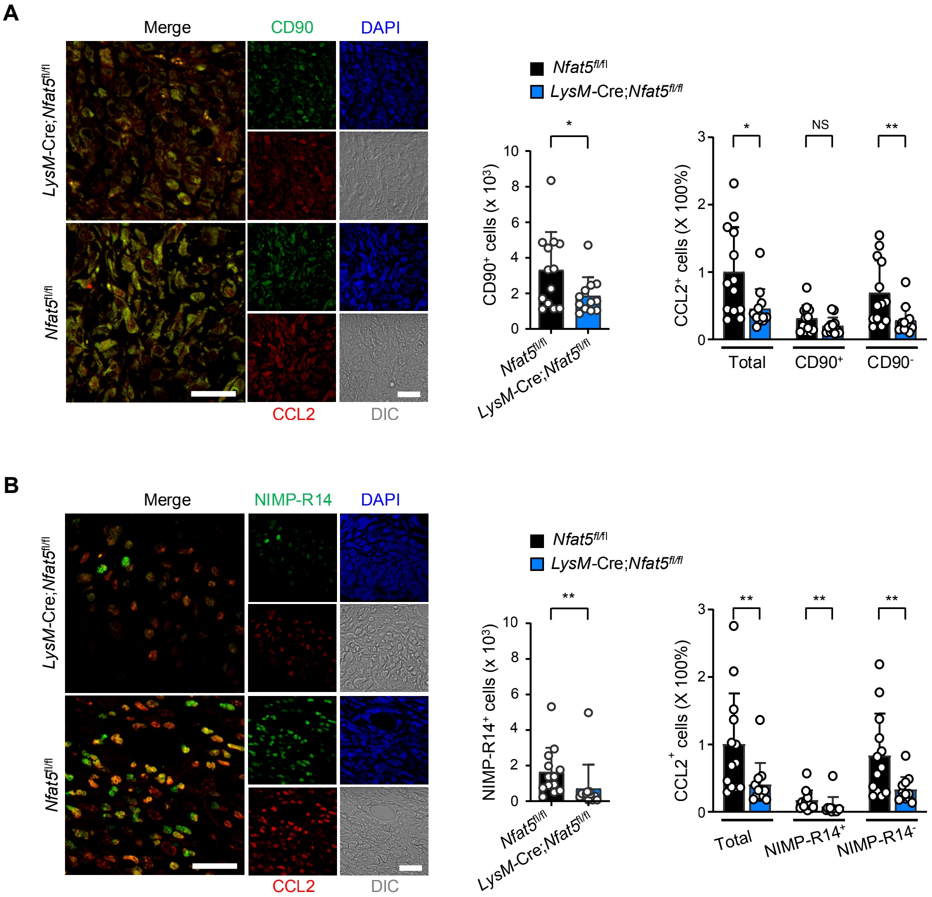

Supplemental Figure 9. Immunofluorescence colocalization for CD90 (green) or NIMP-R14 (green) with CCL2 (red) in the affected joints of *Nfat5$^{fl/fl}$* versus *LysM*-Cre; *Nfat5$^{fl/fl}$* mice with SAA-accelerated arthritis. Nuclei were counterstained with DAPI (blue). The bar graphs represent mean ± SD. *P < 0.05 by Mann Whitney U test for immunostaining of $CD90^+$ cells or NIMP-$R14^+$ cells and Kruskal-Wallis test with post hoc Mann-Whitney U test for immunostaining of $CCL2^+$ cells. Scale bars: 20 µm.

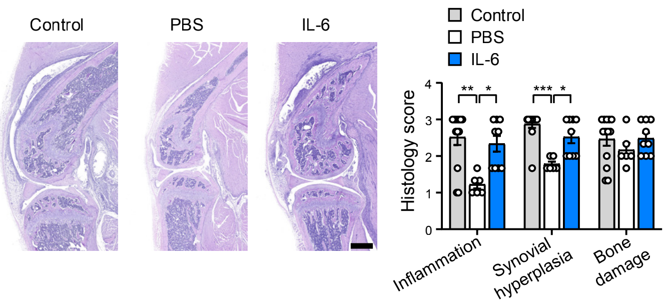

Supplemental Figure 10. IL-6 reversal of the decrease in arthritis severity by NFAT5 deficiency. Histological severity of the affected joints of *LysM*-Cre; *Nfat5*$^{fl/fl}$ mice injected intra-articularly with IL-6 (2 μg × 3) versus vehicle alone. The *Nfat5*$^{\mathrm{fl/fl}}$ mice with SAA-accelerated arthritis were used as a positive control. Mean histological severity is shown on the right. Data are mean ± SD. **P* < 0.05, ***P* < 0.01, and ****P* < 0.001 by Kruskal-Wallis test with a Dunn's multiple comparisons test. Scale bars: 1mm.

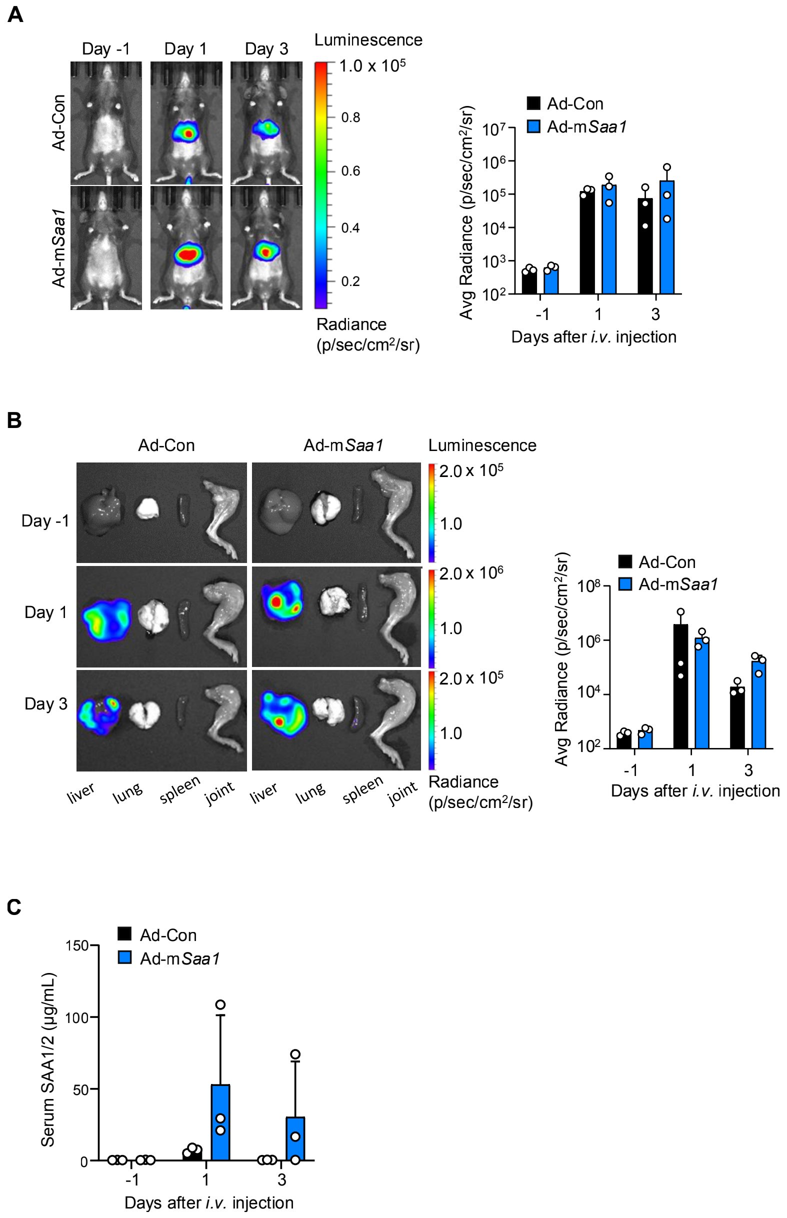

Supplemental Figure 11. Hepatic overexpression of m*Saa1* in healthy mice by adenovirus-mediated transfer of luciferase-tagged m*Saa1*. (**A**) *In vivo* luciferase imaging of whole bodies of mice with no arthritis, before and after intravenous injection of Ad-m*Saa1* and Ad-Con. Mean bioluminescence signal intensity (p/sec/cm^2/sr) in the liver at different time points is shown on the right. (**B**) The *ex vivo* luciferase imaging of liver, lung, spleen, and joint of healthy mice before and after intravenous injection of Ad-*mSaa1* and Ad-Con. Mean bioluminescence signal intensity (p/sec/cm^2/sr) in the liver at different time points is shown on the right. (**C**) SAA1/2 levels in the sera of

healthy mice before and after injecting Ad-m*Saa1* versus Ad-Con, determined by ELISA. Bar graphs in A to C represent mean ± SD. $^{*}P < 0.05$, $^{**}P < 0.01$, $^{***}P < 0.001$, and $^{****}P < 0.0001$ by two-way ANOVA with Sidak's multiple comparisons test (A, C) and Friedman's test with Dunn's multiple-comparisons test (B).

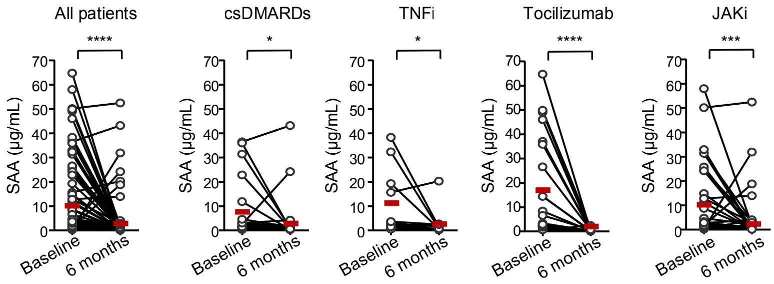

Supplemental Figure 12. The levels of serum SAA at baseline and 6-months after treatment with anti-rheumatic drugs. *$P < 0.05$, ***$P < 0.001$, and ****$P < 0.0001$ by Wilcoxon matched pairs signed rank test.

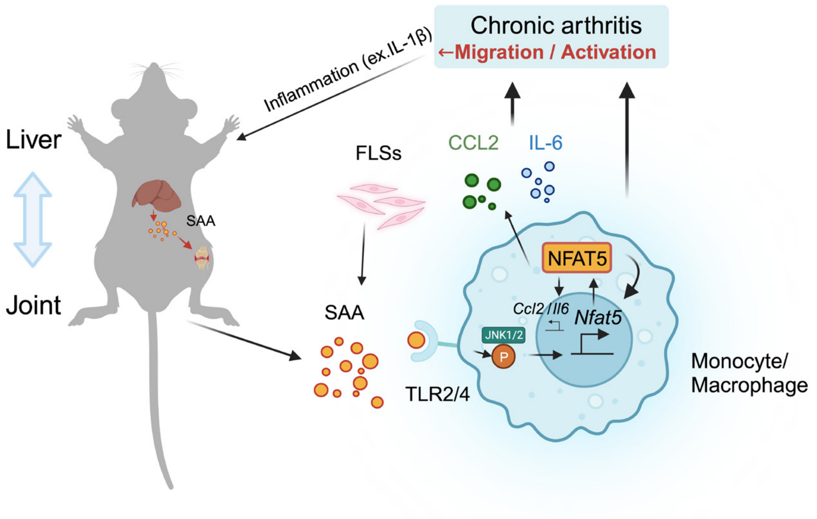

Supplemental Figure 13. Hypothetical model of interorgan communication between liver and joint via SAA-TLR2/4-NFAT5 axis to accelerate inflammatory arthritis. Under nonspecific inflammatory conditions, IL-1β is generated in the joints and stimulates production of SAA in the liver. Liver-generated SAA, can in turn, go to the affected joints via circulatory system whereupon it activates TLR2/4-NFAT5 axis in synovial macrophages to secrete pro-inflammatory cytokines and chemokines, including IL-6 and CCL2; since the SAA level in the sera exceeds that in synovial fluids in RA patients (**Figure 8A**), such activation by SAA might occur in other blood-rich organs in RA. The resultant cytokines and chemokines can amplify inflammatory arthritis by further activating immune cells in autocrine and/or paracrine manners, including synovial macrophages, neutrophils, and fibroblasts in the joints and by facilitating recruitment of monocytes to inflamed joints. Meanwhile, various DAMPs (including SAA) locally generated in inflamed joints can directly promote NFAT5 activity, bolstering proinflammatory responses triggered by liver-originated SAA-TLR2/4-NFAT5 axis. As a result, more activated synovial monocytes/macrophages produce greater amounts of IL-1β to may further stimulate SAA secretion from the liver, which could lead to an SAA-centered mutual activation of liver and joints. FLSs=fibroblast-like synoviocytes. The graphical abstract was created with BioRender.com.

Supplemental Table 1. Demographics and patients' characteristics at baseline

	csDMARDs (N = 25)	TNFi (N = 11)	Tocilizumab (N = 23)	JAKi (N = 44)	*P*
Age, year (IQR)	49 (46–57)	64 (35–66)	51 (39–61)	59 (50–65)	0.092
Female, n (%)	24 (96.0)	10 (90.9)	19 (82.6)	39 (88.6)	0.511
Diabetes, n (%)	0 (0.0)	1 (9.1)	1 (4.4)	7 (15.9)	0.123
Hypertension, n (%)	1 (4.0)	3 (2.9)	3 (2.9)	8 (7.8)	0.027
**Co-medications, n (%)**					
Methotrexate	22 (88.0)	6 (54.6)	18 (78.3)	35 (79.6)	0.163
Leflunomide	14 (56.0)	2 (18.2)	4 (17.4)	2 (4.6)	< 0.001
HCQ	21 (84.0)	2 (18.2)	1 (4.4)	6 (13.6)	< 0.001
SSZ	2 (8.0)	2 (18.2)	0 (0.0)	1 (2.3)	0.087
Glucocorticoids	24 (96.0)	7 (63.6)	21 (91.3)	33 (75.0)	0.032
**Disease activity**					
DAS28	4.6 (4.1–5.4)	5.4 (4.8–6.4)	4.6 (4.0–5.2)	4.8 (4.0–5.2)	0.112
ESR, mm/hour	23 (14–57)	36 (18–60)	19 (11–29)	15 (7–30)	0.031
CRP, mg/dL	0.7 (0.2–2.3)	1.1 (0.3–2.8)	1.2 (0.2–2.1)	0.4 (0.1–1.7)	0.138

CRP, C-reactive protein; csDMARDs, conventional synthetic disease-modifying anti-rheumatic drugs; DAS28, disease activity score in 28 joints; DM, diabetic mellitus; ESR, erythrocyte sedimentation rate; HCQ, hydroxychloroquine; JAKi, janus kinase inhibitor; SSZ, sulfasalazine, TNFi, tumor necrosis factor α inhibitor

Supplemental References

1. Kim NH, Choi S, Han EJ, Hong BK, Choi SY, Kwon HM, et al. The xanthine oxidase-NFAT5 pathway regulates macrophage activation and TLR-induced inflammatory arthritis. *Eur J Immunol.* 2014;44(9):2721-36.
2. Johnson ZI, Shapiro IM, and Risbud MV. RNA Sequencing Reveals a Role of TonEBP Transcription Factor in Regulation of Pro-inflammatory Genes in Response to Hyperosmolarity in Healthy Nucleus Pulposus Cells: A HOMEOSTATIC RESPONSE? *J Biol Chem.* 2016;291(52):26686-97.
3. Buxadé M, Lunazzi G, Minguillón J, Iborra S, Berga-Bolaños R, Del Val M, et al. Gene expression induced by Toll-like receptors in macrophages requires the transcription factor NFAT5. *J Exp Med.* 2012;209(2):379-93.
4. Bischof RJ, Zafiropoulos D, Hamilton JA, and Campbell IK. Exacerbation of acute inflammatory arthritis by the colony-stimulating factors CSF-1 and granulocyte macrophage (GM)-CSF: evidence of macrophage infiltration and local proliferation. *Clin Exp Immunol.* 2000;119(2):361-7.
5. Wang EC, Newton Z, Hayward OA, Clark SR, Collins F, Perks WV, et al. Regulation of early cartilage destruction in inflammatory arthritis by death receptor 3. *Arthritis Rheumatol.* 2014;66(10):2762-72.
