# Supplementary material for: Serum amyloid A expression in liver promotes synovial macrophage activation and chronic arthritis via NFAT5
Source: J Clin Invest. 2024 Mar 1;134(5):e167835. doi: 10.1172/JCI167835 (PMC10904059; doi:10.1172/JCI167835)
Supplement: Unedited blot and gel images [file jci-134-167835-s126.pdf]

Uncropped blot for Figure 1A (upper panel)

Anti-NFAT5

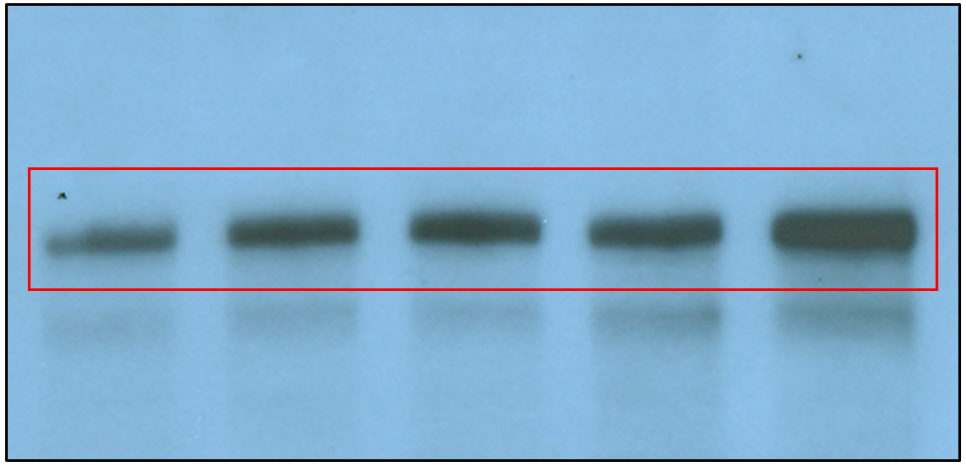

Anti-β-actin

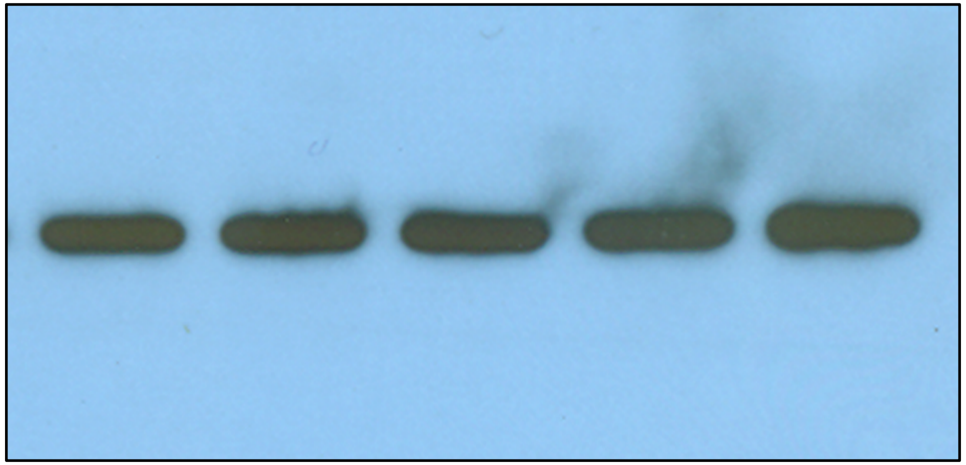

Uncropped image of the Western blot shown in Figure 1A (upper panel).

Uncropped blot for Figure 1A (lower panel)

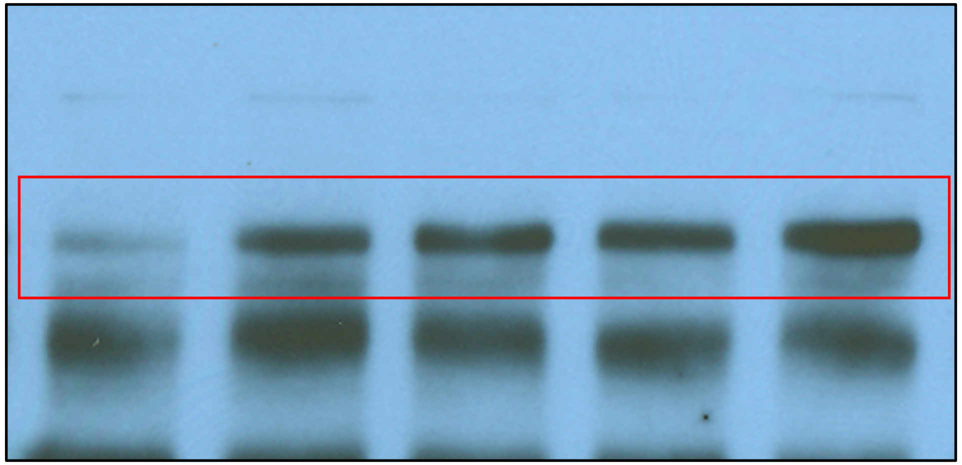

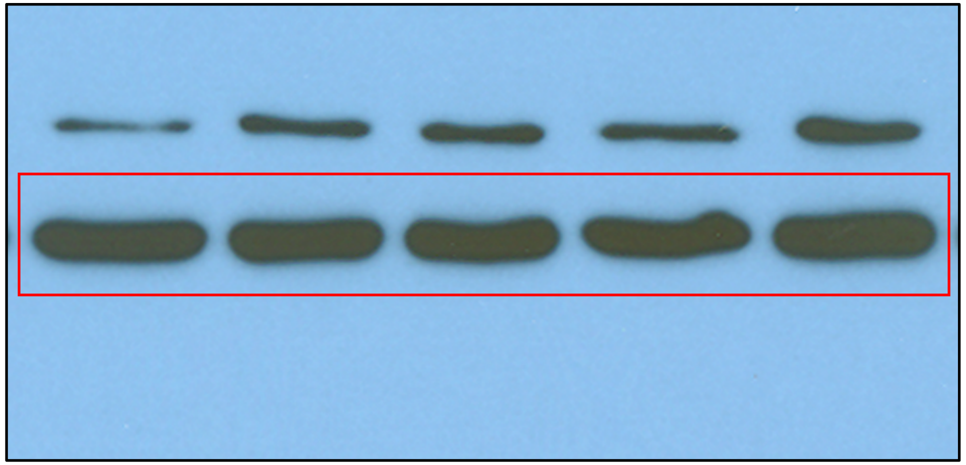

Uncropped image of the Western blot shown in Figure 1A (lower panel).

Uncropped blot for Figure 1B

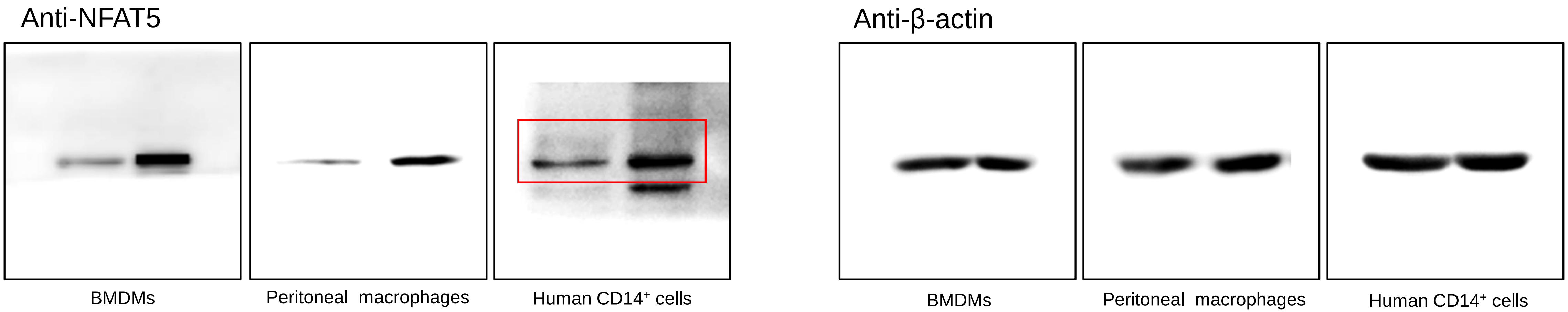

Uncropped image of the Western blot shown in Figure 1B.

Uncropped blot for Figure 2A

Anti-NFAT5

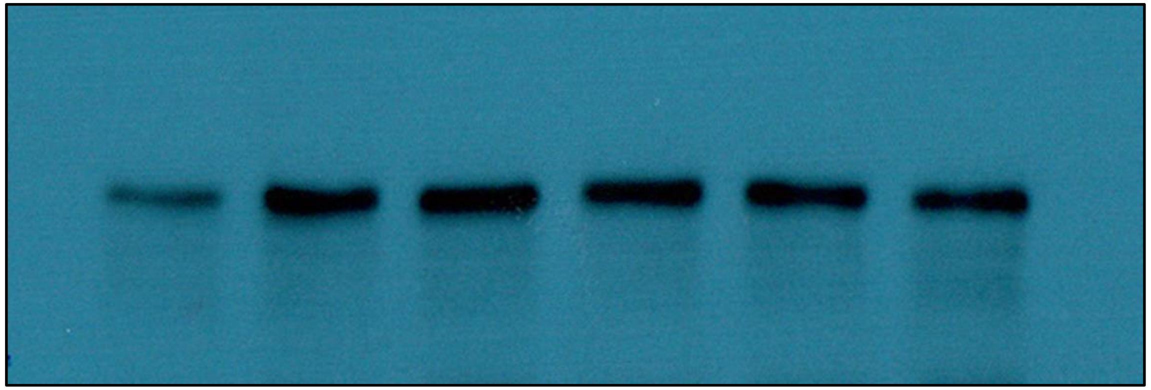

Anti-β-actin

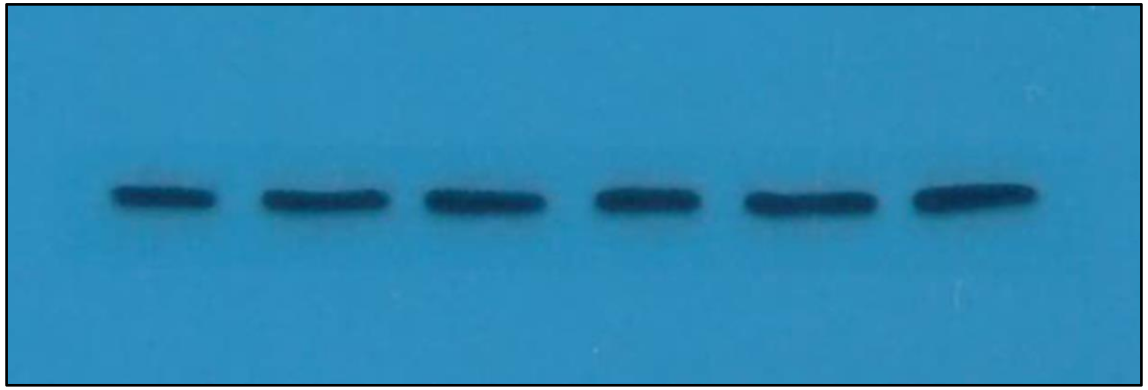

Uncropped image of the Western blot shown in Figure 2A.

Uncropped blot for Figure 2B

Anti-NFAT5

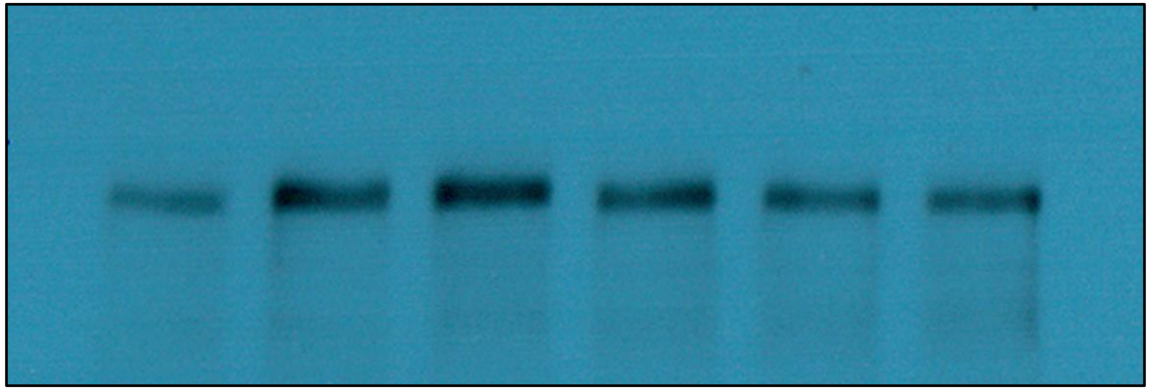

Anti-β-actin

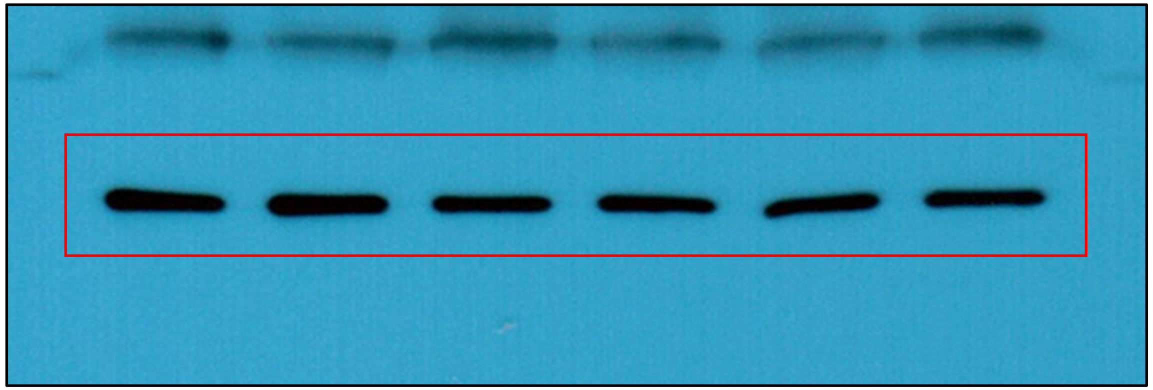

Uncropped image of the Western blot shown in Figure 2B.

Uncropped blot for Figure 2C

Anti-NFAT5

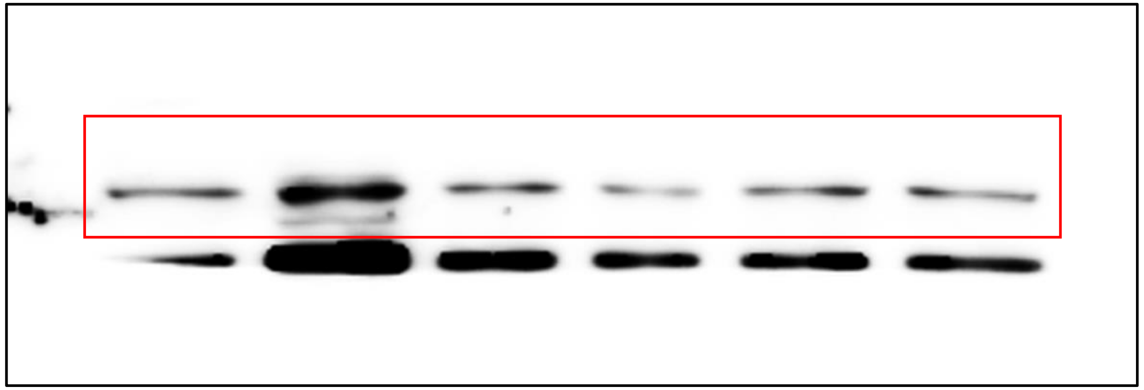

Anti-β-actin

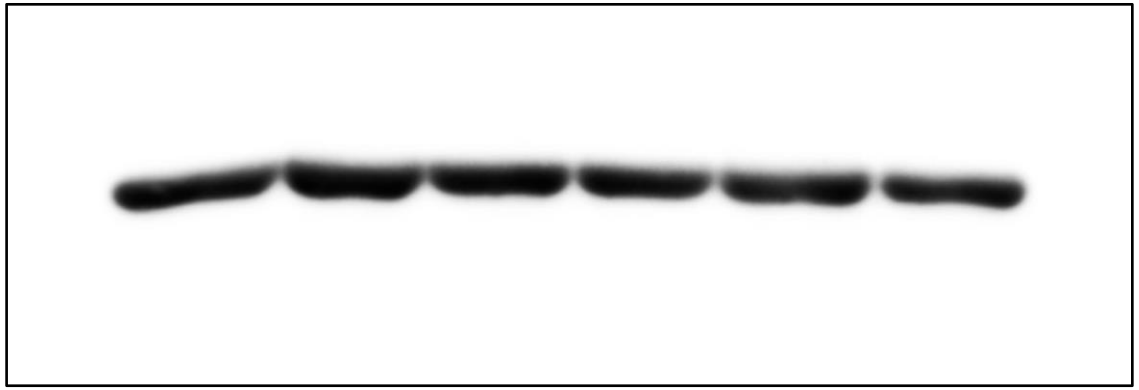

Uncropped image of the Western blot shown in Figure 2C.

Uncropped blot for Figure 2D

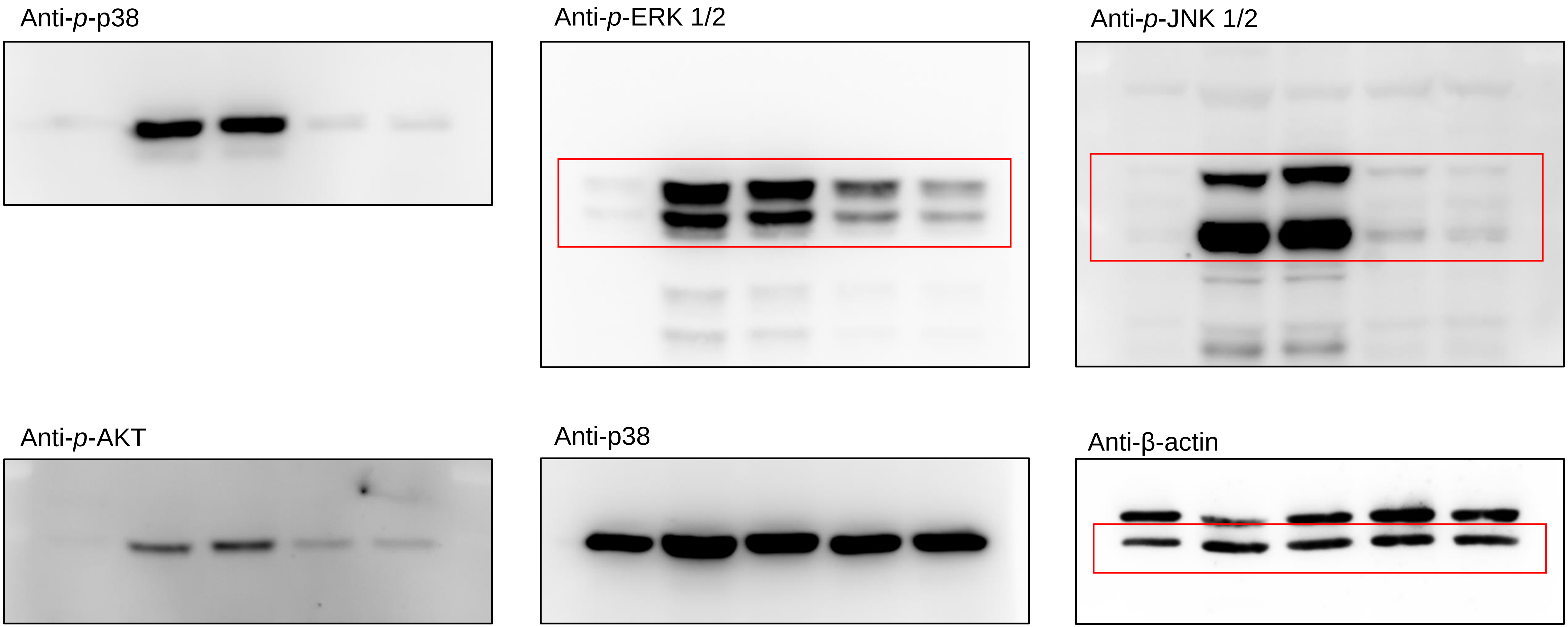

Uncropped image of the Western blot shown in Figure 2D.

Uncropped blot for Figure 2E

Anti-*p*-p38

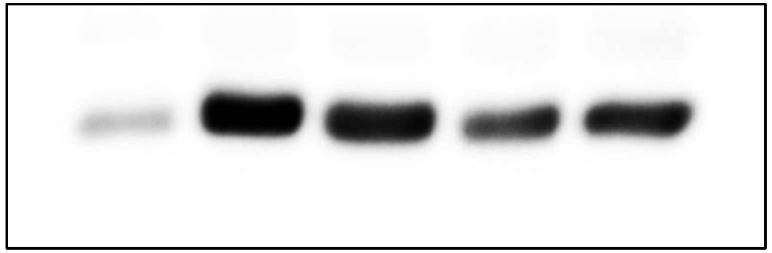

Anti-*p*-ERK 1/2

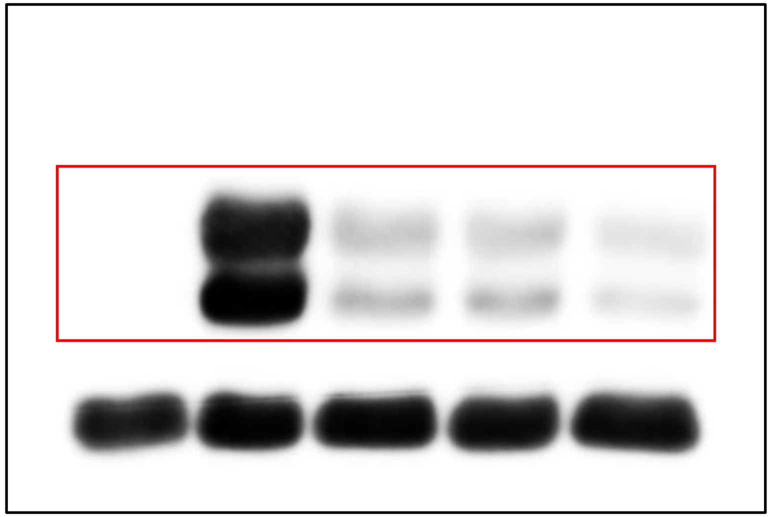

Anti-*p*-JNK 1/2

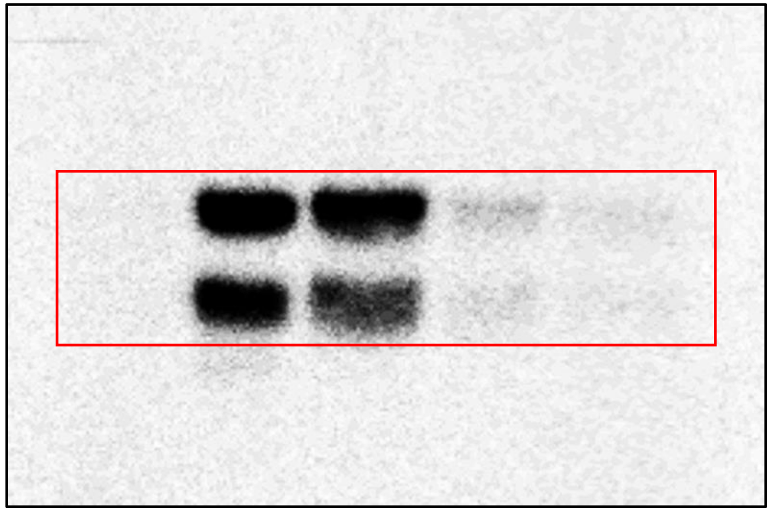

Anti-*p*-AKT

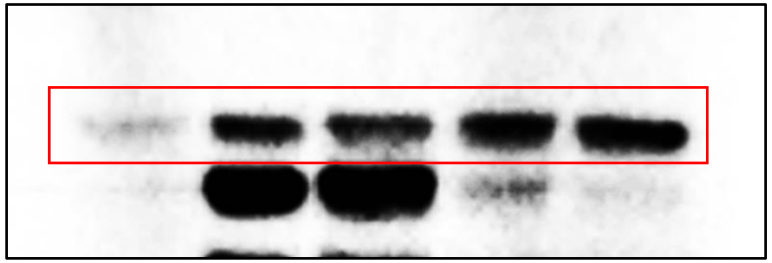

Anti-AKT

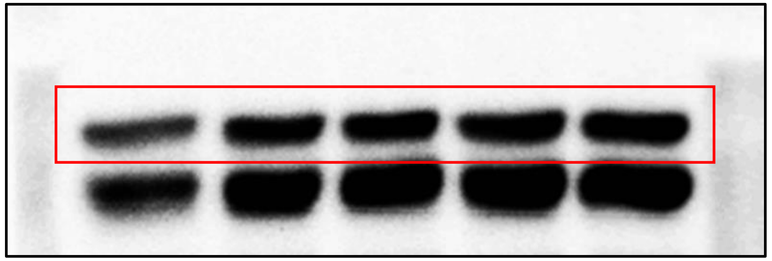

Anti-GAPDH

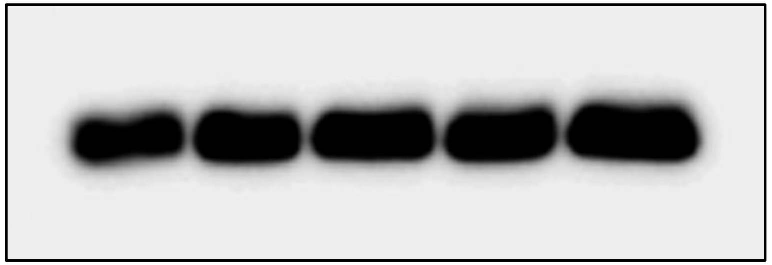

Uncropped image of the Western blot shown in Figure 2E.

Uncropped blot for Figure 2F

Anti-NFAT5

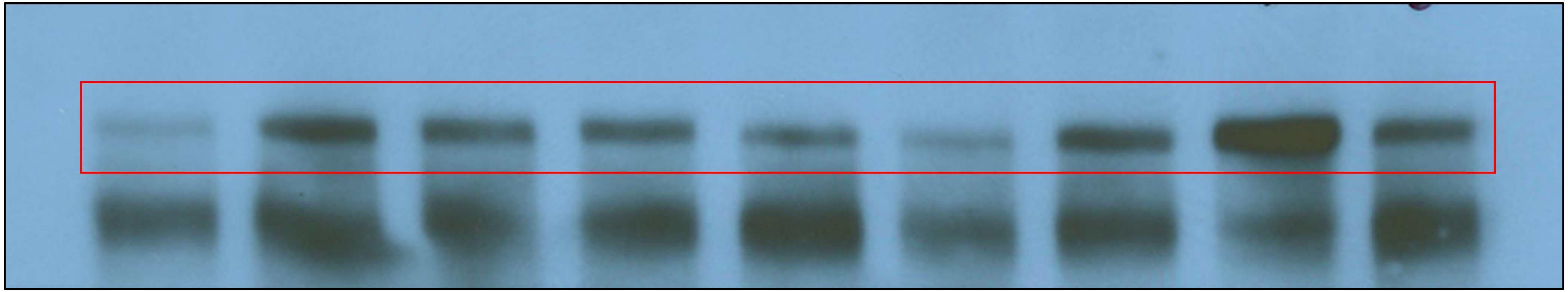

Anti-β-actin

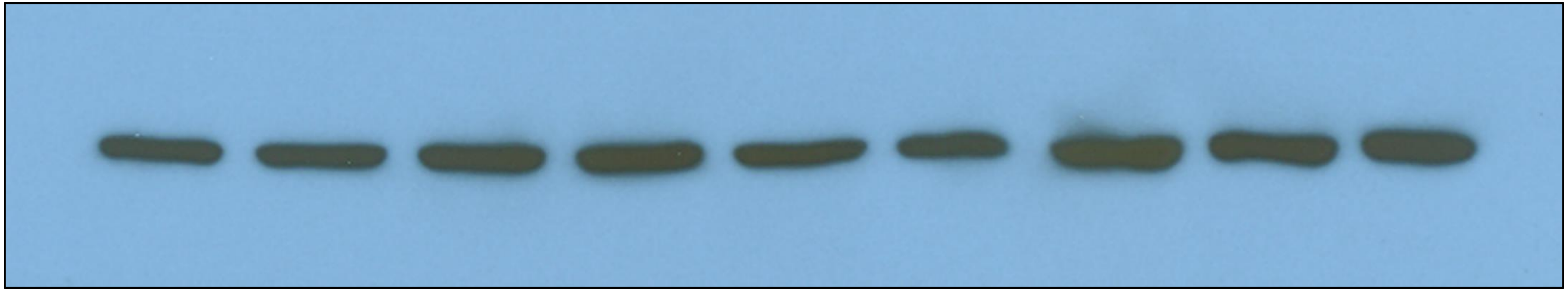

Uncropped image of the Western blot shown in Figure 2F.

Uncropped blot for Figure 2G

Anti-NFAT5

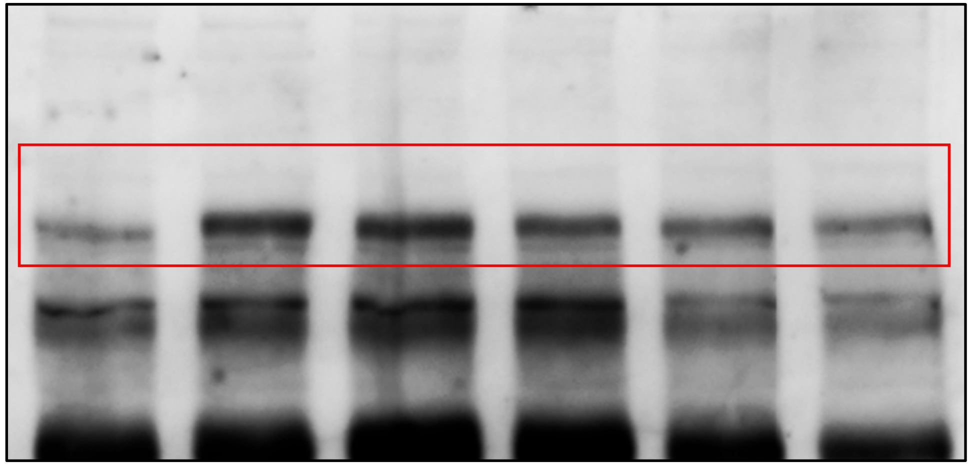

Anti-GAPDH

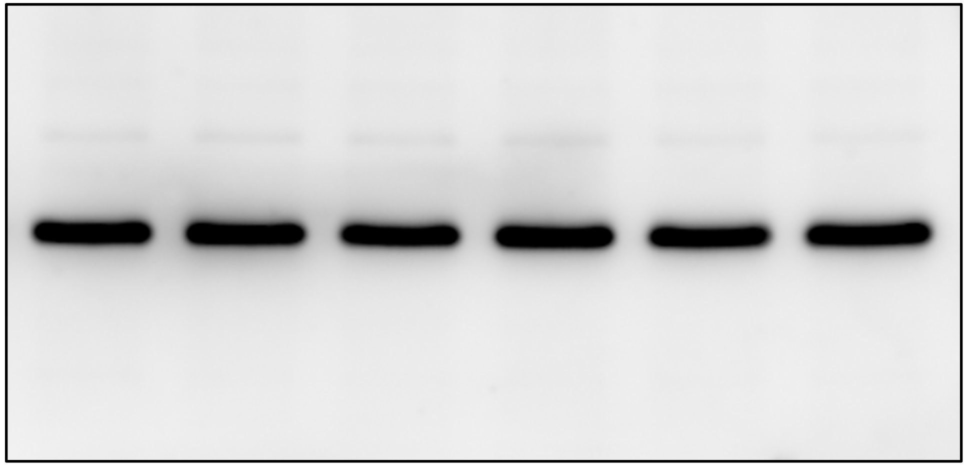

Uncropped image of the Western blot shown in Figure 2G.

Uncropped blot for Figure 2H

Anti-NFAT5

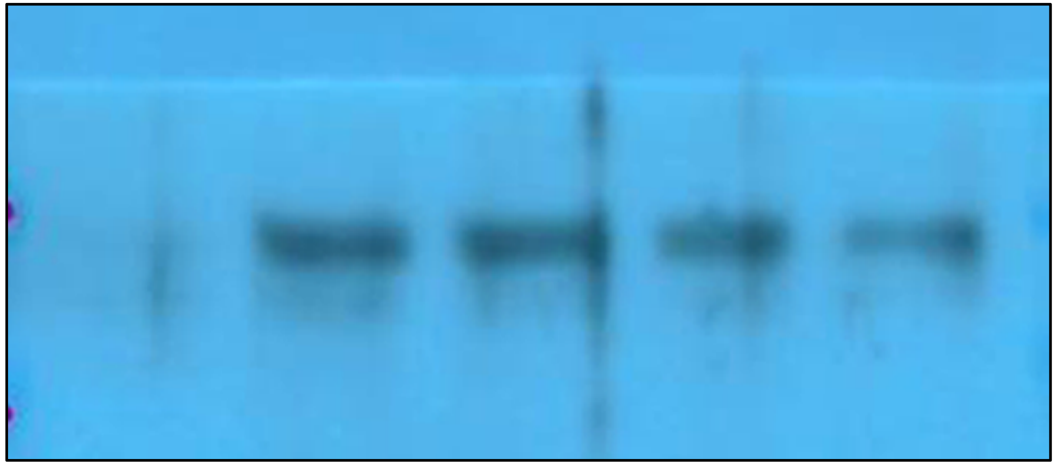

Anti-β-actin

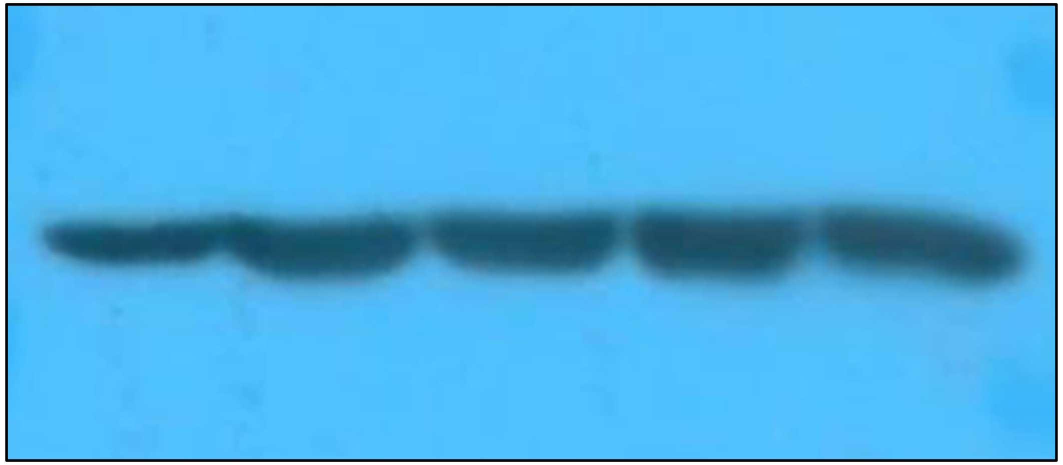

Uncropped image of the Western blot shown in Figure 2H.

Uncropped blot for Figure 2I

Anti-NFAT5

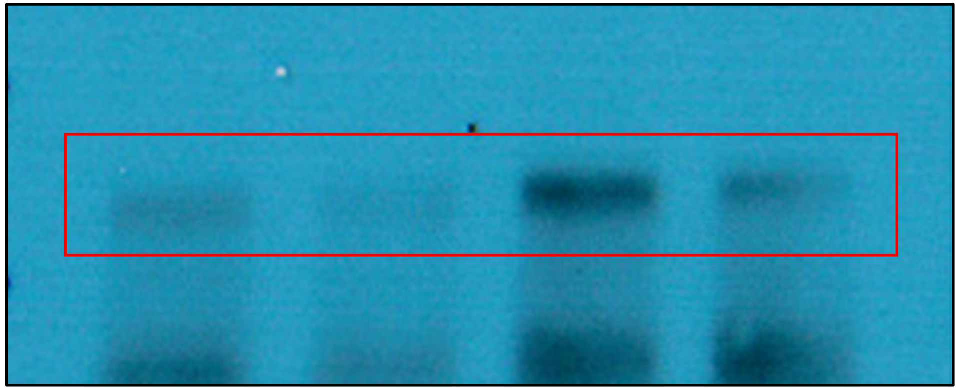

Anti-β-actin

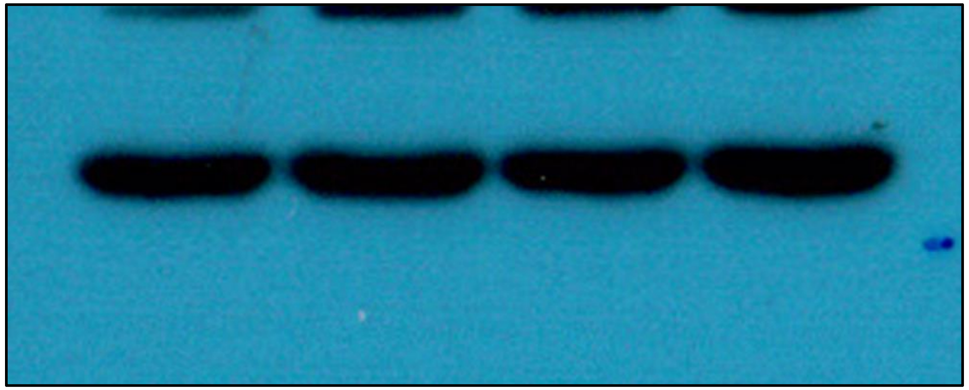

Anti-Total JNK1/2

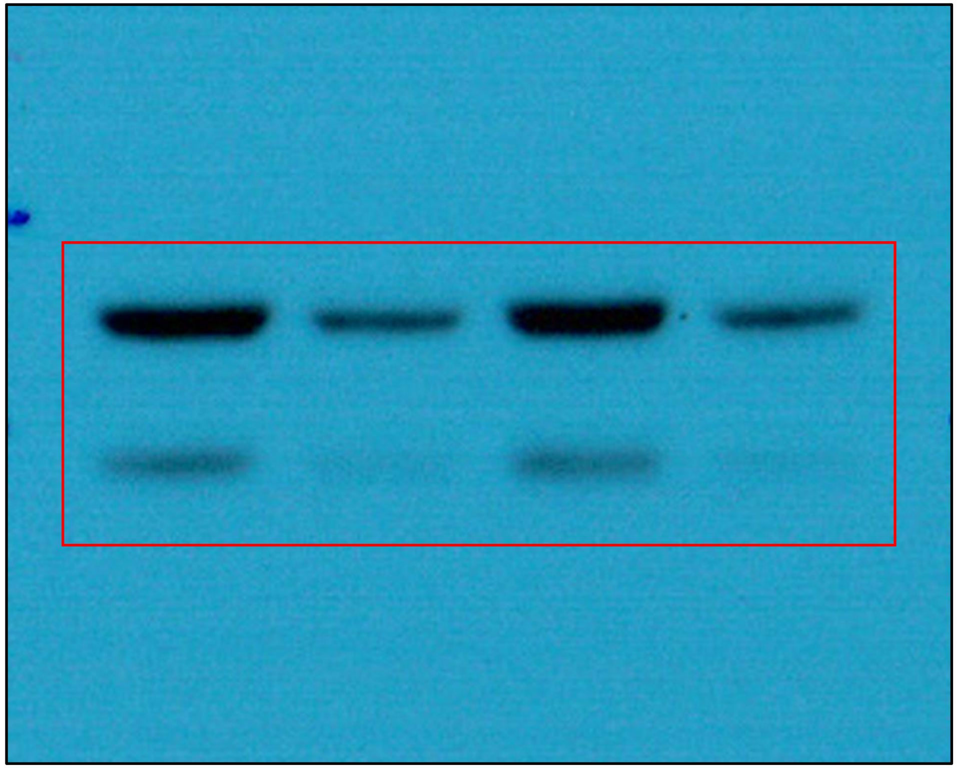

Uncropped image of the Western blot shown in Figure 2I.

Uncropped blot for Figure 2J

Anti-*p*-JNK 1/2

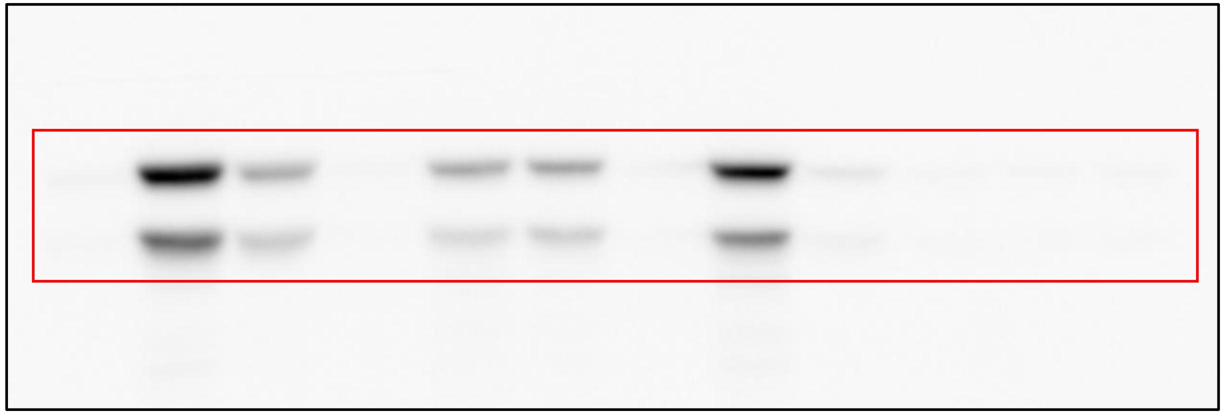

Anti-JNK1/2

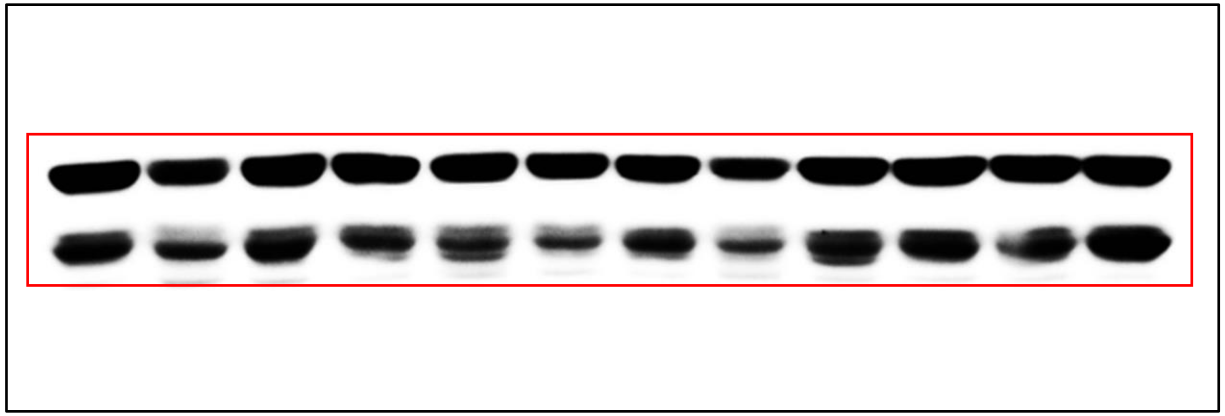

Anti-β-actin

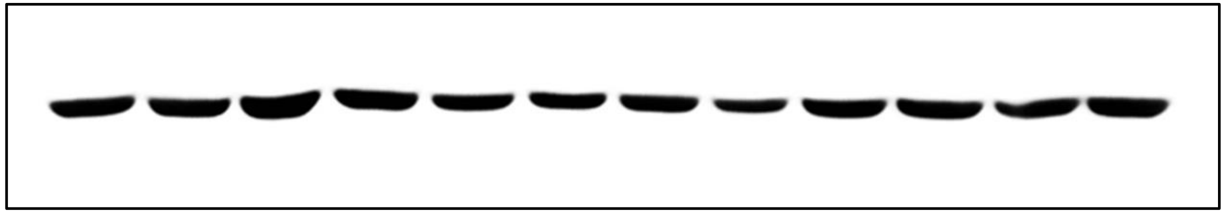

Uncropped image of the Western blot shown in Figure 2J.

Uncropped blot for Supplemental Figure 1A

Anti-NFAT5

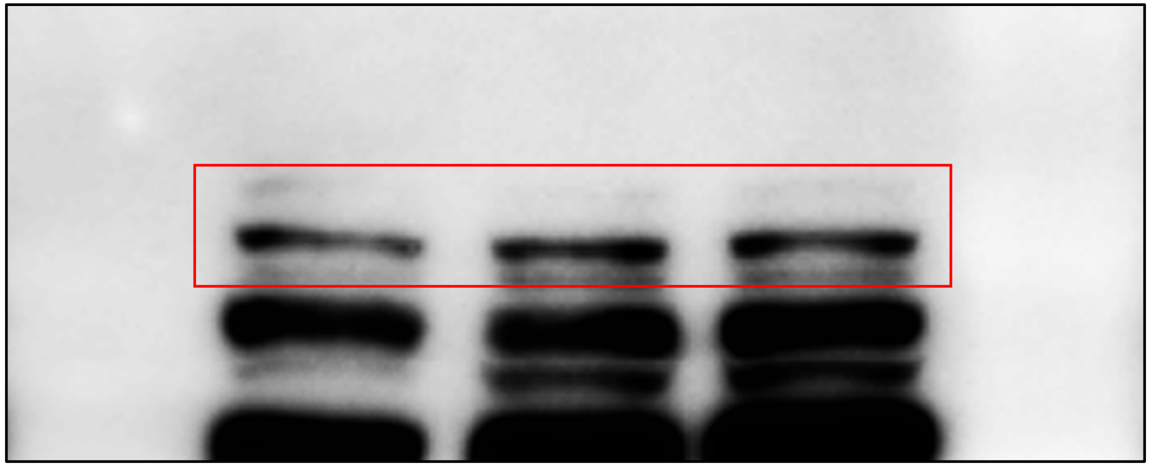

Anti-GAPDH

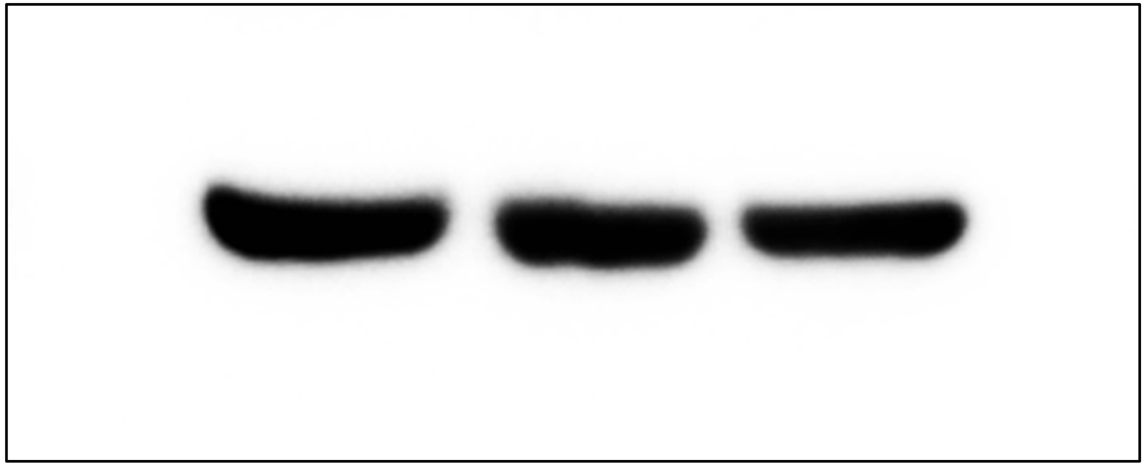

Uncropped image of the Western blot shown in Supplemental Figure 1A.

Uncropped blot for Supplemental Figure 1B

Anti-NFAT5

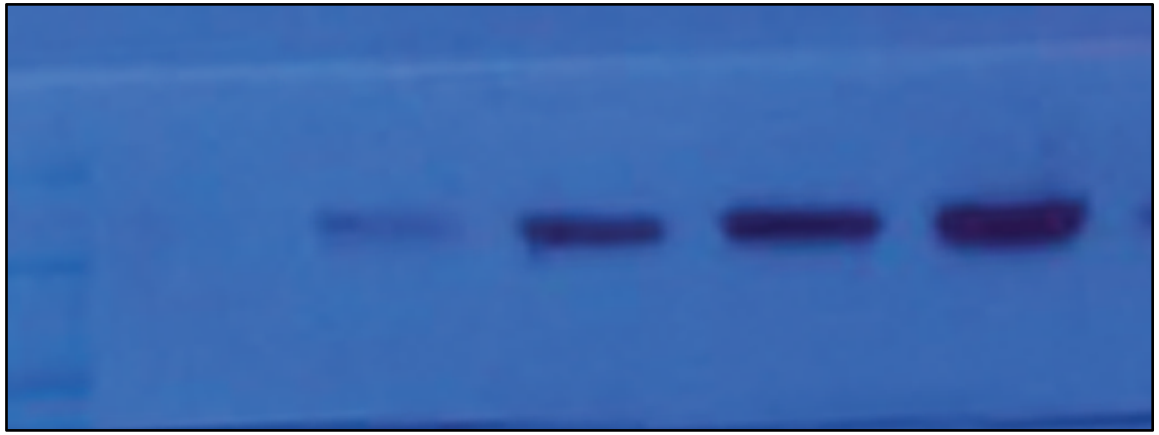

Anti-GAPDH

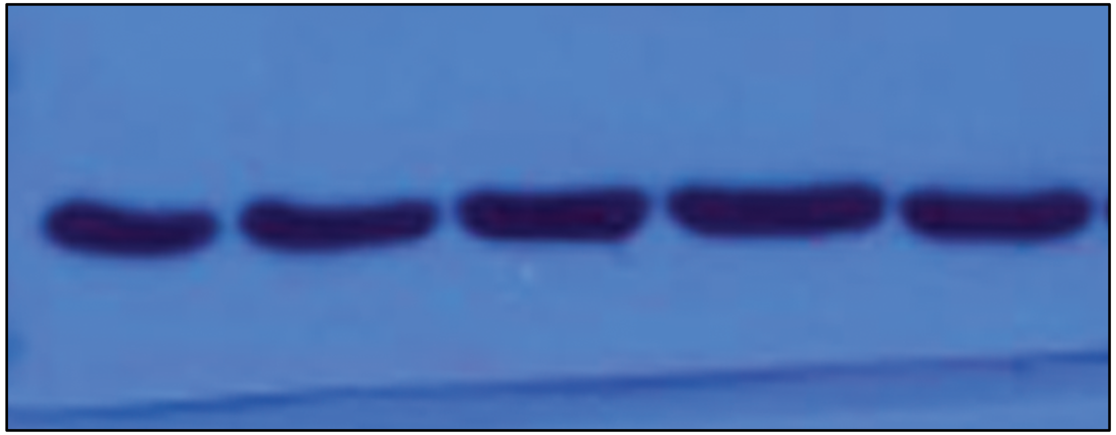

Uncropped image of the Western blot shown in Supplemental Figure 1B.

Uncropped blot for Supplemental Figure 3A

Anti-NFAT5

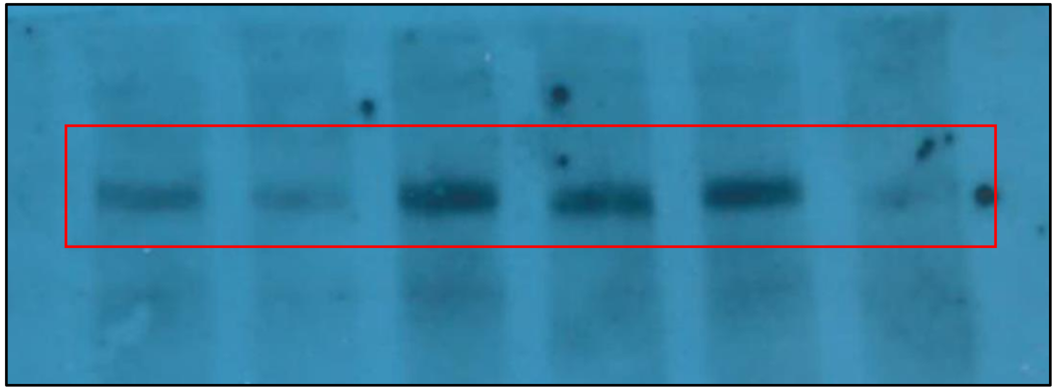

Anti-β-actin

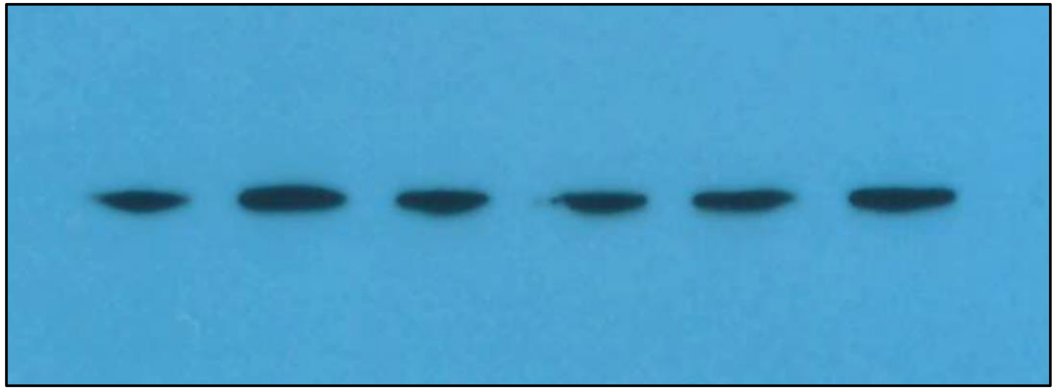

Uncropped image of the Western blot shown in Supplemental Figure 3A.

Uncropped blot for Supplemental Figure 3B

Anti-NFAT5

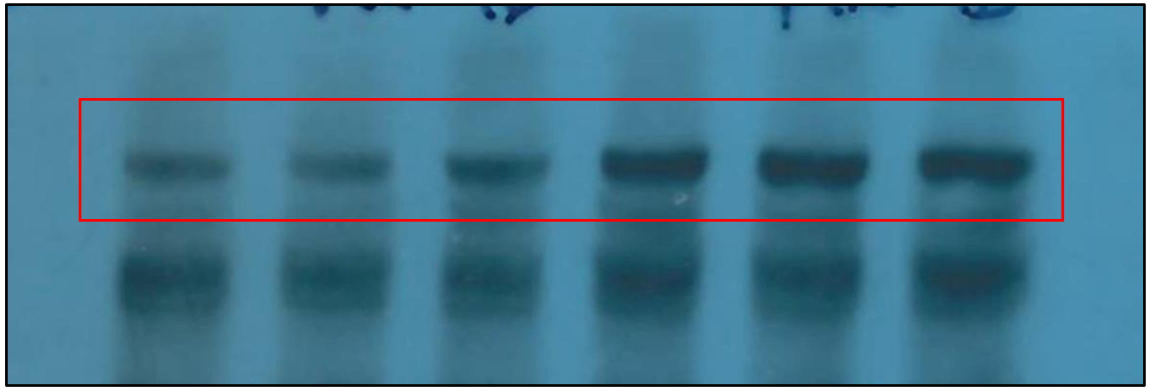

Anti-β-actin

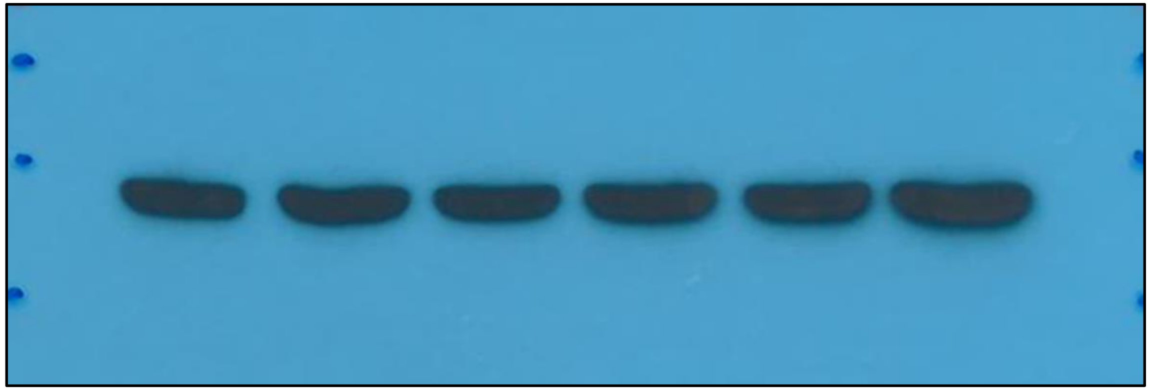

Uncropped image of the Western blot shown in Supplemental Figure 3B.

Uncropped blot for Supplemental Figure 3C

Anti-NFAT5

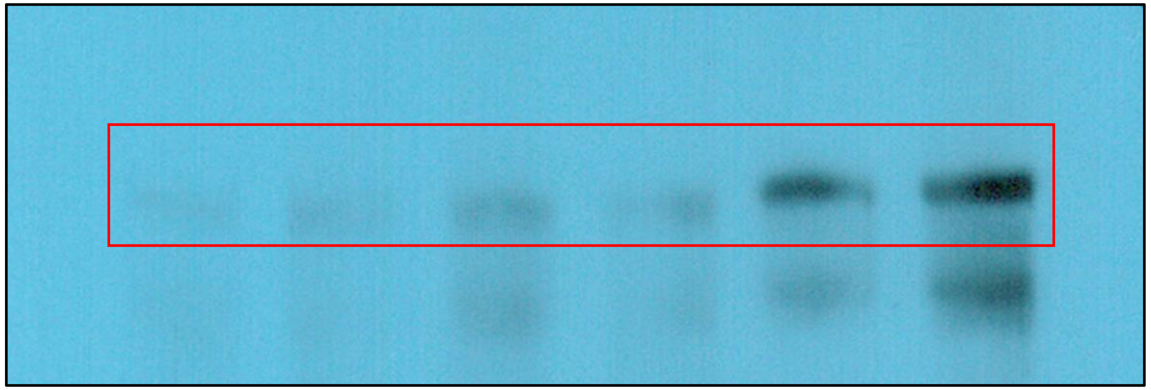

Anti-β-actin

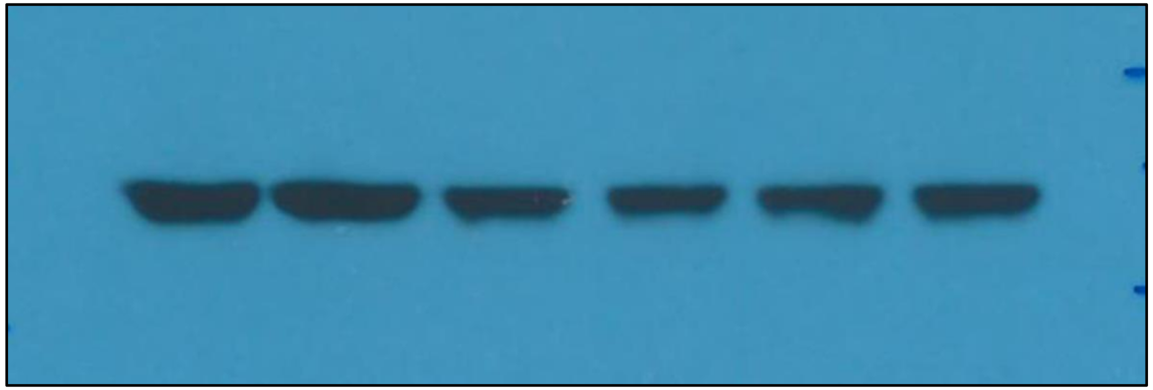

Uncropped image of the Western blot shown in Supplemental Figure 3C.

Uncropped blot for Supplemental Figure 4A

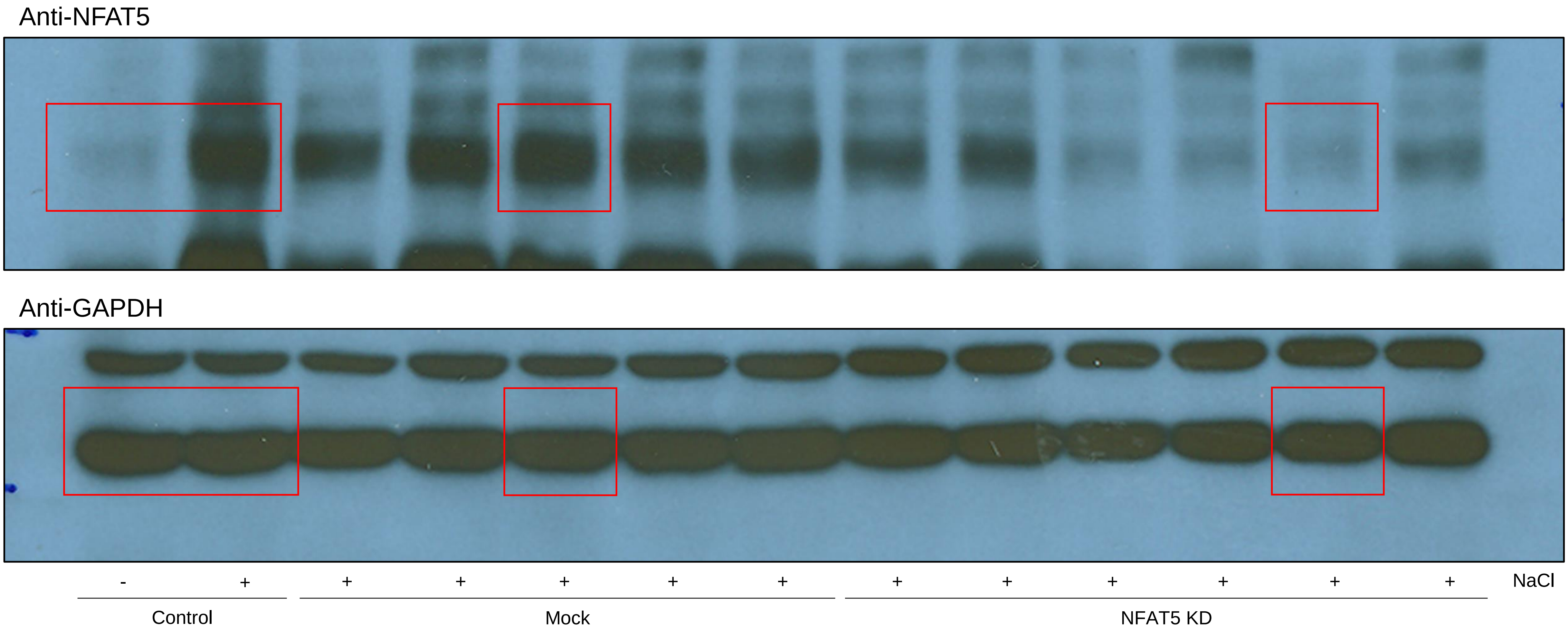

Uncropped image of the Western blot shown in Supplemental Figure 4A.

Uncropped blot for Supplemental Figure 4B

Anti-NFAT5

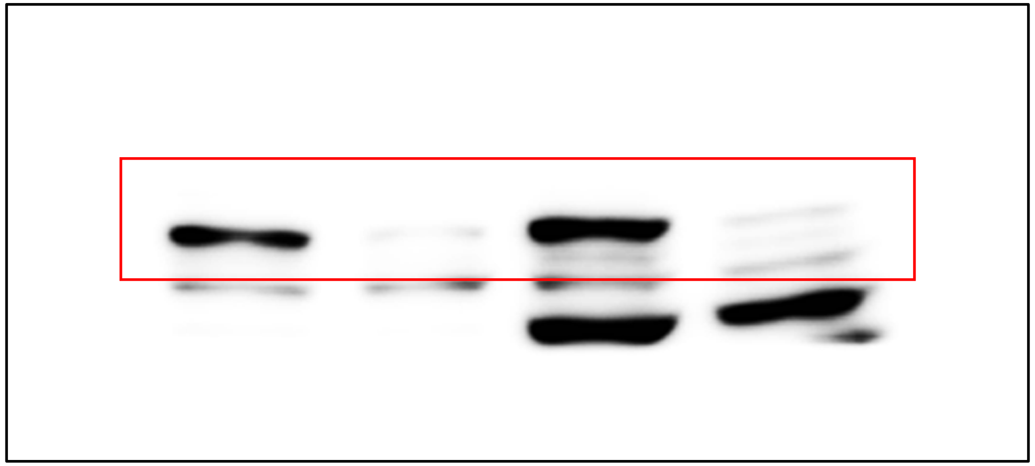

Anti-β-actin

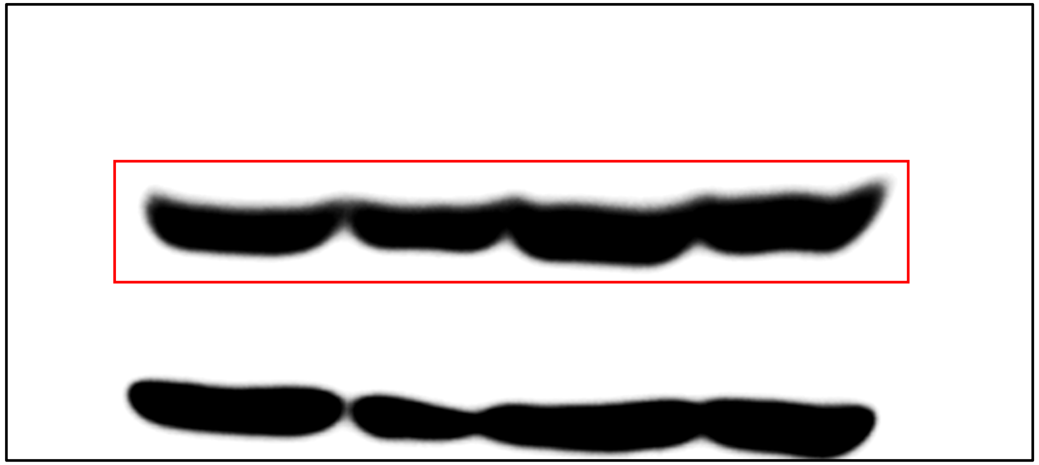

Uncropped image of the Western blot shown in Supplemental Figure 4B.

Uncropped blot for Supplemental Figure 4C

Anti-NFAT5

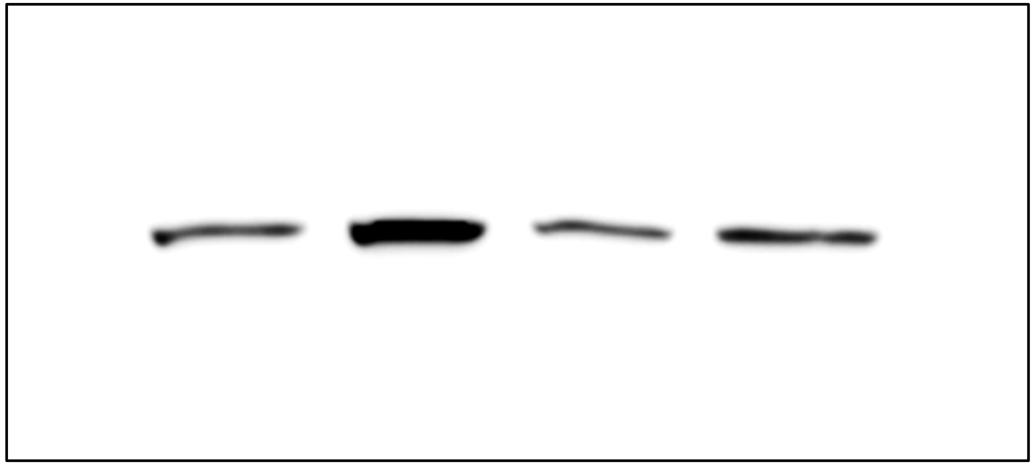

Anti-β-actin

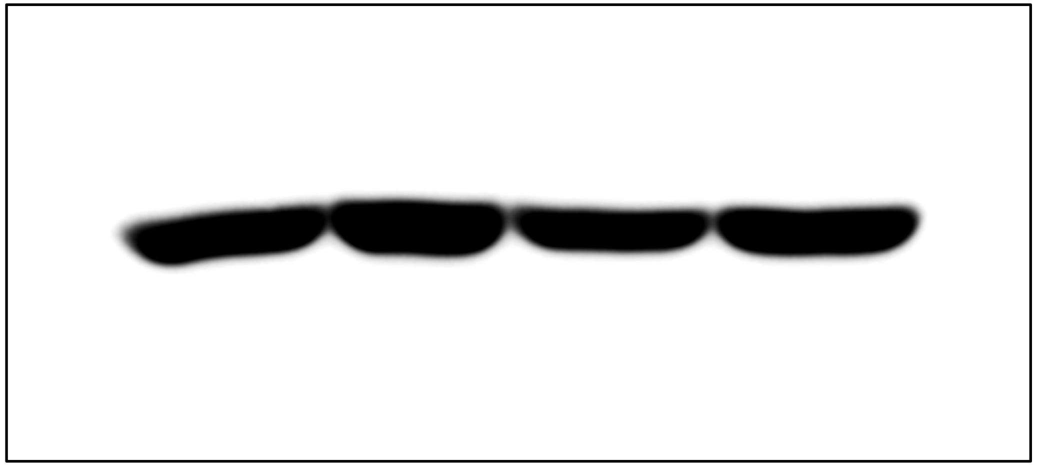

Uncropped image of the Western blot shown in Supplemental Figure 4C.

Uncropped blot for Supplemental Figure 4D

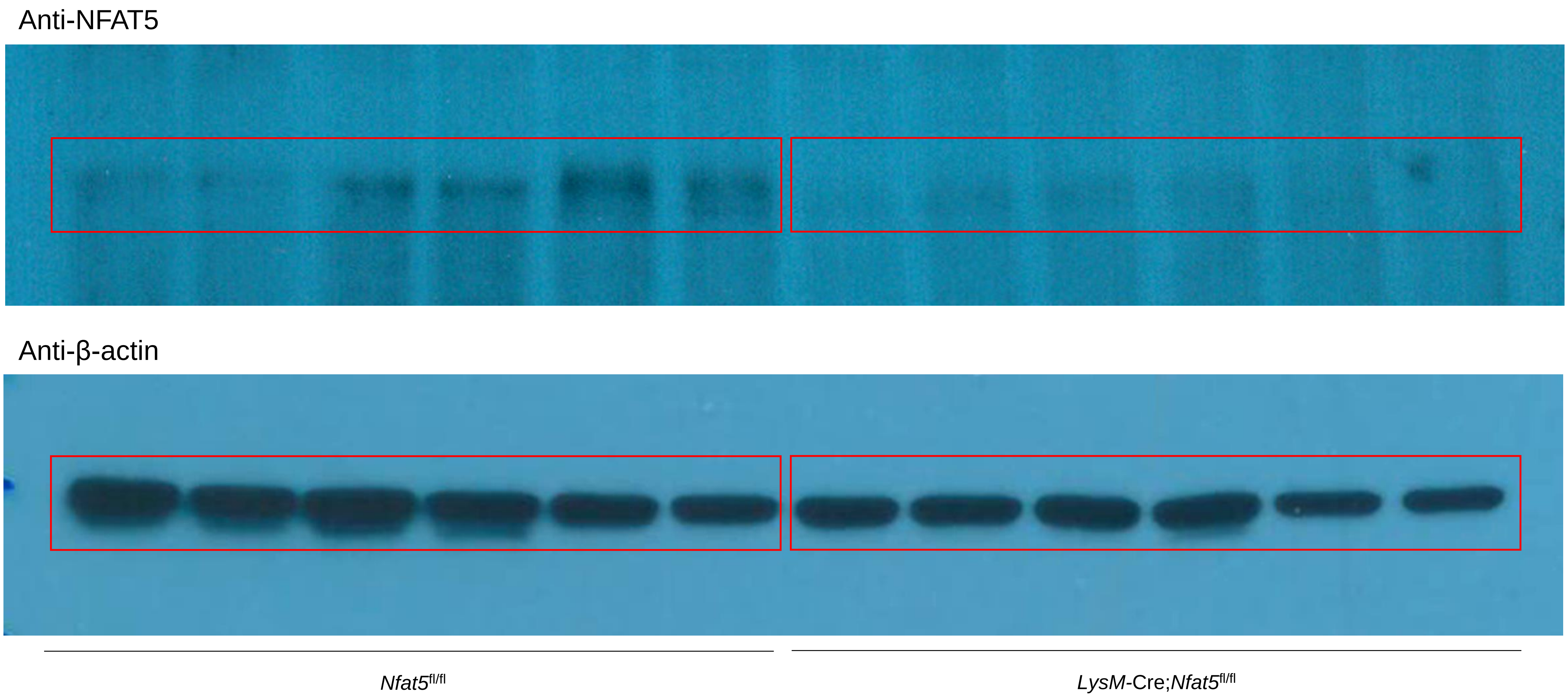

Uncropped image of the Western blot shown in Supplemental Figure 4D.
